# CCMAlnc Promotes the Malignance of Colorectal Cancer by Modulating the Interaction Between miR-5001-5p and Its Target mRNA

**DOI:** 10.3389/fcell.2020.566932

**Published:** 2020-12-16

**Authors:** Yuqing Yan, Baoqin Xuan, Ziyun Gao, Chaoqin Shen, Yingying Cao, Jie Hong, Haoyan Chen, Zhe Cui, Guangyao Ye, Jing-Yuan Fang, Zhenhua Wang

**Affiliations:** ^1^State Key Laboratory of Oncogenes and Related Genes, Ren Ji Hospital, School of Medicine, Shanghai Jiao Tong University, Shanghai, China; ^2^Key Laboratory of Gastroenterology & Hepatology, Ministry of Health, Ren Ji Hospital, School of Medicine, Shanghai Jiao Tong University, Shanghai, China; ^3^Division of Gastroenterology and Hepatology, Ren Ji Hospital, School of Medicine, Shanghai Jiao Tong University, Shanghai, China; ^4^Shanghai Cancer Institute, Ren Ji Hospital, School of Medicine, Shanghai Jiao Tong University, Shanghai, China; ^5^Shanghai Institute of Digestive Disease, Ren Ji Hospital, School of Medicine, Shanghai Jiao Tong University, Shanghai, China; ^6^Department of Gastrointestinal Surgery, Ren Ji Hospital, School of Medicine, Shanghai Jiao Tong University, Shanghai, China

**Keywords:** CCMAlnc, miR-5001-5p, HES6, colorectal cancer, metastasis

## Abstract

**Objective:**

Colorectal cancer (CRC) is highly malignant and cancer metastasis remains the predominant cause of CRC death. The potential molecular mechanism of long non-coding RNA (lncRNAs) in CRC malignance is still poorly elucidated.

**Methods:**

CCMAlnc expression was analyzed by using the Sequence ReadArchive (SRA) database. Target gene expression was examined by real-time PCR and Western blotting. The biological function of CCMAlnc and miR-5001-5p was detected by cell invasion, CCK8 proliferation, and colony formation assays in loss of function and gain of function experiments *in vitro*. A luciferase assay was performed to validate the target site of miR-5001-5p on the 3′-UTR of HES6 mRNA.

**Results:**

CCMAlnc was identified as a novel functional lncRNA in CRC. Elevated CCMAlnc was detected in CRC cells as well as in clinical CRC tissue samples, and the expression of this lncRNA positively correlated with the poor prognosis of CRC patients. Functional validation assays revealed that downregulation of CCMAlnc impaired CRC cell proliferation and invasion *in vitro*, but upregulation of CCMAlnc reversed this effect. Moreover, CCMAlnc was validated to act as a competing endogenous RNA (ceRNA) that stabilizes the expression of HES6 by downregulating miR-5001-5p.

**Conclusion:**

CCMAlnc/miR-5001-5p/HES6 signaling is strongly activated to promote CRC malignance. CCMAlnc is defined as a potential candidate biomarker for metastasis prediction in CRC patients and as a potential therapeutic target for CRC treatment.

## Introduction

Despite the increasing awareness of cancer, CRC continues to be one of the most commonly diagnosed cancers and the third leading cause of cancer-related death (Cronin et al., 2018; Siegel et al., 2020). CRC is highly malignant and prone to metastasis, with approximate metastatic rates in patients at initial diagnosis and confirmed diagnosis of 25% and 50%, respectively (Van Cutsem et al., 2010; Tauriello et al., 2017). Resection of both primary and metastatic tissues provides the best prognosis for CRC patients with metastasis, but post-intervention recurrence is very common on account of disseminated latent or therapy-resistant tumor cells (Benson et al., 2018).

With the rapid development of genetic knowledge and technologies, some signatures concerning CRC malignance have been discovered (Schell et al., 2016; Sakai et al., 2018; Lee et al., 2019; Song et al., 2019). However, the CRC mortality rate is still high in countries that have increasing incidence rates and limited resources. Novel diagnostic and treatment signatures that accurately recapitulate the process of CRC malignance are still required.

Human cells utilize only 2% of their genome to generate transcripts with protein-coding sequences. A large portion of the genome is transcribed into non-coding RNAs (ncRNAs) with no apparent protein-coding potential. Non-coding transcripts larger than 200 nucleotides are defined as lncRNAs (Ponting et al., 2009). Aberrant expression of lncRNA plays important roles in the progression of CRC (Kogo et al., 2011; Xu M. et al., 2018; Ji et al., 2019; Xu et al., 2019; Yang et al., 2019). At the molecular level, lncRNAs adopt different mechanisms to regulate chromatin organization, gene transcription, and posttranscriptional RNA processing (Mercer et al., 2009). Most commonly, lncRNAs are described to compete for miRNA binding, thereby modulating the derepression of miRNA targets.

In the present study, we discovered that multiple lncRNAs, including CCMAlnc, were differentially expressed among CRC tissues and adjacent normal mucosa. The level of CCMAlnc was upregulated from normal colon tissues to cancer samples to metastatic samples and had vital cell function in CRC malignance. Furthermore, we found that CCMAlnc reduced the expression of target gene by competing with miR-5001-5p, which mechanistically and pathologically contributed to CRC malignance. The target gene was hairy and enhancer of split family basic helix-loop-helix transcription factor 6 (HES6), a member of the HES family, and has been implicated in many cancers (Xu Y. et al., 2018).

## Materials and Methods

### Clinical Specimens

Fresh CRC tissues and normal colorectal mucosa from 53 CRC patients who underwent surgery at Renji Hospital from 2016 to 2019 were collected. The clinical characteristics including gender, age, tumor size and etc. of patients was provided in Supplementary Table S1. This study was approved by the ethics committee of Shanghai Jiao Tong University School of Medicine, Renji Hospital, and the protocol was approved by the Ethics Committee of [2015]097. Written informed consent was obtained from all participants in this study.

### Data Collection

The RNA-Sequencing (RNA-Seq) raw data were downloaded from the SRA repository with the access numbers PRJNA218851, PRJNA376161, and PRJNA411984. In PRJNA218851 dataset, RNA-seq data of 54 samples (normal colon, primary CRC and liver metastasis) were generated from 18 CRC patients. In PRJNA376161 dataset, 10 pairs primary tumor and matching normal colon tissue, five pairs aberrant crypt foci and matching normal crypt were included. In PRJNA411984 dataset, whole-transcriptome was analyzed in six total samples consisting of three pairs of CRC and matched normal mucosa.

### Bioinformatics Analysis

Data analysis was performed according to the TopHat-HTSeq-DeSeq2 frame (Anders et al., 2013). Gene set enrichment analysis (GSEA) was performed by calculating the overlap of gene sets of interest with annotated gene sets stored in the Molecular Signatures Database (MSigDB), version 6.0 (Broad Institute, Cambridge, MA, United States). The expression level of CCMAlnc was used as a phenotype label, and the “metric for ranking genes” was set to the Pearson correlation.

### Cell Lines and Cell Cultures

Human CRC cell lines SW1116, SW480, Caco2, Lovo, HT29, and HCT116 were purchased from ATCC. SW1116, SW480, and Lovo were cultured using RPMI-1640 medium with 10% FBS. HT29 and HCT116 were cultured using Macoy’s 5A medium with 10% FBS. Caco2 cells were cultured in DMEM with 20% FBS. All cells were cultured at 37°C in a humidified 5% CO_2_ atmosphere.

### RNA Extraction and Quantitative Real-Time PCR

Total RNA was extracted using the RNAiso Plus (Takara, Japan) from the cultured cells and was reverse transcribed to complementary DNA (cDNA) using the PrimeScript RT Reagent Kit (Takara, Japan). For miRNA analysis, first-strand cDNA was synthesized using the Mir-XTM miRNA First-Strand Synthesis Kit (Takara, Japan). Real-time polymerase chain reaction (RT-PCR) was performed using the Takara SYBR Green PCR Kit (Takara, Japan) according to the manufacturer’s protocol. All reactions were run on an Applied Biosystems 7900 Quantitative PCR System (Applied Biosystems). The Ct values obtained from different samples were compared using the 2^–ΔΔCt^ method. GAPDH and U6 were used as endogenous controls. The primers are listed as follows:

CCMAlnc-Forward: CGAGGGTCAACATCTTGTGAG; CCMAlnc-Reverse: GTTAAGCATCTTGCCCAAGGT; HES6- Forward: GGCAGCAGCTTCCAGGATCTG; HES6-Reverse: AGGTCGGAGCACAGGTCGTC; GAPDH-Forward: GCAT TGCCCTCAACGACCAC; GAPDH-Reverse: CCACCACCC TGTTGCTGTAG.

### Cell Migration and Invasion Assays

Transwell assays were performed in 6.5 mm diameter Boyden chambers with pore size of 8.0 mm (Corning, NY, United States). For migration assays, the stable monoclonal CRC cells (2^∗^10^5^ cells per well) were resuspended in migration medium (medium without FBS) and placed in the upper compartment of transwell chamber coated with fibronectin on the lower surface. The lower compartment was filled with 600 μl medium containing 20% FBS as a chemoattractant. After incubation for 72 h at 37°C with 5% CO_2_ in a humidified incubator, cells on the lower surface of the filter were fixed in 4% formaldehyde for 20 min and stained with 0.1% crystal violet.

### Western Blotting Assay

The whole protein was extracted, separated by 12% SDS-PAGE gel and transferred to a PVDF membrane. HES6 was detected with a monoclonal anti-HES6 antibody (1:200, sc-133196, Santa Cruz, United States). GAPDH (1:5000, KC-5G5, Kangcheng, China) expression was used as an equal loading control. The secondary antibody was anti-mouse IgG-HRP (1:3,000, KC-RB-035, Kangcheng, China).

### Statistical Analysis

All statistical analyses were performed in R-3.0.2^1^. The difference in expression levels between tumors and matched non-tumor tissues were analyzed using a paired Student’s *t*-test. The mean expression values of any two preselected groups were compared using an independent Student’s *t*-test. The Kaplan–Meier method was used to plot the survival curves, and the log-rank test was used to assess survival differences. *P*-values of < 0.05 and < 0.01 were considered statistically significant and highly statistically significant, respectively.

## Results

### Differential lncRNA Expression Was Associated With CRC Metastasis in SRA

To elucidate the driving molecular mechanism of CRC metastasis, we hypothesized that specific lncRNA signatures targeted the genes involved in cancer malignance. To test this hypothesis, we analyzed the lncRNA data on CRC from the SRA database^2^.

**FIGURE 1:**
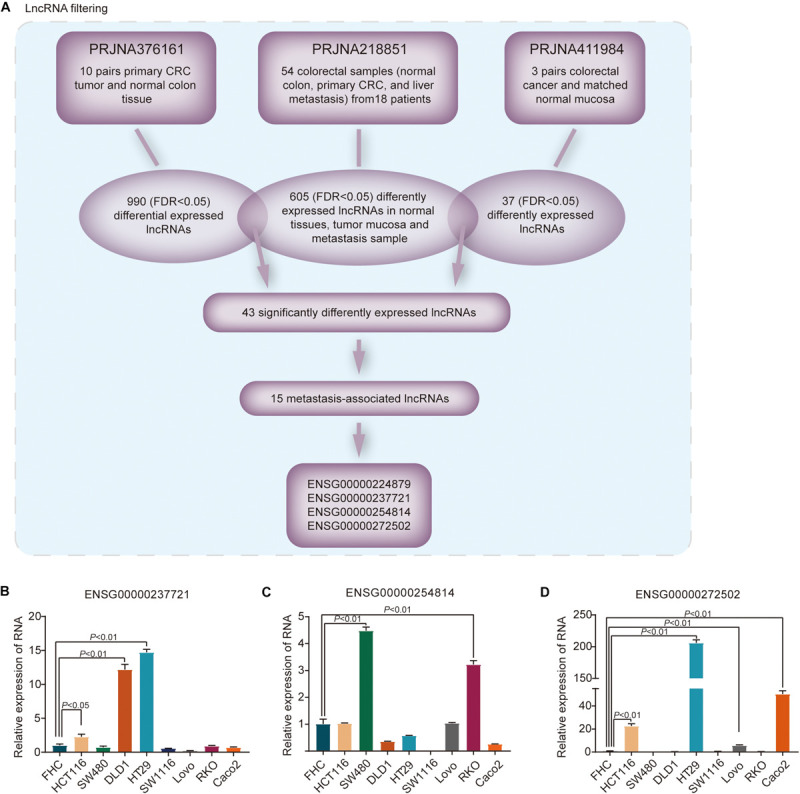
Differential lncRNAs in metastasis-associated CRC in the SRA database. **(A)** Identification of the differential expression of lncRNAs in metastatic and primary CRC tissues compared with normal mucosa from the SRA patient cohort. FDR < 0.05. **(B–D)** The relative levels of three candidate lncRNAs were measured in normal colon epithelial cells and eight CRC cell lines.

In PRJNA218851 dataset, 605 differentially expressed genes were identified, with an FDR < 0.05, among normal mucosa, cancer samples and metastasis tissues. We further overlapped these lncRNAs with lncRNAs from the other two datasets, respectively (Figure 1A). In PRJNA376161 dataset, five pairs aberrant crypt foci and matching normal crypt were excluded, and 990 lncRNAs (FDR < 0.05) were identified with remaining 10 pairs primary tumor and matching normal colon tissues. Another 37 lncRNAs, with an FDR < 0.05, differently expressed among three matched pairs of CRC and normal mucosa, were isolated from PRJNA411984 dataset. After overlapping, a set of 43 significantly differentially expressed lncRNAs were identified. Among these lncRNAs, our bioinformatic approach found that 15 lncRNAs were metastasis-associated lncRNAs that were increasingly upregulated from normal colon tissues to primary tumor mucosa to metastasis samples in PRJNA218851 dataset. After a literature review, four lncRNAs were selected including ENSG00000224879, ENSG00000237721, ENSG00000254814, and ENSG00000272502.

The four lncRNA expression levels were examined in a panel of normal colon epithelial cells and 8 CRC cell lines. RNA expression of ENSG00000237721, ENSG00000254814, and ENSG00000272502 was higher in three or more CRC cell lines than in normal colon epithelial cells (Figures 1B–D); nevertheless, expression of ENSG00000224879 was particularly low in CRC cell lines compared with normal colon epithelial cells (Supplementary Figure S1A). Thus, our bioinformatic approach identified differentially expressed lncRNAs, including ENSG00000237721, ENSG00000254814, and ENSG00000272502, that were associated with the metastasis of CRC.

### Role of CCMAlnc in Metastasis-Associated CRC

**FIGURE 2:**
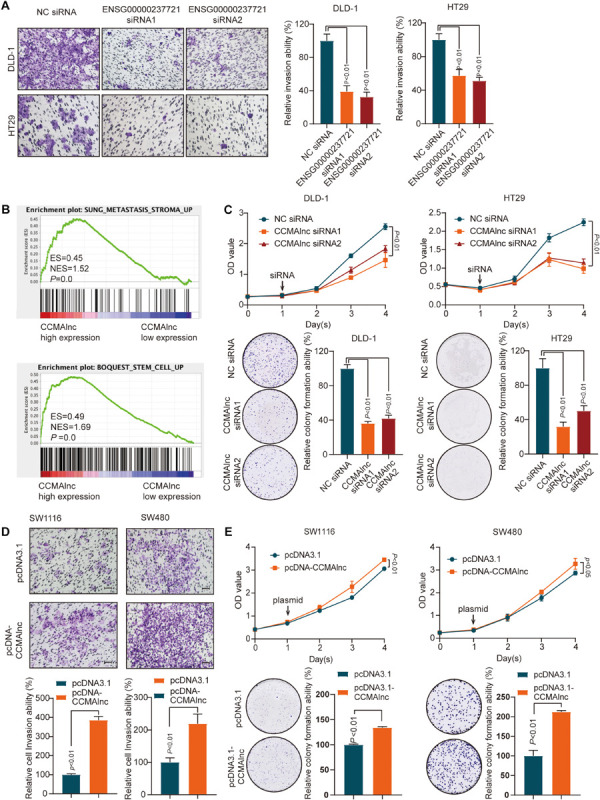
CCMAlnc promotes the proliferation and invasion of CRC cells. **(A)** A Matrigel invasion assay was performed in CRC cells after transfection of control siRNA and CCMAlnc siRNA. The invading cell numbers on each filter were counted. Data were plotted by defining the percentage of control siRNA-transfected cells as 100%. *N* = 3. Scale bars, 50 μm. **(B)** GSEA was performed to compare the CCMAlnc high-expression group (red) against the CCMAlnc low-expression group (blue) of CRC patients from the TCGA database. **(C)** CCMAlnc siRNA significantly reduced cell proliferation and anchorage-dependent growth, as detected by CCK8 proliferation and colony formation assays in DLD-1 and HT29 cells. **(D)** CCMAlnc increased cell proliferation and anchorage-dependent growth, as detected by CCK8 proliferation and colony formation assays in SW1116 and SW480 cells. **(E)** Overexpression of CCMAlnc promoted invasion in SW1116 and SW480 cells. The invading cell numbers on each filter were counted. Data were plotted by defining the percentage of pcDNA3.1-transfected cells as 100%. *N* = 3. Scale bars, 50 μm.

To evaluate the biological significance of the three lncRNAs in the progression of CRC, loss of function studies were performed. Successful knockdown of ENSG00000237721 and ENSG00000272502 was confirmed by qRT-PCR, while siRNA targeting ENSG00000254814 was not successfully designed (Supplementary Figures S2A–C). Transwell assays demonstrated that knockdown of ENSG00000237721 markedly reduced cell invasion compared with that in control cells (Figure 2A), but the invasion ability only slightly reduced with knockdown of ENSG00000272502 (Supplementary Figure S2D). We further used another siRNA and another CRC cell line to verify the invasion ability of ENSG00000237721 and confirmed our conclusion (Figure 2A and Supplementary Figure S2E). Therefore, we focused our research on ENSG00000237721 and henceforth named this lncRNA candidate colorectal cancer metastasis-associated lncRNA (CCMAlnc).

GSEA enrichment plots showed that the gene signatures of cancer metastasis and stem cell pathways were enriched in CRC cells with high expression of CCMAlnc (Figure 2B). The results of CCK8 proliferation and colony formation assays showed that knockdown of CCMAlnc significantly reduced the proliferative capacity of CRC cells and that cells transfected with siRNA of CCMAlnc formed fewer colonies than control cells transfected with NC siRNA (Figure 2C). In addition, overexpression of CCMAlnc presented the opposite results (Figures 2D,E). The effect of overexpression vector was verified by qPCR (Supplementary Figures S2F–G). These data suggested that CCMAlnc might control the cancer malignance pathway including cancer metastasis and proliferation and could be a cancer-promoting gene in CRC.

### CCMAlnc Is a Target of miR-5001-5p

**FIGURE 3:**
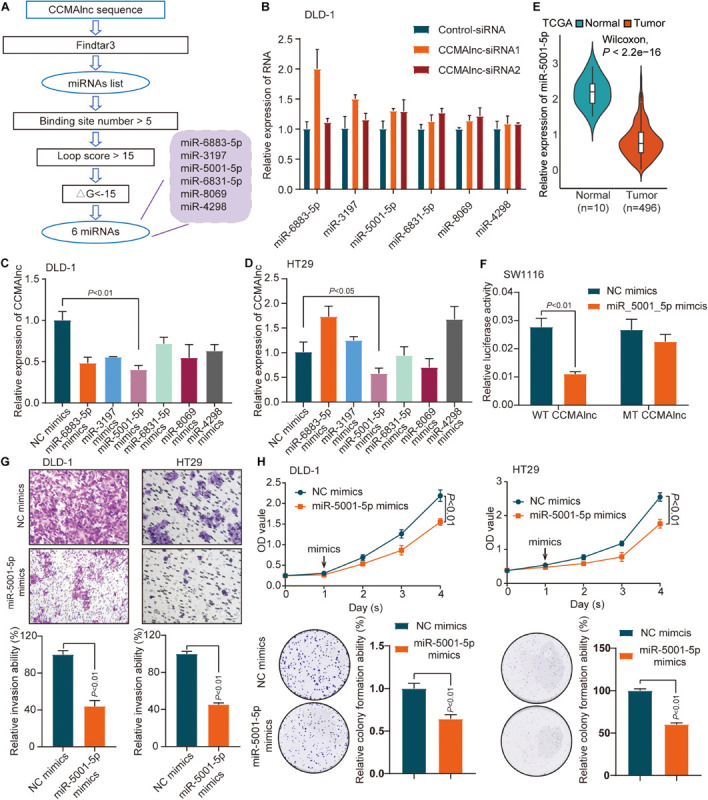
Identification of miR-5001-5p as a target of CCMAlnc. **(A)** Flowchart of CCMAlnc targeted miRNA screening. **(B)** The expression of candidate microRNAs after transfection with CCMAlnc siRNA 1 and siRNA 2. **(C,D)** The expression of CCMAlnc when transfected with candidate microRNA mimics in DLD-1 **(C)** and HT29 **(D)** cells. **(E)** The expression of miR-5001-5p in normal and CRC tissues in the TCGA database. **(F)** Luciferase reporters containing CCMAlnc or CCMAlnc-mutant were cotransfected into SW1116 cells with miR-5001-5p mimics. Luciferase activities were detected after 48 h. **(G)** Invasion of DLD-1 and HT29 cells was reduced by miR-5001-5p. The invading cell numbers on each filter were counted. Data were plotted by defining the percentage of control mimics-transfected cells as 100%. *N* = 3. Scale bars, 50 mm. **(H)** miR-5001-5p reduced cell proliferation and anchorage-dependent growth, as detected by CCK8 proliferation and colony formation assays in DLD-1 and HT29 cells.

Next, we explored the potential mechanism by which CCMAlnc promoted CRC progression. Previous studies illustrate that lncRNAs may function as miRNA sponges to compete and degrade their target miRNAs at the posttranscriptional level (Salmena et al., 2011; Thomson and Dinger, 2016). Meanwhile the target miRNA can down-regulate the expression of lncRNA. CCMAlnc was hypothesized to have a similar effect in the malignance of CRC. To determine which microRNAs interact with CCMAlnc, the full-length sequence of CCMAlnc was input into the Findtar3 database^3^, and the presence of 557 putative miRNA sites was revealed; these are listed in Supplementary Table S2. Criteria of binding sites ≥ 5, loop score > 15, ΔG < −15 were used to filter the miRNAs, and then six candidate miRNAs were selected (Figure 3A). The 6 miRNAs were all slightly increased when transfected with CCMAlnc siRNA1 or siRNA2 (Figure 3B). Interestingly, CCMAlnc was most significantly decreased with overexpression of miR-5001-5p in two CRC cell lines (Figures 3C,D). The efficiency of six mimics was validated by RT-qPCR (Supplementary Figures S3A,B). In addition, the expression of miR-5001-5p in CRC tissues was much lower in tumor tissues in TCGA database (Figure 3E). And the expression of CCMAlnc and miR-5001-5p tended to have a negative correlation in CRC cell (Supplementary Figure S3C). Thus, CCMAlnc was hypothesized to act as a miRNA sponge to modulate miR-5001-5p stability.

The direct binding of CCMAlnc and miR-5001-5p was verified by luciferase assays. miR-5001-5p mimics significantly reduced the relative luciferase activity of GV272-CCMAlnc (WT CCMAlnc) reporter vector (Figure 3F). When transfected with mutant reporter vector lacking the putative miR-5001-5p recognition sequence (MUT CCMAlnc), the changes in luciferase activity were not observed (Figure 3F). The role of miR-5001-5p in the progression of CRC was further investigated using miR-5001-5p mimics. Using the Matrigel invasion assay, a significant decrease in cell invasion ability was observed with overexpression of miR-5001-5p (Figure 3G). The proliferative capacity of cells was significantly suppressed when transfected with miR-5001-5p mimics, and fewer colonies were formed in these cells than in control cells, as verified in two CRC cell lines (Figure 3H). Thus, CCMAlnc was hypothesized to promote the malignance of CRC through repressing the expression of miR-5001-5p.

### CCMAlnc Controls miR-5001-5p Target Expression

**FIGURE 4:**
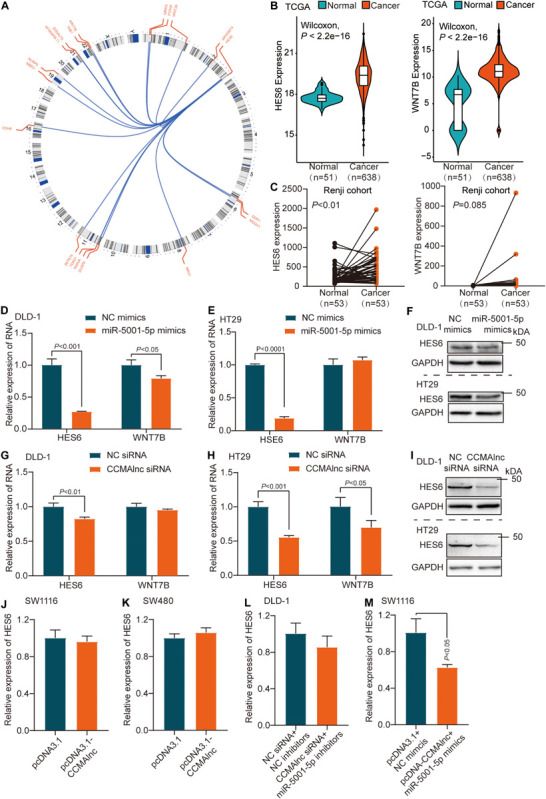
HES6 is a target of miR-5001-5p controlled by CCMAlnc. **(A)** Circos plot of predicted target genes of miR-5001-5p reported to participate in the growth and metastasis of CRC. **(B,C)** The expression of HES6 and WNT7B in normal and CRC tissues in the TCGA database **(B)** and Renji cohort **(C)**. **(D,E)** The expression of HES6 and WNT7B after transfection with miR-5001-5p mimics and control mimics in DLD-1 and HT29 cells at RNA level. **(F)** The expression of HES6 after transfection with miR-5001-5p mimics and control mimics in DLD-1 and HT29 cells at protein level. **(G,H)** The expression of HES6 and WNT7B after transfection with CCMAlnc siRNA and control siRNA in DLD-1 and HT29 cells at RNA level. **(I)** The expression of HES6 after transfection with CCMAlnc siRNA and control siRNA in DLD-1 and HT29 cells at protein level. **(J,K)** The expression of HES6 in SW1116 and SW480 cells transfected with CCMAlnc overexpression vector and control vector. **(L)** The effect of control siRNA or CCMAlnc siRNA combined with miR-5001-5p inhibitiors on the RNA level of HES6. **(M)** The effect of control vector or CCMAlnc overexpression vector combined with miR-5001-5p overexpression vector on the RNA level of HES6.

We used FindTar3^4^ and Miranda microRNA^5^ software to predict the target genes of miR-5001-5p, and a set of 449 genes was discovered (Supplementary Table S3). Among the many predicted targets of miR-5001-5p, we concentrated on 19 genes since they were reported to participate in the growth and metastasis of colorectal carcinoma (Figure 4A). In the TCGA database, only HES6 and WNT7B were expressed at higher levels in CRC samples than in normal samples, consistent with CCMAlnc expression (Figure 4B). We further verified expression of the two genes in Renji hospital samples and the expression of HES6 in cancer and para-cancer was consistent with the TCGA database (Figure 4C). Moreover, overexpression of miR-5001-5p decreased HES6 expression compared to NC mimics treated cells (Figures 4D–F). However, miR-5001-5p mimics did not significantly reduce the expression of WNT7B in CRC cells (Figures 4D,E). To explore whether HES6 and WNT7B were regulated by CCMAlnc through competing with miR-5001-5p, overexpression and knockdown experiments were performed. CCMAlnc depletion remarkably reduced the expression of HES6 at both the RNA and protein levels in the two CRC cell lines (Figures 4G–I). Consistently, the expression of WNT7B was not remarkably reduced in two CRC cells when transfected with CCMAlnc siRNA (Figures 4G,H). In addition, the expression of HES6 did not change when cells were transfected with the CCMAlnc overexpression vector compared with the control vector (Figures 4J,K). Therefore, we focused our attention on HES6 and hypothesized that CCMAlnc functioned to stabilize the expression of HES6, rather than increase the expression of HES6.

Given that both repressing CCMAlnc and overexpressing miR-5001-5p can decrease the expression of HES6, we next investigated whether the suppressive effect of CCMAlnc on HES6 was dependent on miR-5001-5p. A rescue experiment was conducted in which we cotransfected DLD-1 cells with both CCMAlnc siRNA and miR-5001-5p inhibitors, and the effect on HES6 expression was evaluated. MiR-5001-5p depletion reduced the increase of HES6 caused by knockdown of CCMAlnc, and the level of HES6 remained unchanged (Figure 4L). In addition, overexpression of both CCMAlnc and miR-5001-5p decreased the expression of HES6 (Figure 4M). These findings suggested that CCMAlnc stabilized the expression of target HES6 by competing with miR-5001-5p.

### MiR-5001-5p Targets the 3′-UTR of HES6 mRNA

**FIGURE 5:**
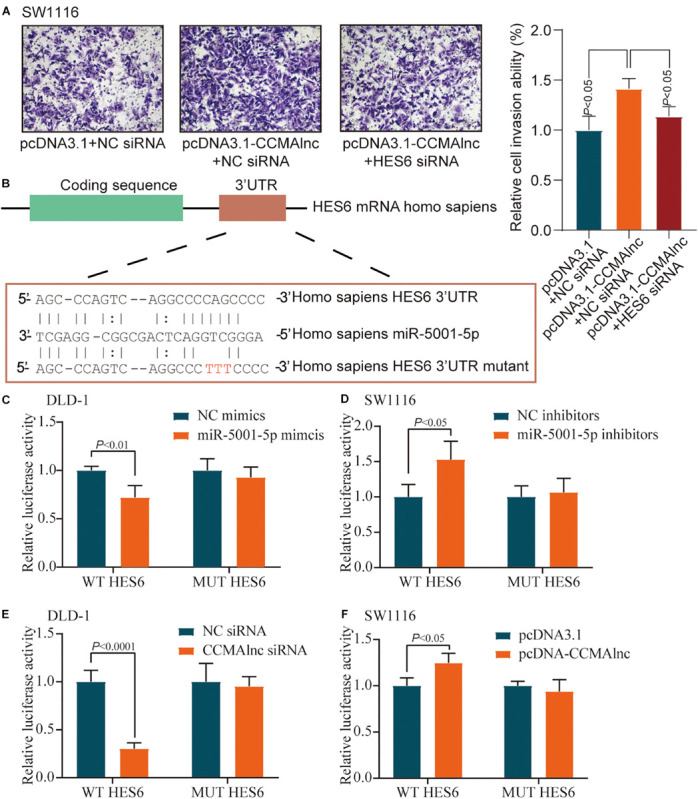
miR-5001-5p targets the 3′-UTR of HES6 mRNA. **(A)** A Matrigel invasion assay was performed in SW1116 cells after transfection of control vector or CCMAlnc overexpression vector combined with HES6 siRNA. The invading cell numbers on each filter were counted. Data were plotted by defining the percentage of control cells as 100%. *N* = 3. Scale bars, 50 μm. **(B)** One of the predicted binding sites of miR-5001-5p with the 3′-UTR of HES6 mRNA. **(C)** Luciferase reporters containing HES6-3′-UTR or HES6-3′-UTR-mutant were cotransfected into DLD-1 cells with miR-5001-5p mimics or miR-5001-5p inhibitors. Luciferase activities were detected after 48 h. **(D)** Luciferase reporters containing HES6-3′-UTR or HES6-3′-UTR-mutant were cotransfected into SW1116 cells with miR-5001-5p inhibitors. Luciferase activities were detected after 48 h. **(E)** Luciferase reporters containing HES6-3′-UTR or HES6-3′-UTR-mutant were cotransfected into DLD-1 cells with CCMAlnc siRNA. Luciferase activities were detected after 48 h. **(F)** Luciferase reporters containing HES6-3′-UTR or HES6-3′-UTR-mutant were cotransfected into SW1116 cells with CCMAlnc overexpression vector. Luciferase activities were detected after 48 h.

The result of transwell assays showed that upregulation of CCMAlnc could promote cell invasion, while simultaneously knockdown of HES6 could inhabit the invasion ability caused by CCMAlnc (Figure 5A). The efficiency of HES6 siRNA was validated by RT-qPCR (Supplementary Figure S4A). Considering the vital role of HES6 in the progression of CRC, the exact mechanism underlying the effect of miR-5001-5p on the expression of HES6 was further investigated. miRNAs induce posttranscriptional regulation mainly by binding to the 3′-UTR region of the target gene. The sequence alignments for miR-5001-5p and the 3′-UTR of HES6 mRNA are shown in Figure 5B; they suggest that HES6 may be one of the potential targets of miR-5001-5p.

To confirm the direct binding between miR-5001-5p and HES6, pmirGLO-HES6-3′UTR construct (WT HES6) or mutant derivatives lacking the putative miR-5001-5p recognition sequence (MUT HES6) were transfected into CRC cell lines together with either miR-5001-5p mimics or miR-5001-5p inhibitors. Luciferase activity of WT HES6 reporter vector was reduced when transfected with miR-5001-5p mimics (Figure 5C). Conversely, miR-5001-5p inhibitors significantly increased the relative luciferase activity of WT HES6 reporter vector (Figure 5D). The effects were abolished when mutant substrates for either miR-5001-5p mimics or miR-5001-5p inhibitors were utilized (Figures 5C,D). These data suggested that miR-5001-5p was responsible for HES6 stability.

The effect of CCMAlnc on luciferase activity of HES6 reporter vector was further verified. CCMAlnc siRNA significantly reduced the relative luciferase activity of WT HES6 reporter vector (Figure 5E). Overexpression of CCMAlnc increased the relative luciferase activity of WT HES6 reporter vector (Figure 5F). When transfected with MUT HES6 reporter vector, all the changes in luciferase activity were not observed (Figures 5E,F). These data indicated that CCMAlnc competed with miR-5001-5p, which could bind to HES6 and reduce its expression.

### CCMAlnc Is Upregulated in CRC and Associated With Poor Prognosis

**FIGURE 6:**
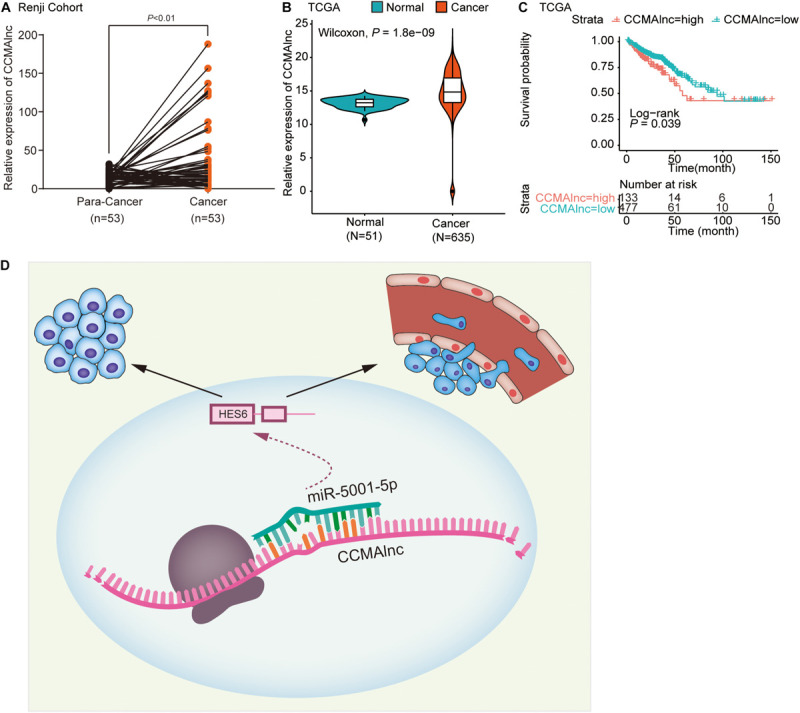
CCMAlnc is upregulated in CRC and associated with poor prognosis. **(A)** Statistical analysis of CCMAlnc expression in CRC and the paired adjacent normal tissues of the Renji cohort (*n* = 53). **(B)** The expression of CCMAlnc in CRC and normal tissues in the TCGA database. **(C)** Survival was analyzed and compared between patients with high and low CCMAlnc expression in the TCGA database. **(D)** Schematic representation of the mechanism of the CCMAlnc/miR-5001-5p axis as a switch that regulates human CRC progression by stabilizing the expression of HES6.

CCMAlnc is a non-coding RNA, which was verified by the coding potential calculator, ORF Finder of NCBI and PhyloCSF analysis (Supplementary Figures S5A–C). The existence and expected size of full length of CCAMlnc was confirmed by northern blot assay (Supplementary Figure S5D). To investigate the clinical correlation between CCMAlnc and CRC, we evaluated CCMAlnc expression in multiple groups of primary tumors and normal colon mucosa. CRC tissues and normal colorectal tissues adjacent to cancer lesions from 53 patients were collected at Renji Hospital. Upregulation of CCMAlnc in CRC tissues was validated in our local Renji cohort (*P* < 0.01) (Figure 6A). We further assessed CCMAlnc expression in 51 normal samples and 635 primary tumors in TCGA data, and the mean CCMAlnc expression was significantly higher in primary tumors than in normal samples (Figure 6B). Furthermore, the correlation between CCMAlnc, miR-5001-5p and HES6 was analyzed in TCGA database. Considering the poor prognosis of stage IV patients and their poor clinical significance of the diagnostic target, the stage IV severe patients were removed. It was found that CCMAlnc was negatively correlated with miR-5001-5p, while positively correlated with HES6 (Supplementary Figures S5E,F). To assess the impact of CCMAlnc on the outcome of patients with CRC, we analyzed the effect of CCMAlnc expression on patient survival in TCGA data. Kaplan-Meier survival analysis showed that CRC patients with high CCMAlnc expression had significantly shorter disease-free survival than those with low CCMAlnc expression (*P* = 0.039, Figure 6C). These data indicated that CCMAlnc was markedly elevated in CRC tissue compared to normal mucosa. CCMAlnc expression might affect patient outcome and represent a new prognostic factor in patients with CRC.

## Discussion

To date, cancer metastasis remains the predominant cause of CRC-related death (Nishida et al., 2014; Zhang et al., 2015). In this study, the lncRNAs that promote malignancy of CRC were investigated from the perspective of cancer metastasis. CRC metastasis is a complex process that includes a degradation of extracellular matrix, a decrease in cell adhesion, an enhancement of the migratory ability of CRC cells and a change in the tumor microenvironment (Gregorieff et al., 2005; Highfill et al., 2014; Said et al., 2014; Marei et al., 2016). Multiple gene signatures, including NLRP3, CAF, JMDJ1C, FOXC1, and FOXC2, contribute to the carcinogenesis and metastasis of CRC (Cui et al., 2015; Chen et al., 2018; Liu et al., 2018; Paauwe et al., 2018; Deng et al., 2019). These genes function by altering the activities of several intracellular signaling pathways, such as Notch, NF-κB, MAPK, and AKT signaling pathways (Agarwal et al., 2017; Wang et al., 2018; Xu J. et al., 2018; Jackstadt et al., 2019). Key regulators of Wnt signaling, β-catenin and E-cadherin were reported to be activated by various genes and to promote CRC metastasis (Wang et al., 2016; Dong et al., 2017; Zhang et al., 2018; He et al., 2019; Li et al., 2019).

lncRNA has been demonstrated to be involved in different aspects of gene regulation in diverse cellular contexts and biological processes. Through a combination of bioinformatic, genetic, functional, and clinical approaches, we demonstrated that CCMAlnc is a novel oncogenic lncRNA in CRC that promotes cancer malignance. CCMAlnc was significantly upregulated from normal tissues to CRC tissues to metastatic tissues and the relationship between CCMAlnc and CRC was verified in CRC cell lines and invasion experiment. GSEA analyses have demonstrated that metastasis, stem cell pathways were remarkably enriched in response to CCMAlnc alteration in the datasets of patients with CRC. The bioinformatic analyses have been functionally validated in several *in vitro* experiment. In cultured CRC cells, downregulated CCMAlnc markedly suppressed CRC cell metastasis and growth. These data consistently point to the notion that CCMAlnc may be a decisive factor of controlling CRC malignance.

lncRNAs can regulate genome organization, remodel chromatin and regulate transcription (Yao et al., 2019). Recent data suggest that lncRNAs can regulate target genes through their ability to compete for miRNA binding, termed ceRNA (Salmena et al., 2011). ceRNAs can sequester miRNAs, thus protecting their targets RNAs from repression (Karreth and Pandolfi, 2013; Tay et al., 2014; Thomson and Dinger, 2016).

We assumed that CCMAlnc promoted CRC malignance through the same mechanism. miR-5001-5p and its target gene HES6 were screened with FindTar3 and Miranda microRNA software. It has been reported that HES6 has a prognostic value and promotes metastasis in CRC (Swearingen et al., 2003; Xu Y. et al., 2018). In this paper, we discovered that CCMAlnc displayed decoy activity for miR-5001-5p and, in doing so, regulated its target HES6 in a molecular circuitry affecting the cancer malignance program. This hypothesis is supported by several lines of experimental evidence: (I) CCMAlnc contained five predicted binding sites for miR-5001-5p. Luciferase assay verified that miR-5001-5p could interact directly with CCMAlnc. And CCMAlnc was most significantly decreased when two CRC cell lines were transfected with miR-5001-5p mimics. (II) the expression level of miR-5001-5p in normal and CRC tissues was opposite to that of CCMAlnc. (III) Overexpression of miR-5001-5p remarkably inhibited the invasion and growth of CRC cells. (IV) HES6, a target gene of miR-5001-5p, was up expressed in CRC tissues. (V) HES6 was significantly downregulated by CCMAlnc through miR-5001-5p in several qRT-PCR and Western blot experiments. (VI) Luciferase assays showed that miR-5001-5p could physically interact with HES6. And CCMAlnc was validated to act as a molecular sponge to determine the availability of miR-5001-5p, which then supports the expression of HES6.

The target gene HES6 was reported to play a vital role in different kind of cancer. HES6 is highly expressed in metastatic prostate adenocarcinomas and required for the hypoxia-mediated neuroendocrine phenotype, the metastasis of prostate adenocarcinomas, and the formation of neuroendocrine tumors (Qi et al., 2010). HES6 also drives a critical AR transcriptional program to induce castration-resistant prostate cancer through the activation of an E2F1-mediated cell cycle network (Ramos-Montoya et al., 2014). Moreover, HES6 contributed to glioma cell proliferation and migration and had a role in angiogenesis (Haapa-Paananen et al., 2012). The association between CRC and HES6 was first reported by Swearingen et al. (2003), and the molecular mechanism was further verified. HES6 may be a novel prognostic marker that promotes metastasis via the Wnt/β-catenin signaling pathway in CRC (Xu Y. et al., 2018). Considering the close connection between HES6 and Wnt/β-catenin in CRC, we made a bold assumption that CCMAlnc promoted CRC metastasis through the Wnt/β-catenin signaling pathway. The detailed mechanism requires further exploration in the future.

In addition to the biological importance of CCMAlnc, our work may be relevant to its clinical significance. CCMAlnc was upregulated in CRC patients from TCGA and Renji datasets. Survival analyses illustrated that CCMAlnc overexpression could predict poor clinical outcome in patients with CRC from TCGA datasets, which indicated that patients with high CCMAlnc expression should be treated early and followed up closely after surgery.

In summary, our integrated and comprehensive study identified a novel mechanism by which CCMAlnc modulated HES6 via sequestering miR-5001-5p and further promoted CRC malignance (Figure 6D). The interaction between CCMAlnc, miR-5001-5p and HES6 provides a potential target for early diagnosis. Targeting this network may be therapeutically meaningful in treating CRC patients.

## Data Availability Statement

The original contributions presented in the study are included in the article/Supplementary Material, further inquiries can be directed to the corresponding author/s.

## Ethics Statement

The studies involving human participants were reviewed and approved by the Ethics Committee of Renji Hospital. The patients/participants provided their written informed consent to participate in this study.

## Author Contributions

YY, BX, ZG, CS, and YC performed the experiments and analyzed the data. ZC, GY, and J-YF provided the clinical specimen. HC conducted the data analysis. JH and ZW conceived and wrote the manuscript, and supervised the study. All authors contributed to the article and approved the submitted version.

## Conflict of Interest

The authors declare that the research was conducted in the absence of any commercial or financial relationships that could be construed as a potential conflict of interest.

## Footnotes

1 http://cran.r-project.org/bin/windows/base/old/3.0.2/

2 https://www.ncbi.nlm.nih.gov/sra/

3 http://bio.sz.tsinghua.edu.cn

4 http://www.tsinghua.edu.cn/publish/newthu/index.html

5 www.microrna.org
