# Supplementary figures and images for: CCMAlnc Promotes the Malignance of Colorectal Cancer by Modulating the Interaction Between miR-5001-5p and Its Target mRNA
Source: Front Cell Dev Biol. 2020 Dec 16;8:566932. doi: 10.3389/fcell.2020.566932 (PMC7931267; doi:10.3389/fcell.2020.566932)

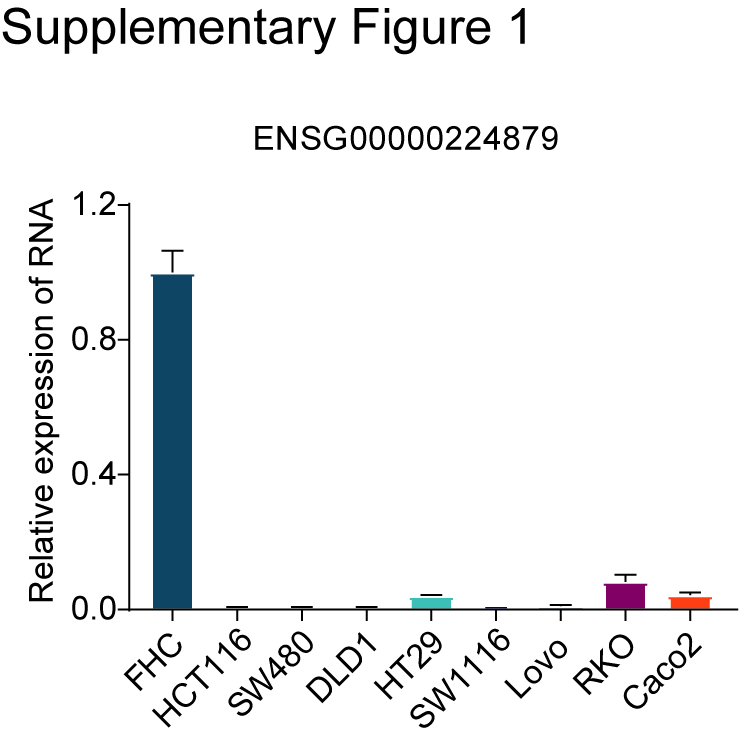

Supplement: Supplementary file 1 [file Image_1.TIF]

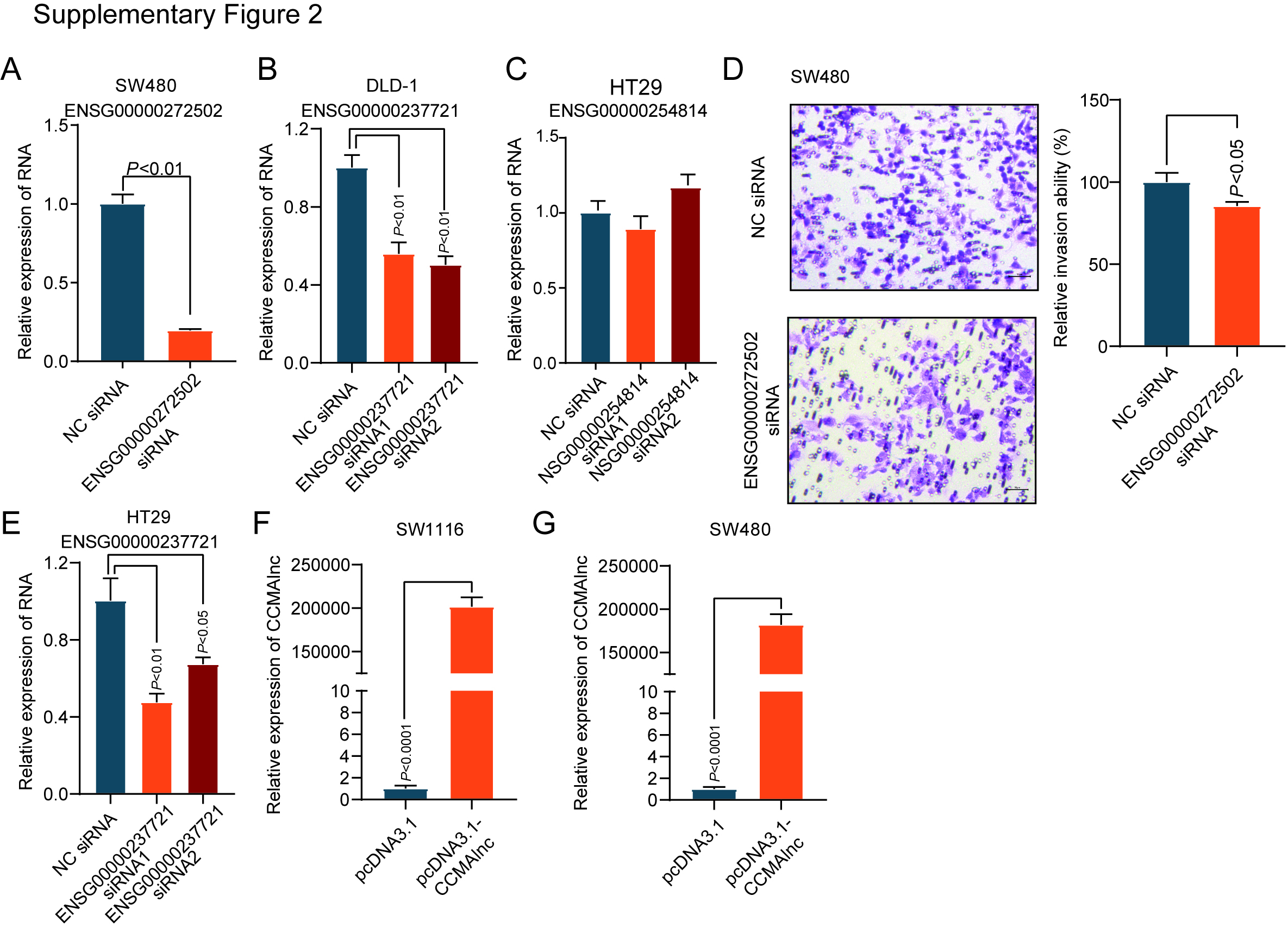

Supplement: Supplementary file 2 [file Image_2.jpg]

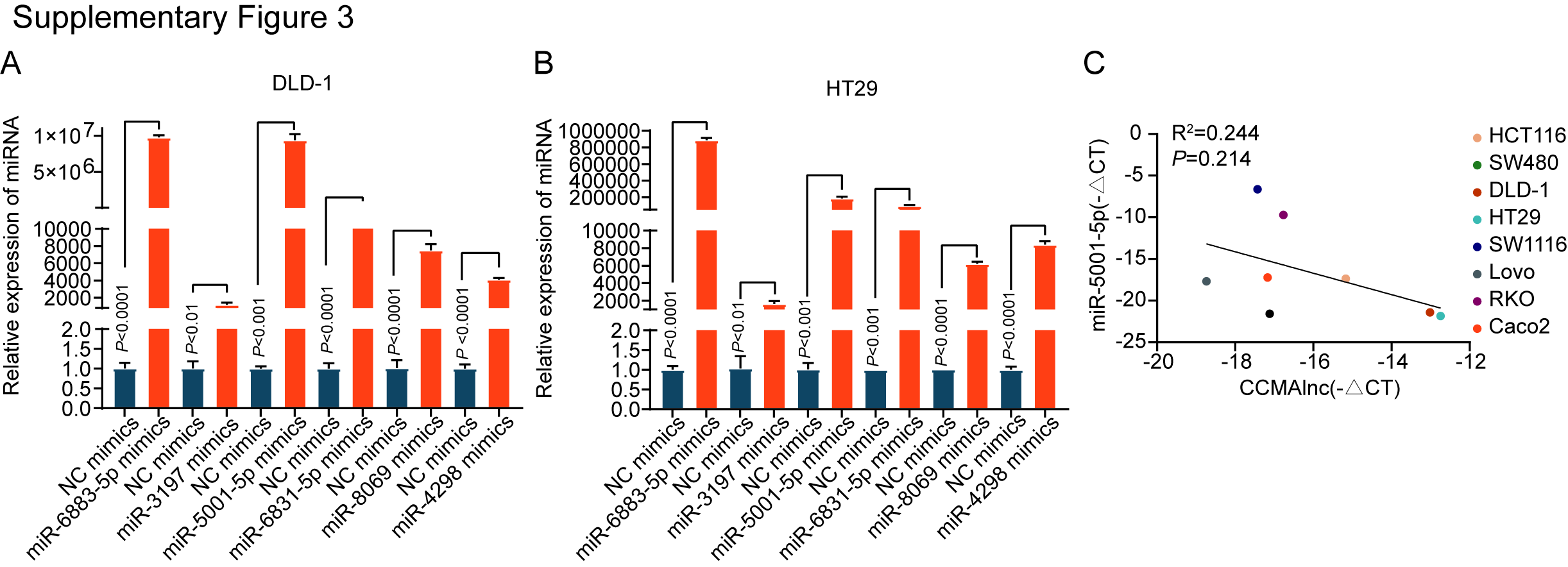

Supplement: Supplementary file 3 [file Image_3.TIF]

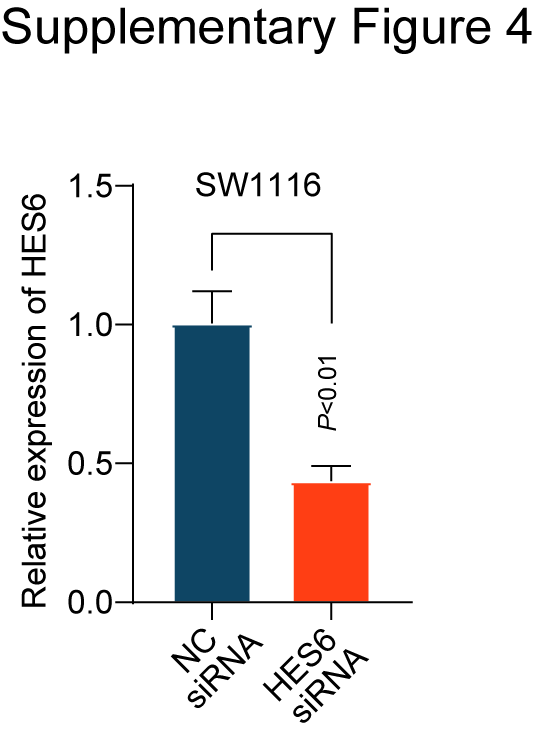

Supplement: Supplementary file 4 [file Image_4.TIF]

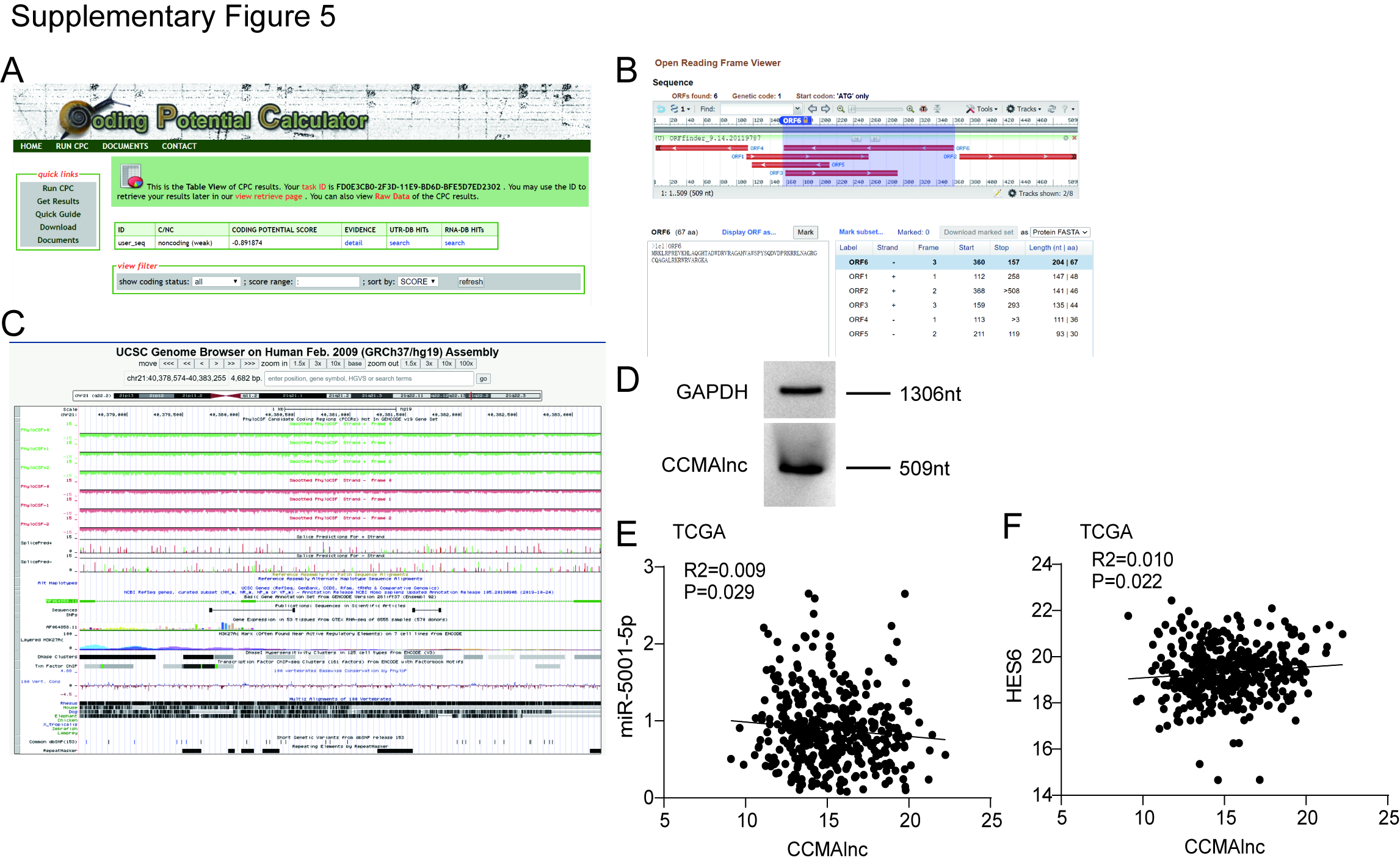

Supplement: Supplementary file 5 [file Image_5.TIF]
