# Supplementary material for: CCMAlnc Promotes the Malignance of Colorectal Cancer by Modulating the Interaction Between miR-5001-5p and Its Target mRNA
Source: Front Cell Dev Biol. 2020 Dec 16;8:566932. doi: 10.3389/fcell.2020.566932 (PMC7931267; doi:10.3389/fcell.2020.566932)
Supplement: Supplementary file 6 [file Table_1.PDF]

Table S1 The clinical characteristics of CRC patients

Samples ID	Gender	Age/year	Disease Type	Anatomic neoplasm subdivision	Tumor size/cm	TNM staging	Tumor stage	Lymphatic metastasis	Vascular invasion	Liver metastasis	Other organs metastasis	Intraperitoneal implantatio
1	Male	66	Colon Adenocarcinoma	Descending Colon	6×4×3	T3N0M1a	stage IV	-	+	+	-	-
2	Male	70	Colon Adenocarcinoma	Sigmoid Colon	4×3×1	T4N2M1	stage IV	+	-	-	-	-
3	Male	69	Colon Adenocarcinoma	Sigmoid Colon	4×2.5×1	T4N2M0	stage III	+	-	-	-	-
4	Male	31	Colon Adenocarcinoma	Sigmoid Colon	3×3×1	T4N0M0	stage II	-	-	-	-	-
5	Female	65	Colon Adenocarcinoma	Sigmoid Colon	2.5×2×0.8	T2N0M0	stage I	-	-	-	-	-
6	Female	51	Colon Adenocarcinoma	Rectum	1.5×1×1	T3N0M0	stage IIA	-	-	-	-	-
7	Male	60	Colon Adenocarcinoma	Hepatic Flexure	4×3×1	T1N0M0	stage I	-	-	-	-	-
8	Male	65	Colon Adenocarcinoma	Rectum	5.5×4×1	T4N1M1	stage Ⅳ	+	-	+	-	-
9	Male	71	Colon Adenocarcinoma	Sigmoid Colon	5×4×2	T2N0M0	stage I	-	-	-	-	-
10	Female	67	Colon Adenocarcinoma	Sigmoid Colon	3×1.8×0.5	T1N1M0	stage III	+	-	-	-	-
11	Male	82	Colon Adenocarcinoma	Hepatic Flexure	5×4.5×2	T1N0M0	stage I	-	-	-	-	-
12	Male	56	Colon Adenocarcinoma	Descending Colon	4×4×2	T4N0M0	stage II	-	-	-	-	-
13	Male	53	Colon Adenocarcinoma	Ascending Colon	4.5×3×1	T4N1M1	stage IV	+	-	-	-	+
14	Female	51	Colon Adenocarcinoma	Junction of the rectosigmoid colon	3.5×3.5×1	T4aN0M0	stage IIB	-	-	-	-	-
15	Female	75	Colon Adenocarcinoma	Sigmoid Colon	3×3×1.5	T3N0M0	stage IIa	-	-	-	-	-
16	Female	67	Colon Adenocarcinoma	Ascending Colon	3.5×3×1	T4N0M0	stage II	-	-	-	-	-
17	Male	82	Colon Adenocarcinoma	Ascending Colon	3.5×2.5×1	T3N1M0	stage IIIB	+	-	-	-	-
18	Male	59	Colon Adenocarcinoma	Sigmoid Colon	2.5×2×0.5	T4N0M0	stage II	-	-	-	-	-
19	Male	63	Colon Adenocarcinoma	Rectum	3×2×0.6	T2N0M0	stage I	-	-	-	-	-
20	Male	59	Colon Adenocarcinoma	Sigmoid Colon	3×3×0.8	T1N1M0	stage IIIa	+	-	-	-	-
21	Male	59	Colon Adenocarcinoma	Rectum	4×3×0.5	T2N0M0	stage I	-	-	-	-	-
22	Male	54	Colon Adenocarcinoma	Sigmoid Colon	7.5×5×2	T3N0M0	stage IIA	-	-	-	-	-
23	Male	46	Colon Adenocarcinoma	Ascending Colon	6×4×1.5	T1N0M0	stage I	-	-	-	-	-
24	Female	53	Colon Adenocarcinoma	Rectum	3.5×1×0.5	T4N0M0	stage II	-	-	-	-	-
25	Male	77	Colon Adenocarcinoma	Rectum	5×4×0.8	T4N0M0	stage II	-	-	-	-	-
26	Male	71	Colon Adenocarcinoma	Cecum	5× 4.5×	T3N1M0	stage ⅢA	+	-	-	-	-
27	Female	70	Colon Adenocarcinoma	Sigmoid Colon	4×3.5×0.8	T3N1M0	stage III	+	-	-	-	-
28	Male	62	Colon Adenocarcinoma	Descending Colon	4×3×1	T1N0M0	stage I	-	-	-	-	-
29	Male	49	Colon Adenocarcinoma	Sigmoid Colon	3×3×1	T4N2M0	stage III	+	-	-	-	-
30	Male	58	Colon Adenocarcinoma	Rectum	7.5×5×3	T4N3M0	stage III	+	-	-	-	-
31	Female	63	Colon Adenocarcinoma	Ascending Colon	4.5×4.5×2	T4N1M1	stage IV	+	-	-	lung metastasis	-
32	Male	88	Colon Adenocarcinoma	Descending Colon	3×3×0.8	T3N0M0	stage IIA	-	-	-	-	-
33	Female	60	Colon Adenocarcinoma	Ascending Colon	3×3×1	T4N3M0	stage III	+	-	-	-	-
34	Male	87	Colon Adenocarcinoma	Ascending Colon	4×3×1	T4N2M0	stage III	+	-	-	-	-
35	Male	73	Colon Adenocarcinoma	Rectum	4×3×0.5	T3N0M0	stage IIA	-	-	-	-	-
36	Male	62	Colon Adenocarcinoma	Sigmoid Colon	4×3×1	T1N0M1	stage Ⅳ	-	-	+	-	-
37	Male	59	Colon Adenocarcinoma	Rectum	6.5×5.5×1	T4aN2bM0	stage IIIc	+	+	-	-	-
38	Male	79	Colon Adenocarcinoma	Sigmoid Colon	4×4×1	T4N1M0	stage III	+	-	-	-	-
39	Female	69	Colon Adenocarcinoma	Sigmoid Colon	6×4×1.5	T1N0M0	stage I	-	-	-	-	-
40	Male	64	Colon Adenocarcinoma	Transverse Colon	4×4×1	T4N2M0	stage III	+	-	-	-	-
41	Female	75	Colon Adenocarcinoma	Ascending Colon	5×3×1	T4N0M1	stage IV	-	-	+	-	-
42	Male	65	Colon Adenocarcinoma	Rectum	5×4×1	T4N0M0	stage II	-	-	-	-	-
43	Female	75	Colon Adenocarcinoma	Rectum	4×2.5×1	T1N1M1	stage Ⅳ	+	-	-	lung metastasis	-
44	Male	73	Colon Adenocarcinoma	Rectum	5×3×0.5	T4N0M0	stage II	-	-	-	-	-
45	Male	66	Colon Adenocarcinoma	Rectum	4×3×1	T4N2M0	stage III	+	-	-	-	-
46	Male	31	Colon Adenocarcinoma	Rectum	5×3×2	T4bN2M1	stage IV	+	-	-	-	-
47	Female	56	Colon Adenocarcinoma	Ascending Colon	6×5×4	T3N0M0	stage IIA	-	-	-	-	-
48	Male	29	Colon Adenocarcinoma	Rectum	3×2×1	T4N2M1	stage IV	+	-	+	-	+
49	Male	64	Colon Adenocarcinoma	Sigmoid Colon	2.5×2×1	T4N2M0	stage IIIB	+	-	-	-	-
50	Male	69	Colon Adenocarcinoma	Rectum	7×5×1.5	T3N0M0	stage IIA	-	-	-	-	-
51	Male	42	Colon Adenocarcinoma	Rectum	5×3.5×0.5	T4N0M0	stage IIC	-	-	-	-	-
52	Male	68	Colon Adenocarcinoma	Sigmoid Colon	5×5×1.2	T4N1M0	stage III	+	-	-	-	-
53	Male	50	Colon Adenocarcinoma	Transverse Colon	14×9×2.5	T1N0M0	stage I	-	-	-	-	-
