# Supplementary material for: CCMAlnc Promotes the Malignance of Colorectal Cancer by Modulating the Interaction Between miR-5001-5p and Its Target mRNA
Source: Front Cell Dev Biol. 2020 Dec 16;8:566932. doi: 10.3389/fcell.2020.566932 (PMC7931267; doi:10.3389/fcell.2020.566932)
Supplement: Supplementary file 7 [file Table_2.PDF]

Table S2 CCMAlnc target microRNAs

miRNA		folding energy		
hsa_let_7b_5p	174	-17	CCACACGCCACCTCTTCCTCA	TGAGGTAGTAGGTTGTGTGGTT
hsa_let_7e_5p	174	-17.5	CCACACGCCACCTCTTCCTCA	TGAGGTAGGAGGTTGTATAGTT
hsa_miR_103a_2_5p	469	-17.3	ACAGAAAGGGCTGGAAGAAGCA	AGCTTCTTTACAGTGCTGCCTTG
hsa_miR_105_3p	279	-15.4	GAGCTCCAGCTCTAACTCTGT	ACGGATGTTTGAGCATGTGCTA
hsa_miR_1178_5p	278	-15.6	TGAGCTCCAGCTCTAACTCTG	CAGGGTCAGCTGAGCATG
hsa_miR_1182	191	-19.1	CTCAGTGCCCCAGCCTGGCATCCTC	GAGGGTCTTGGGAGGGATGTGAC
hsa_miR_1182	146	-18.5	AGGACAGCCCCTCATGCCTTC	GAGGGTCTTGGGAGGGATGTGAC
hsa_miR_1183	329	-18.3	TGCTTAACTTCTCTGGGCCTCAGTT	ACTGTAGGTGATGGTGAGAGTGGGC
hsa_miR_1193	34	-18.8	TGACTTCCTGGTGCCATCCT	GGGATGGTAGACCGGTGACGTGC
hsa_miR_1193	147	-16.1	GGACAGCCCCTCATGCCTTCCC	GGGATGGTAGACCGGTGACGTGC
hsa_miR_1200	475	-18.6	AGGGCTGGAAGAAGCAGGGG	CTCCTGAGCCATTCTGAGCCTC
hsa_miR_1202	195	-19.6	GTGCCCCAGCCTGGCAT	GTGCCAGCTGCAGTGGGGGAG
hsa_miR_1207_5p	185	-25.1	CTCTTCCTCAGTGCCCCAGCCT	TGGCAGGGAGGCTGGGAGGGG
hsa_miR_1207_5p	35	-21.3	GACTTCCTGGTGCCATCCTTGGCT	TGGCAGGGAGGCTGGGAGGGG
hsa_miR_1207_5p	278	-17.9	TGAGCTCCAGCTCTAACTCTGTCC	TGGCAGGGAGGCTGGGAGGGG
hsa_miR_1224_5p	333	-18.3	TAACTTCTCTGGGCCTCAG	GTGAGGACTCGGGAGGTGG
hsa_miR_1226_5p	336	-24.6	CTTCTCTGGGCCTCAGTTTTCTCAT	GTGAGGGCATGCAGGCCTGGATGGGG
hsa_miR_1227_5p	47	-25.1	CCATCCTTGGCTCCAT	GTGGGGCCAGGCGGTGG
hsa_miR_1227_5p	155	-16.4	CCTCATGCCTTCCCGC	GTGGGGCCAGGCGGTGG
hsa_miR_1227_5p	129	-15.3	CTACTTCCTGTTCCAG	GTGGGGCCAGGCGGTGG
hsa_miR_1229_5p	278	-22.3	TGAGCTCCAGCTCTAACTCTGTCCCAA	GTGGGTAGGGTTTGGGGGAGAGCG
hsa_miR_1233_5p	339	-15.2	CTCTGGGCCTCAGTTTTCTCATC	AGTGGGAGGCCAGGGCACGGCA
hsa_miR_1237_3p	474	-17.9	AAGGGCTGGAAGAAGCAGGGGA	TCCTTCTGCTCCGTCCCCCAG
hsa_miR_1237_5p	14	-21.2	AGCAGCAAACACTGCCCCCG	CGGGGGCGGGGCCGAAGCGCG
hsa_miR_1249_5p	148	-19.5	GACAGCCCCTCATGCCTTCCC	AGGAGGGAGGAGATGGGCCAAGTT
hsa_miR_125a_3p	344	-22.6	GGCCTCAGTTTTCTCATCTGT	ACAGGTGAGGTTCTTGGGAGCC
hsa_miR_125b_1_3p	186	-18.6	TCTTCCTCAGTGCCCCAGCCTGG	ACGGGTTAGGCTCTTGGGAGCT
hsa_miR_1268b	111	-15.8	CATGCCCTTCAGAATGTTCT	CGGGCGTGGTGGTGGGGGTG
hsa_miR_127_5p	330	-18.6	GCTTAACTTCTCTGGGCCTCAG	CTGAAGCTCAGAGGGCTCTGAT
hsa_miR_1270	281	-17	GCTCCAGCTCTAACTCTGTCCCAA	CTGGAGATATGGAAGAGCTGTGT
hsa_miR_1273f	222	-18.5	CATTCAGCCTTCTTTTCC	GGAGATGGAGGTTGCAGTG
hsa_miR_1273g_3p	473	-20.9	AAAGGGCTGGAAGAAGCAGGGGA	ACCACTGCACTCCAGCCTGAG
hsa_miR_1273g_5p	214	-15.6	TCTTCCTGCATTCAGCCTTC	GGTGGTTGAGGCTGCAGTAAGT
hsa_miR_1275	207	-19.7	GGCATCCTCTTCCTGCAT	GTGGGGGAGAGGCTGTC
hsa_miR_1275	179	-17.9	CGCCACCTCTTCCTCAG	GTGGGGGAGAGGCTGTC
hsa_miR_128_2_5p	40	-17.2	CCTGGTGCCATCCTTGGCTCCA	GGGGGCCGATACACTGTACGAGA
hsa_miR_1282	476	-15	GGGCTGGAAGAAGCAGGGGA	TCGTTTGCCTTTTTCTGCTT
hsa_miR_1289	41	-19.2	CTGGTGCCATCCTTGGCTCCA	TGGAGTCCAGGAATCTGCATTTT
hsa_miR_1291	327	-17.7	GATGCTTAACTTCTCTGGGCCT	TGGCCCTGACTGAAGACCAGCAGT
hsa_miR_1294	187	-18.5	CTTCCTCAGTGCCCCAGCCTGGCA	TGTGAGGTTGGCATTGTTGTCT
hsa_miR_1303	274	-16.2	AACGTGAGCTCCAGCTCTAAC	TTTAGAGACGGGGTCTTGCTCT
hsa_miR_130a_3p	112	-16.4	ATGCCCTTCAGAATGTTCTACTT	CAGTGCAATGTTAAAAGGGCAT
hsa_miR_1321	218	-15.3	CCTGCATTCAGCCTTCTTT	CAGGGAGGTGAATGTGAT
hsa_miR_1322	193	-15.6	CAGTGCCCCAGCCTGGCATCCTC	GATGATGCTGCTGATGCTG
hsa_miR_1343_5p	199	-24.2	CCCAGCCTGGCATCCTCTTCCTG	TGGGGAGCGGCCCCCGGGTGGG
hsa_miR_1343_5p	180	-18.76	GCCACCTCTTCCTCAGTGCCCCA	TGGGGAGCGGCCCCCGGGTGGG
hsa_miR_1343_5p	135	-15.3	CCTGTTCCAGAAGGACAGCCCCTC	TGGGGAGCGGCCCCCGGGTGGG
hsa_miR_135a_3p	280	-18.4	AGCTCCAGCTCTAACTCTGTC	TATAGGGATTGGAGCCGTGGCG
hsa_miR_139_3p	335	-20.2	ACTTCTCTGGGCCTCAGTTTTCT	TGGAGACGCGGCCCTGTTGGAGT
hsa_miR_139_3p	281	-19.5	GCTCCAGCTCTAACTCTGTCCCA	TGGAGACGCGGCCCTGTTGGAGT
hsa_miR_143_5p	191	-15.3	CTCAGTGCCCCAGCCTGGCATC	GGTGCAGTGCTGCATCTCTGGT
hsa_miR_1468_5p	419	-17.6	CAGCAGGACAGAGGAAACAGAA	CTCCGTTTGCCTGTTTCGCTG
hsa_miR_146a_3p	471	-18.1	AGAAAGGGCTGGAAGAAGCAGGGG	CCTCTGAAATTCAGTTCTTCAG
hsa_miR_146a_5p	337	-16.4	TTCTCTGGGCCTCAGTTTTCT	TGAGAACTGAATTCCATGGGTT
hsa_miR_146b_5p	336	-15.9	CTTCTCTGGGCCTCAGTTTTCT	TGAGAACTGAATTCCATAGGCT
hsa_miR_149_3p	147	-31.1	GGACAGCCCCTCATGCCTTCCCG	AGGGAGGGACGGGGGCTGTGC
hsa_miR_149_3p	281	-22.2	GCTCCAGCTCTAACTCTGTCCCA	AGGGAGGGACGGGGGCTGTGC
hsa_miR_149_3p	197	-21	GCCCCAGCCTGGCATCCTCTTCCT	AGGGAGGGACGGGGGCTGTGC
hsa_miR_149_3p	33	-16	GTGACTTCCTGGTGCCATCCTT	AGGGAGGGACGGGGGCTGTGC
hsa_miR_150_3p	124	-18.6	ATGTTCTACTTCCTGTTCCAG	CTGGTACAGGCCTGGGGGACAG
hsa_miR_151a_3p	190	-15.1	CCTCAGTGCCCCAGCCTGG	CTAGACTGAAGCTCCTTGAGG
hsa_miR_152_5p	477	-15.3	GGCTGGAAGAAGCAGGGGACCT	AGGTTCTGTGATACACTCCGACT
hsa_miR_154_5p	481	-15.8	GGAAGAAGCAGGGGACCTA	TAGGTTATCCGTGTTGCCTTCG
hsa_miR_1587	331	-16.6	CTTAACTTCTCTGGGCCTCAG	TTGGGCTGGGCTGGGTTGGG
hsa_miR_185_3p	10	-15.1	GGACAGCAGCAAACACTGCCCCC	AGGGGCTGGCTTTCCTCTGGTC
hsa_miR_1908_5p	38	-17.1	TTCCTGGTGCCATCCTTGGCT	CGGCGGGGACGGCGATTGGTC
hsa_miR_1908_5p	182	-16.7	CACCTCTTCCTCAGTGCCCCAGCCT	CGGCGGGGACGGCGATTGGTC
hsa_miR_1909_3p	202	-20.3	AGCCTGGCATCCTCTTCCTGCA	CGCAGGGGCCGGGTGCTCACCG
hsa_miR_1910_3p	212	-20.1	CCTCTTCCTGCATTCAGCCTT	GAGGCAGAAGCAGGATGACA
hsa_miR_1914_3p	135	-22.1	CCTGTTCCAGAAGGACAGCCCCTCA	GGAGGGGTCCCGCACTGGGAGG
hsa_miR_1914_3p	196	-20	TGCCCCAGCCTGGCATCCTCTTC	GGAGGGGTCCCGCACTGGGAGG
hsa_miR_1914_3p	212	-17.2	CCTCTTCCTGCATTCAGCCTTCT	GGAGGGGTCCCGCACTGGGAGG
hsa_miR_193b_5p	181	-23	CCACCTCTTCCTCAGTGCCCCA	CGGGGTTTTGAGGGCGAGATGA
hsa_miR_197_5p	37	-21.9	CTTCCTGGTGCCATCCTTGGCTCC	CGGGTAGAGAGGGCAGTGGGAGG
hsa_miR_197_5p	212	-21.3	CCTCTTCCTGCATTCAGCCTT	CGGGTAGAGAGGGCAGTGGGAGG
hsa_miR_197_5p	196	-18.7	TGCCCCAGCCTGGCATCCTCTTCCTG	CGGGTAGAGAGGGCAGTGGGAGG

hsa_miR_197_5p	280	-16.7	AGCTCCAGCTCTAACTCTGTCCCA	CGGGTAGAGAGGGCAGTGGGAGG
hsa_miR_1972	186	-19	TCTTCCTCAGTGCCCCAGCCTGG	TCAGGCCAGGCACAGTGGCTCA
hsa_miR_1976	475	-16.6	AGGGCTGGAAGAAGCAGGGGA	CCTCCTGCCCTCCTTGCTGT
hsa_miR_198	328	-21.9	ATGCTTAACTTCTCTGGGCC	GGTCCAGAGGGGAGATAGGTTC
hsa_miR_198	201	-16	CAGCCTGGCATCCTCTTCCTGCATT	GGTCCAGAGGGGAGATAGGTTC
hsa_miR_198	187	-15	CTTCCTCAGTGCCCCAGCCTGGCATC	GGTCCAGAGGGGAGATAGGTTC
hsa_miR_204_3p	150	-23.8	CAGCCCCTCATGCCTTCCCGC	GCTGGGAAGGCAAAGGGACGT
hsa_miR_204_3p	36	-19.6	ACTTCCTGGTGCCATCCTTGGC	GCTGGGAAGGCAAAGGGACGT
hsa_miR_204_3p	124	-15.1	ATGTTCTACTTCCTGTTCCAGA	GCTGGGAAGGCAAAGGGACGT
hsa_miR_204_5p	476	-18.6	GGGCTGGAAGAAGCAGGGGA	TTCCCTTTGTCATCCTATGCCT
hsa_miR_211_3p	150	-21.2	CAGCCCCTCATGCCTTCCCGC	GCAGGGACAGCAAAGGGGTGC
hsa_miR_211_3p	36	-16.1	ACTTCCTGGTGCCATCCTTGG	GCAGGGACAGCAAAGGGGTGC
hsa_miR_211_5p	476	-17.9	GGGCTGGAAGAAGCAGGGGA	TTCCCTTTGTCATCCTTCGCCT
hsa_miR_2116_5p	129	-15.1	CTACTTCCTGTTCCAGAAGGACA	GGTTCTTAGCATAGGAGGTCT
hsa_miR_219a_1_3p	277	-17.7	GTGAGCTCCAGCTCTAACTCT	AGAGTTGAGTCTGGACGTCCCG
hsa_miR_2277_5p	187	-23	CTTCCTCAGTGCCCCAGCCTGGCA	AGCGCGGGCTGAGCGCTGCCAGTC
hsa_miR_2355_3p	131	-15	ACTTCCTGTTCCAGAAGGACAGC	ATTGTCCTTGCTGTTTGGAGAT
hsa_miR_2392	202	-22.3	AGCCTGGCATCCTCTTCCTG	TAGGATGGGGGTGAGAGGTG
hsa_miR_2392	35	-15.8	GACTTCCTGGTGCCATCCTT	TAGGATGGGGGTGAGAGGTG
hsa_miR_2467_3p	204	-17.8	CCTGGCATCCTCTTCCTGCA	AGCAGAGGCAGAGAGGCTCAGG
hsa_miR_2467_3p	330	-17	GCTTAACTTCTCTGGGCCTCAGTT	AGCAGAGGCAGAGAGGCTCAGG
hsa_miR_2467_5p	327	-15.95	GATGCTTAACTTCTCTGGGCCTCA	TGAGGCTCTGTTAGCCTTGGCTC
hsa_miR_2682_3p	468	-15.9	CACAGAAAGGGCTGGAAGAAGCA	CGCCTCTTCAGCGCTGTCTTCC
hsa_miR_27a_5p	329	-16.42	TGCTTAACTTCTCTGGGCCTC	AGGGCTTAGCTGCTTGTGAGCA
hsa_miR_2861	181	-22.4	CCACCTCTTCCTCAGTGCCCC	GGGGCCTGGCGGTGGGCGG
hsa_miR_296_3p	147	-21.5	GGACAGCCCCTCATGCCTTC	GAGGGTTGGGTGGAGGCTCTCC
hsa_miR_296_3p	212	-21.4	CCTCTTCCTGCATTCAGCCTTC	GAGGGTTGGGTGGAGGCTCTCC
hsa_miR_296_3p	276	-19.5	CGTGAGCTCCAGCTCTAACTCTG	GAGGGTTGGGTGGAGGCTCTCC
hsa_miR_296_5p	478	-15.3	GCTGGAAGAAGCAGGGGACCTA	AGGGCCCCCCCTCAATCCTGT
hsa_miR_3074_5p	475	-17	AGGGCTGGAAGAAGCAGGGGA	GTTCCTGCTGAACTGAGCCAG
hsa_miR_30b_3p	15	-15.6	GCAGCAAACACTGCCCCCGT	CTGGGAGGTGGATGTTTACTTC
hsa_miR_30c_1_3p	283	-20.2	TCCAGCTCTAACTCTGTCCCAA	CTGGGAGAGGGTTGTTTACTCC
hsa_miR_3120_3p	293	-15.8	ACTCTGTCCCAATCAGCTGTG	CACAGCAAGTGTAGACAGGCA
hsa_miR_3122	180	-18.8	GCCACCTCTTCCTCAGTGCCCCAGC	GTTGGGACAAGAGGACGGTCTT
hsa_miR_3122	284	-17.7	CCAGCTCTAACTCTGTCCCAAT	GTTGGGACAAGAGGACGGTCTT
hsa_miR_3126_5p	202	-18.4	AGCCTGGCATCCTCTTCCTGCA	TGAGGGACAGATGCCAGAAGCA
hsa_miR_3131	35	-17.7	GACTTCCTGGTGCCATCCTTGG	TCGAGGACTGGTGGAAGGGCCTT
hsa_miR_3132	337	-20	TTCTCTGGGCCTCAGTTTTCTCATCTG	TGGGTAGAGAAGGAGCTCAGAGGA
hsa_miR_3132	279	-19.9	GAGCTCCAGCTCTAACTCTGTCCCA	TGGGTAGAGAAGGAGCTCAGAGGA
hsa_miR_3135a	193	-20.3	CAGTGCCCCAGCCTGGCA	TGCCTAGGCTGAGACTGCAGTG
hsa_miR_3135b	270	-17.3	AGGCAACGTGAGCTCCAGCT	GGCTGGAGCGAGTGCAGTGGTG
hsa_miR_3135b	209	-16.3	CATCCTCTTCCTGCATTCAGCC	GGCTGGAGCGAGTGCAGTGGTG
hsa_miR_3135b	187	-15.6	CTTCCTCAGTGCCCCAGCC	GGCTGGAGCGAGTGCAGTGGTG
hsa_miR_3135b	202	-15	AGCCTGGCATCCTCTTCCTGCA	GGCTGGAGCGAGTGCAGTGGTG
hsa_miR_3138	281	-22	GCTCCAGCTCTAACTCTGTCCCA	TGTGGACAGTGAGGTAGAGGGAGT
hsa_miR_3138	122	-19.3	GAATGTTCTACTTCCTGTTCCA	TGTGGACAGTGAGGTAGAGGGAGT
hsa_miR_3141	214	-25.2	TCTTCCTGCATTCAGCCTTC	GAGGGCGGGTGGAGGAGGA
hsa_miR_3141	208	-18.7	GCATCCTCTTCCTGCATTC	GAGGGCGGGTGGAGGAGGA
hsa_miR_3147	181	-32.8	CCACCTCTTCCTCAGTGCCCCAGCC	GGTTGGGCAGTGAGGAGGGTGTGA
hsa_miR_3147	110	-20.5	ACATGCCCTTCAGAATGTTCTACT	GGTTGGGCAGTGAGGAGGGTGTGA
hsa_miR_3147	279	-18.9	[illegible]AGCTCCAGCTCTAACTCTGTCCCAAT[illegible]	GGTTGGGCAGTGAGGAGGGTGTGA
hsa_miR_3150a_3p	181	-18.57	CCACCTCTTCCTCAGTGCCCCAG	CTGGGGAGATCCTCGAGGTTGG
hsa_miR_3150b_3p	200	-19.7	CCAGCCTGGCATCCTCTTCC	TGAGGAGATCGTCGAGGTTGG
hsa_miR_3151_5p	183	-26.1	ACCTCTTCCTCAGTGCCCCAGCC	GGTGGGGCAATGGGATCAGGT
hsa_miR_3152_3p	192	-17.6	TCAGTGCCCCAGCCTGGCATC	TGTGTTAGAATAGGGGCAATAA
hsa_miR_3153	122	-16.4	GAATGTTCTACTTCCTGTTCCA	GGGGAAAGCGAGTAGGGACATTT
hsa_miR_3153	148	-15.7	GACAGCCCCTCATGCCTTCCCG	GGGGAAAGCGAGTAGGGACATTT
hsa_miR_3154	198	-18.6	CCCCAGCCTGGCATCCTCTTCCTG	CAGAAGGGGAGTTGGGAGCAGA
hsa_miR_3154	218	-17.6	CCTGCATTCAGCCTTCTTTTC	CAGAAGGGGAGTTGGGAGCAGA
hsa_miR_3154	340	-16.4	TCTGGGCCTCAGTTTTCTCATCTG	CAGAAGGGGAGTTGGGAGCAGA
hsa_miR_3154	211	-16.1	TCCTCTTCCTGCATTCAGCCTTCTT	CAGAAGGGGAGTTGGGAGCAGA
hsa_miR_3155a	186	-22.8	TCTTCCTCAGTGCCCCAGCCTGG	CCAGGCTCTGCAGTGGGAACT
hsa_miR_3155b	189	-21.9	TCCTCAGTGCCCCAGCCTGG	CCAGGCTCTGCAGTGGGA
hsa_miR_3160_3p	332	-16.2	TTAACTTCTCTGGGCCTCAGTTTT	AGAGCTGAGACTAGAAAGCCCA
hsa_miR_3162_5p	117	-16.6	CTTCAGAATGTTCTACTTCCTGT	TTAGGGAGTAGAAGGGTGGGGAG
hsa_miR_3162_5p	344	-16.3	GGCCTCAGTTTTCTCATCTGTGA	TTAGGGAGTAGAAGGGTGGGGAG
hsa_miR_3166	342	-15.86	TGGGCCTCAGTTTTCTCATCTGTG	CGCAGACAATGCCTACTGGCCTA
hsa_miR_3170	182	-22.3	CACCTCTTCCTCAGTGCCCCAG	CTGGGGTTCTGAGACAGACAGT
hsa_miR_3170	330	-18.3	GCTTAACTTCTCTGGGCCTCAG	CTGGGGTTCTGAGACAGACAGT
hsa_miR_3173_3p	215	-17.9	CTTCCTGCATTCAGCCTTCTTT	AAAGGAGGAAATAGGCAGGCCA
hsa_miR_3175	116	-15.8	CCTTCAGAATGTTCTACTTCCTG	CGGGGAGAGAACGCAGTGACGT
hsa_miR_3177_3p	294	-20.1	CTCTGTCCCAATCAGCTGTGTG	TGCACGGCACTGGGGACACGT
hsa_miR_3177_3p	193	-19.7	CAGTGCCCCAGCCTGGCA	TGCACGGCACTGGGGACACGT
hsa_miR_3180_3p	179	-20.8	CGCCACCTCTTCCTCAGTGCCCCA	TGGGGCGGAGCTTCCGGAGGCC
hsa_miR_3180_3p	196	-15.9	TGCCCCAGCCTGGCATCCTCTTCC	TGGGGCGGAGCTTCCGGAGGCC
hsa_miR_3180_3p	210	-15.4	ATCCTCTTCCTGCATTCAGCCTTC	TGGGGCGGAGCTTCCGGAGGCC
hsa_miR_3181	192	-15.6	TCAGTGCCCCAGCCTGGC	ATCGGGCCCTCGGCGCCGG
hsa_miR_3184_5p	134	-18.2	TCCTGTTCCAGAAGGACAGCCCCTCA	TGAGGGGCCTCAGACCGAGCTTTT

hsa_miR_3185	199	-18.2	CCCAGCCTGGCATCCTCTTCC	AGAAGAAGGCGGTCGGTCTGCGG
hsa_miR_3188	212	-21.1	CCTCTTCCTGCATTCAGCCTTC	AGAGGCTTTGTGCGGATACGGGG
hsa_miR_3188	134	-15.7	TCCTGTTCCAGAAGGACAGCCCCT	AGAGGCTTTGTGCGGATACGGGG
hsa_miR_3190_3p	338	-19.7	TCTCTGGGCCTCAGTTTTCTCATC	TGTGGAAGGTAGACGGCCAGAGA
hsa_miR_3190_3p	146	-15.9	AGGACAGCCCCTCATGCCTTCCCG	TGTGGAAGGTAGACGGCCAGAGA
hsa_miR_3190_3p	202	-15.6	AGCCTGGCATCCTCTTCCTGCA	TGTGGAAGGTAGACGGCCAGAGA
hsa_miR_3191_3p	192	-15.2	TCAGTGCCCCAGCCTGGCATCCTCT	TGGGGACGTAGCTGGCCAGACAG
hsa_miR_3196	284	-15.4	CCAGCTCTAACTCTGTCCCA	CGGGGCGGCAGGGGCCTC
hsa_miR_3197	334	-19.6	AACTTCTCTGGGCCTCAGTTTTC	GGAGGCGCAGGCTCGGAAAGGCG
hsa_miR_3197	179	-18.6	CGCCACCTCTTCCTCAGTGCCCC	GGAGGCGCAGGCTCGGAAAGGCG
hsa_miR_3197	329	-17.8	TGCTTAACTTCTCTGGGCCTCA	GGAGGCGCAGGCTCGGAAAGGCG
hsa_miR_3197	210	-17.6	ATCCTCTTCCTGCATTCAGCCTTC	GGAGGCGCAGGCTCGGAAAGGCG
hsa_miR_3197	38	-15	TTCCTGGTGCCATCCTTGGCTCC	GGAGGCGCAGGCTCGGAAAGGCG
hsa_miR_3198	44	-18.4	GTGCCATCCTTGGCTCCAT	GTGGAGTCCTGGGGAATGGAGA
hsa_miR_3199	133	-17.2	TTCCTGTTCCAGAAGGACAGCCCCT	AGGGACTGCCTTAGGAGAAAGTT
hsa_miR_320a	334	-18.8	AACTTCTCTGGGCCTCAGTTTTC	AAAAGCTGGGTTGAGAGGGCGA
hsa_miR_320b	334	-18.8	AACTTCTCTGGGCCTCAGTTTTC	AAAAGCTGGGTTGAGAGGGCAA
hsa_miR_320c	336	-18.8	CTTCTCTGGGCCTCAGTTTTC	AAAAGCTGGGTTGAGAGGGT
hsa_miR_320d	337	-19.6	TTCTCTGGGCCTCAGTTTTC	AAAAGCTGGGTTGAGAGGA
hsa_miR_320e	335	-17	ACTTCTCTGGGCCTCAGTTTT	AAAGCTGGGTTGAGAAGG
hsa_miR_323a_5p	478	-16.1	GCTGGAAGAAGCAGGGGACCTACTG	AGGTGGTCCGTGGCGCGTTCGC
hsa_miR_328_5p	146	-33.7	AGGACAGCCCCTCATGCCTTCCC	GGGGGGGCAGGAGGGGCTCAGGG
hsa_miR_331_5p	35	-22.1	GACTTCCTGGTGCCATCCTTGG	CTAGGTATGGTCCCAGGGATCC
hsa_miR_342_5p	134	-17.8	TCCTGTTCCAGAAGGACAGCCCCT	AGGGGTGCTATCTGTGATTGA
hsa_miR_342_5p	192	-15.9	TCAGTGCCCCAGCCTGGCATCCTC	AGGGGTGCTATCTGTGATTGA
hsa_miR_345_3p	179	-15.4	CGCCACCTCTTCCTCAGTGC	GCCCTGAACGAGGGGTCTGGAG
hsa_miR_346	474	-15.6	AAGGGCTGGAAGAAGCAGGGGACC	TGTCTGCCCGCATGCCTGCCTCT
hsa_miR_34a_5p	12	-20.3	ACAGCAGCAAACACTGCCC	TGGCAGTGTCTTAGCTGGTTGT
hsa_miR_34a_5p	281	-17	GCTCCAGCTCTAACTCTGTCC	TGGCAGTGTCTTAGCTGGTTGT
hsa_miR_34c_5p	8	-19.1	CTGGACAGCAGCAAACACTGCCC	AGGCAGTGTAGTTAGCTGATTGC
hsa_miR_3605_5p	35	-22	GACTTCCTGGTGCCATCCTTG	TGAGGATGGATAGCAAGGAAGCC
hsa_miR_3605_5p	213	-17.8	CTCTTCCTGCATTCAGCCTTC	TGAGGATGGATAGCAAGGAAGCC
hsa_miR_3610	206	-15.2	TGGCATCCTCTTCCTGCATTC	GAATCGGAAAGGAGGCGCCG
hsa_miR_3612	31	-15.2	CCGTGACTTCCTGGTGCCATCCT	AGGAGGCATCTTGAGAAATGGA
hsa_miR_3616_3p	98	-15.7	GAAGAACGTGTGACATGCCCTTC	CGAGGGCATTTCATGATGCAGGC
hsa_miR_3616_3p	203	-15.42	GCCTGGCATCCTCTTCCTGCATTCA	CGAGGGCATTTCATGATGCAGGC
hsa_miR_3620_5p	476	-18.2	GGGCTGGAAGAAGCAGGGGACCTAC	GTGGGCTGGGCTGGGCTGGGCC
hsa_miR_3621	346	-15.8	CCTCAGTTTTCTCATCTGTG	CGCGGGTCGGGGTCTGCAGG
hsa_miR_3622a_3p	474	-16.7	AAGGGCTGGAAGAAGCAGGGGA	TCACCTGACCTCCCATGCCTGT
hsa_miR_3622b_3p	475	-17.1	AGGGCTGGAAGAAGCAGGGGA	TCACCTGAGCTCCCGTGCCTG
hsa_miR_3622b_5p	145	-18.1	AAGGACAGCCCCTCATGCCT	AGGCATGGGAGGTCAGGTGA
hsa_miR_3622b_5p	442	-15.3	GTACATGACCGATGATGTCT	AGGCATGGGAGGTCAGGTGA
hsa_miR_3652	189	-24.5	TCCTCAGTGCCCCAGCCT	CGGCTGGAGGTGTGAGGA
hsa_miR_3656	200	-22	CCAGCCTGGCATCCTCT	GGCGGGTGCGGGGGTGG
hsa_miR_3656	212	-20.3	CCTCTTCCTGCATTCAGCC	GGCGGGTGCGGGGGTGG
hsa_miR_3656	284	-15.1	CCAGCTCTAACTCTGTC	GGCGGGTGCGGGGGTGG
hsa_miR_365a_5p	109	-17.6	GACATGCCCTTCAGAATGTTCTA	AGGGACTTTTGGGGGCAGATGTG
hsa_miR_365b_5p	110	-16.2	ACATGCCCTTCAGAATGTTCTA	AGGGACTTTCAGGGGCAGCTGT
hsa_miR_3665	294	-16.6	CTCTGTCCCAATCAGCTGTG	AGCAGGTGCGGGGCGGCG
hsa_miR_3666	205	-16.1	CTGGCATCCTCTTCCTGCATTC	CAGTGCAAGTGTAGATGCCGA
hsa_miR_3679_5p	113	-15.6	TGCCCTTCAGAATGTTCTACTTCC	TGAGGATATGGCAGGGAAGGGGA
hsa_miR_3691_5p	195	-16.1	GTGCCCCAGCCTGGCATCCTCT	AGTGGATGATGGAGACTCGGTAC
hsa_miR_3692_5p	123	-21.9	AATGTTCTACTTCCTGTTCCAGAAGG	CCTGCTGGTCAGGAGTGGATACTG
hsa_miR_3714	144	-16.1	GAAGGACAGCCCCTCATGCCTTC	GAAGGCAGCAGTGCTCCCCTGT
hsa_miR_377_5p	197	-16.4	GCCCCAGCCTGGCATCCTCT	AGAGGTTGCCCTTGGTGAATTC
hsa_miR_378a_5p	469	-20	ACAGAAAGGGCTGGAAGAAGCAGGGG	CTCCTGACTCCAGGTCCTGTGT
hsa_miR_378g	331	-19.3	CTTAACTTCTCTGGGCCTCAGT	ACTGGGCTTGGAGTCAGAAG
hsa_miR_378i	125	-16.3	TGTTCTACTTCCTGTTCCAGA	ACTGGACTAGGAGTCAGAAGG
hsa_miR_3909	476	-17.3	GGGCTGGAAGAAGCAGGGGACC	TGTCCTCTAGGGCCTGCAGTCT
hsa_miR_3911	151	-17	AGCCCCTCATGCCTTCCCGCG	TGTGTGGATCCTGGAGGAGGCA
hsa_miR_3915	174	-15.9	CCACACGCCACCTCTTCCTCAG	TTGAGGAAAAGATGGTCTTATT
hsa_miR_3916	193	-21.1	CAGTGCCCCAGCCTGGCATCCTCTT	AAGAGGAAGAAATGGCTGGTTCTCAG
hsa_miR_3927_3p	112	-17.4	ATGCCCTTCAGAATGTTCTACTTC	CAGGTAGATATTTGATAGGCAT
hsa_miR_3928_3p	197	-17	GCCCCAGCCTGGCATCCTCT	GGAGGAACCTTGGAGCTTCGGC
hsa_miR_3929	209	-16.5	CATCCTCTTCCTGCATTCAGCCTT	GAGGCTGATGTGAGTAGACCACT
hsa_miR_3934_3p	189	-22.6	TCCTCAGTGCCCCAGCCTGGCA	TGCTCAGGTTGCACAGCTGGGA
hsa_miR_3934_5p	343	-27.6	GGGCCTCAGTTTTCTCATCTGT	TCAGGTGTGGAAACTGAGGCAG
hsa_miR_3934_5p	187	-22.6	CTTCCTCAGTGCCCCAGCCTGG	TCAGGTGTGGAAACTGAGGCAG
hsa_miR_3936	196	-16.3	TGCCCCAGCCTGGCATCCTCTTC	TAAGGGGTGTATGGCAGATGCA
hsa_miR_3937	116	-16.1	CCTTCAGAATGTTCTACTTCCTGT	ACAGGCGGCTGTAGCAATGGGGG
hsa_miR_3940_5p	290	-18.3	CTAACTCTGTCCCAATCAGC	GTGGGTTGGGGCGGGCTCTG
hsa_miR_3944_3p	187	-19.4	CTTCCTCAGTGCCCCAGCCTGGC	TTCGGGCTGGCCTGCTGCTCCGG
hsa_miR_3945	146	-20.5	AGGACAGCCCCTCATGCCTT	AGGGCATAGGAGAGGGTTGATAT
hsa_miR_3945	281	-15.8	GCTCCAGCTCTAACTCTGTCCC	AGGGCATAGGAGAGGGTTGATAT
hsa_miR_3972	465	-15.5	GACCACAGAAAGGGCTGGAAG	CTGCCAGCCCCGTTCCAGGGCA
hsa_miR_3978	335	-16.2	ACTTCTCTGGGCCTCAGTTTTCTCAT	GTGGAAAGCATGCATCCAGGGTGT
hsa_miR_4253	183	-17.5	ACCTCTTCCTCAGTGCCCC	AGGGCATGTCCAGGGGGT
hsa_miR_4253	198	-17.4	CCCCAGCCTGGCATCCTCT	AGGGCATGTCCAGGGGGT

hsa_miR_4253	36	-16.1	ACTTCCTGGTGCCATCCTT	AGGGCATGTCCAGGGGGT
hsa_miR_4257	347	-18	CTCAGTTTTCTCATCTGTGA	CCAGAGGTGGGGACTGAG
hsa_miR_4259	183	-17.4	ACCTCTTCCTCAGTGCCCCAGCCTG	CAGTTGGGTCTAGGGGTCAGGA
hsa_miR_4260	186	-18.3	TCTTCCTCAGTGCCCCAGC	CTTGGGGCATGGAGTCCCA
hsa_miR_4260	289	-18	TCTAACTCTGTCCCAAT	CTTGGGGCATGGAGTCCCA
hsa_miR_4265	191	-15.3	CTCAGTGCCCCAGCCTGGCAT	CTGTGGGCTCAGCTCTGGG
hsa_miR_4270	203	-18.5	GCCTGGCATCCTCTTCCTGC	TCAGGGAGTCAGGGGAGGGC
hsa_miR_4271	198	-20.7	CCCCAGCCTGGCATCCTCT	GGGGGAAGAAAAGGTGGGG
hsa_miR_4281	196	-20.5	TGCCCCAGCCTGGCATCC	GGGTCCCGGGGAGGGGGG
hsa_miR_4281	332	-20.1	TTAACTTCTCTGGGCCT	GGGTCCCGGGGAGGGGGG
hsa_miR_4283	333	-18.9	TAACTTCTCTGGGCCTCA	TGGGGCTCAGCGAGTTT
hsa_miR_4289	108	-17.2	TGACATGCCCTTCAGAATGT	GCATTGTGCAGGGCTATCA
hsa_miR_4298	181	-27.5	CCACCTCTTCCTCAGTGCCCCAG	CTGGGACAGGAGGAGGAGGCAG
hsa_miR_4298	124	-25.3	ATGTTCTACTTCCTGTTCCAG	CTGGGACAGGAGGAGGAGGCAG
hsa_miR_4298	150	-22.2	CAGCCCCTCATGCCTTCCCGC	CTGGGACAGGAGGAGGAGGCAG
hsa_miR_4298	284	-20.1	CCAGCTCTAACTCTGTCCCAA	CTGGGACAGGAGGAGGAGGCAG
hsa_miR_4298	112	-17.7	ATGCCCTTCAGAATGTTCTAC	CTGGGACAGGAGGAGGAGGCAG
hsa_miR_4299	189	-15.4	TCCTCAGTGCCCCAGC	GCTGGTGACATGAGAGGC
hsa_miR_431_3p	481	-18.5	GGAAGAAGCAGGGGACCTA	CAGGTCGTCTTGCAGGGCTTCT
hsa_miR_4311	220	-18.6	TGCATTCAGCCTTCTTTT	GAAAGAGAGCTGAGTGTG
hsa_miR_4322	190	-18.4	CCTCAGTGCCCCAGCCTGGCAT	CTGTGGGCTCAGCGCGTGGGG
hsa_miR_4322	331	-15.8	CTTAACTTCTCTGGGCCTCAG	CTGTGGGCTCAGCGCGTGGGG
hsa_miR_4326	417	-17.4	GCCAGCAGGACAGAGGAAACA	TGTTCCTCTGTCTCCCAGAC
hsa_miR_4419a	207	-15.4	GGCATCCTCTTCCTGCA	TGAGGGAGGAGACTGCA
hsa_miR_4420	347	-16.9	CTCAGTTTTCTCATCTGTGAA	GTCACTGATGTCTGTAGCTGAG
hsa_miR_4429	336	-20.8	CTTCTCTGGGCCTCAGTTTTC	AAAAGCTGGGCTGAGAGGCG
hsa_miR_4430	282	-17.6	CTCCAGCTCTAACTCTGTCCC	AGGCTGGAGTGAGCGGAG
hsa_miR_4430	213	-17.1	CTCTTCCTGCATTCAGCCT	AGGCTGGAGTGAGCGGAG
hsa_miR_4433a_3p	203	-16.6	GCCTGGCATCCTCTTCCTGC	ACAGGAGTGGGGGTGGGACAT
hsa_miR_4433b_3p	145	-20.7	AAGGACAGCCCCTCATGCCTT	CAGGAGTGGGGGGTGGGACGT
hsa_miR_4437	329	-17.8	TGCTTAACTTCTCTGGGCCTC	TGGGCTCAGGGTACAAAGGTT
hsa_miR_4443	290	-15.1	CTAACTCTGTCCCAA	TTGGAGGCGTGGGTTTT
hsa_miR_4446_3p	293	-17.6	ACTCTGTCCCAATCAGCTGTG	CAGGGCTGGCAGTGACATGGGT
hsa_miR_4446_3p	273	-16.4	CAACGTGAGCTCCAGCTCTA	CAGGGCTGGCAGTGACATGGGT
hsa_miR_4446_3p	198	-15.3	CCCCAGCCTGGCATCCTC	CAGGGCTGGCAGTGACATGGGT
hsa_miR_4447	279	-15.1	GAGCTCCAGCTCTAACT	GGTGGGGGCTGTTGTTT
hsa_miR_4450	328	-19.5	ATGCTTAACTTCTCTGGGCCTCA	TGGGGATTTGGAGAAGTGGTGA
hsa_miR_4454	42	-21.8	TGGTGCCATCCTTGGCTCC	GGATCCGAGTCACGGCACCA
hsa_miR_4454	193	-15.7	CAGTGCCCCAGCCTGGCATCC	GGATCCGAGTCACGGCACCA
hsa_miR_4459	198	-20.1	CCCCAGCCTGGCATCCTCTTCCTGC	CCAGGAGGCGGAGGAGGTGGAG
hsa_miR_4459	117	-17.9	CTTCAGAATGTTCTACTTCCTGT	CCAGGAGGCGGAGGAGGTGGAG
hsa_miR_4461	281	-19.9	GCTCCAGCTCTAACTCTGTCCCAATC	GATTGAGACTAGTAGGGCTAGGC
hsa_miR_4461	330	-18.1	GCTTAACTTCTCTGGGCCTCAGTT	GATTGAGACTAGTAGGGCTAGGC
hsa_miR_4463	337	-16.3	TTCTCTGGGCCTCAGTTTT	GAGACTGGGGTGGGGCC
hsa_miR_4466	37	-17.6	CTTCCTGGTGCCATCC	GGGTGCGGGCCGGCGGGG
hsa_miR_4466	210	-15	ATCCTCTTCCTGCATTC	GGGTGCGGGCCGGCGGGG
hsa_miR_4467	285	-19.8	CAGCTCTAACTCTGTCCCA	TGGCGGCGGTAGTTATGGGCTT
hsa_miR_4467	13	-16.8	CAGCAGCAAACACTGCCCCCG	TGGCGGCGGTAGTTATGGGCTT
hsa_miR_4472	143	-19.1	AGAAGGACAGCCCCTCATG	GGTGGGGGGTGTTGTTTT
hsa_miR_4478	213	-20.5	CTCTTCCTGCATTCAGCCTT	GAGGCTGAGCTGAGGAG
hsa_miR_4478	336	-16.8	CTTCTCTGGGCCTCAGTTTT	GAGGCTGAGCTGAGGAG
hsa_miR_4479	296	-15.2	CTGTCCCAATCAGCTGTGTG	CGCGCGGCCGTGCTCGGAGCAG
hsa_miR_4488	202	-16.6	AGCCTGGCATCCTCTTCCT	AGGGGGCGGGCTCCGGCG
hsa_miR_4489	215	-20.1	CTTCCTGCATTCAGCCTTC	TGGGGCTAGTGATGCAGGACG
hsa_miR_4489	281	-16.2	GCTCCAGCTCTAACTCTGTCCCA	TGGGGCTAGTGATGCAGGACG
hsa_miR_4489	184	-16.1	CCTCTTCCTCAGTGCCCCA	TGGGGCTAGTGATGCAGGACG
hsa_miR_4492	192	-21.8	TCAGTGCCCCAGCCTG	GGGGCTGGGCGCGCGCC
hsa_miR_4492	276	-19.2	CGTGAGCTCCAGCTCT	GGGGCTGGGCGCGCGCC
hsa_miR_4494	184	-17.5	CCTCTTCCTCAGTGCCCCAGCCTGG	CCAGACTGTGGCTGACCAGAGG
hsa_miR_4496	337	-26.7	TTCTCTGGGCCTCAGTTTTCTC	GAGGAAACTGAAGCTGAGAGGG
hsa_miR_4496	211	-18.9	TCCTCTTCCTGCATTCAGCCTTCTTT	GAGGAAACTGAAGCTGAGAGGG
hsa_miR_449a	11	-22.4	GACAGCAGCAAACACTGCCC	TGGCAGTGTATTGTTAGCTGGT
hsa_miR_449b_5p	11	-22.4	GACAGCAGCAAACACTGCCC	AGGCAGTGTATTGTTAGCTGGC
hsa_miR_449c_5p	200	-17	CCAGCCTGGCATCCTCTTCCTG	TAGGCAGTGTATTGCTAGCGGCTGT
hsa_miR_4505	295	-23.2	TCTGTCCCAATCAGCTG	AGGCTGGGCTGGGACGGA
hsa_miR_4505	211	-18.4	TCCTCTTCCTGCATTCAGCCT	AGGCTGGGCTGGGACGGA
hsa_miR_4505	337	-17	TTCTCTGGGCCTCAGTTT	AGGCTGGGCTGGGACGGA
hsa_miR_4508	194	-26.7	AGTGCCCCAGCCTGGC	GCGGGGCTGGGCGCGCG
hsa_miR_4510	202	-22.5	AGCCTGGCATCCTCTTCCTGCA	TGAGGGAGTAGGATGTATGGTT
hsa_miR_4516	203	-19.4	GCCTGGCATCCTCTTCC	GGGAGAAGGGTCGGGGC
hsa_miR_4525	198	-22.6	CCCCAGCCTGGCATCCTCTT	GGGGGGATGTGCATGCTGGTT
hsa_miR_4525	13	-21	CAGCAGCAAACACTGCCCCC	GGGGGGATGTGCATGCTGGTT
hsa_miR_4525	139	-19.9	TTCCAGAAGGACAGCCCCTC	GGGGGGATGTGCATGCTGGTT
hsa_miR_4525	282	-15.8	CTCCAGCTCTAACTCTGTCC	GGGGGGATGTGCATGCTGGTT
hsa_miR_4530	329	-15.6	TGCTTAACTTCTCTGGG	CCCAGCAGGACGGGAGCG
hsa_miR_4533	202	-22.1	AGCCTGGCATCCTCTTCCT	TGGAAGGAGGTTGCCGGACGCT
hsa_miR_4533	280	-17	AGCTCCAGCTCTAACTCTGTCCC	TGGAAGGAGGTTGCCGGACGCT
hsa_miR_4534	210	-17	ATCCTCTTCCTGCATTC	GGATGGAGGAGGGGTCT

hsa_miR_4534	285	-16.9	CAGCTCTAACTCTGTCC	GGATGGAGGAGGGGTCT
hsa_miR_4534	343	-16.6	GGGCCTCAGTTTTCTCATCT	GGATGGAGGAGGGGTCT
hsa_miR_4535	44	-15.9	GTGCCATCCTTGGCTCCAT	GTGGACCTGGCTGGGAC
hsa_miR_4537	38	-16.1	TTCCTGGTGCCATCCTTGGCTCC	TGAGCCGAGCTGAGCTTAGCTG
hsa_miR_4632_5p	199	-19.1	CCCAGCCTGGCATCCTCTTCCTGCATTC	GAGGGCAGCGTGGGTGTGGCGGA
hsa_miR_4634	39	-16.7	TCCTGGTGCCATCCTTGGCTC	CGGCGCGACCGGCCCGGGG
hsa_miR_4638_5p	293	-18.2	ACTCTGTCCCAATCAGCTGTGT	ACTCGGCTGCGGTGGACAAGT
hsa_miR_4640_5p	37	-25.3	CTTCCTGGTGCCATCCTTGGCTCCA	TGGGCCAGGGAGCAGCTGGTGGG
hsa_miR_4640_5p	280	-22.2	AGCTCCAGCTCTAACTCTGTCCCA	TGGGCCAGGGAGCAGCTGGTGGG
hsa_miR_4640_5p	8	-15.9	CTGGACAGCAGCAAACACTGCCCC	TGGGCCAGGGAGCAGCTGGTGGG
hsa_miR_4644	338	-16.1	TCTCTGGGCCTCAGTTTTCTCATCTG	TGGAGAGAGAAAAGAGACAGAAG
hsa_miR_4646_5p	173	-19.1	GCCACACGCCACCTCTTCCTCAGT	ACTGGGAAGAGGAGCTGAGGGA
hsa_miR_4647	196	-22.3	TGCCCCAGCCTGGCATCCTCTTC	GAAGATGGTGCTGTGCTGAGGAA
hsa_miR_4648	152	-17.1	GCCCCTCATGCCTTCCCGCG	TGTGGGACTGCAAATGGGAG
hsa_miR_4649_5p	171	-18.9	GGGCCACACGCCACCTCTTCCTC	TGGGCGAGGGGTGGGCTCTCAGAG
hsa_miR_4649_5p	195	-16	GTGCCCCAGCCTGGCATCCTCTTCCT	TGGGCGAGGGGTGGGCTCTCAGAG
hsa_miR_4651	203	-21.7	GCCTGGCATCCTCTTCCTG	CGGGGTGGGTGAGGTCGGGC
hsa_miR_4651	212	-16.6	CCTCTTCCTGCATTCAGCCTT	CGGGGTGGGTGAGGTCGGGC
hsa_miR_4653_3p	37	-25	CTTCCTGGTGCCATCCTTGGCTCCA	TGGAGTTAAGGGTTGCTTGGAGA
hsa_miR_4654	341	-15.9	CTGGGCCTCAGTTTTCTCATC	TGTGGGATCTGGAGGCATCTGG
hsa_miR_4656	208	-26.7	GCATCCTCTTCCTGCATTCAGCCTT	TGGGCTGAGGGCAGGAGGCCTGT
hsa_miR_4656	185	-26.2	CTCTTCCTCAGTGCCCCAGCCTG	TGGGCTGAGGGCAGGAGGCCTGT
hsa_miR_4656	333	-19.5	TAACTTCTCTGGGCCTCAGTTTT	TGGGCTGAGGGCAGGAGGCCTGT
hsa_miR_4656	341	-19	CTGGGCCTCAGTTTTCTCATCTG	TGGGCTGAGGGCAGGAGGCCTGT
hsa_miR_4657	201	-18.8	CAGCCTGGCATCCTCTTCCTGCATT	AATGTGGAAGTGGTCTGAGGCAT
hsa_miR_4658	138	-17.1	GTTCCAGAAGGACAGCCCCTCAT	GTGAGTGTGGATCCTGGAGGAAT
hsa_miR_4660	38	-15.3	TTCCTGGTGCCATCCTTGGCTCCA	TGCAGCTCTGGTGGAAAATGGAG
hsa_miR_4663	330	-18.4	GCTTAACTTCTCTGGGCCTCAGTT	AGCTGAGCTCCATGGACGTGCAGT
hsa_miR_4665_5p	15	-17.4	GCAGCAAACACTGCCCCCGT	CTGGGGGACGCGTGAGCGCGAGC
hsa_miR_4665_5p	102	-15.5	AACGTGTGACATGCCCTTCAG	CTGGGGGACGCGTGAGCGCGAGC
hsa_miR_4667_3p	472	-15.7	GAAAGGGCTGGAAGAAGCAGGGG	TCCCTCCTTCTGTCCCCACAG
hsa_miR_4667_5p	332	-18.7	TTAACTTCTCTGGGCCTCAGT	ACTGGGGAGCAGAAGGAGAACC
hsa_miR_4667_5p	183	-18.4	ACCTCTTCCTCAGTGCCCCAGC	ACTGGGGAGCAGAAGGAGAACC
hsa_miR_4674	329	-17	TGCTTAACTTCTCTGGGCCTCAG	CTGGGCTCGGGACGCGCGGCT
hsa_miR_4675	135	-22.5	CCTGTTCCAGAAGGACAGCCCC	GGGGCTGTGATTGACCAGCAGG
hsa_miR_4675	37	-18.2	CTTCCTGGTGCCATCCTTGGCTCC	GGGGCTGTGATTGACCAGCAGG
hsa_miR_4676_5p	192	-15.9	TCAGTGCCCCAGCCTGGCATC	GAGCCAGTGGTGAGACAGTGA
hsa_miR_4687_3p	134	-23.1	TCCTGTTCCAGAAGGACAGCCC	TGGCTGTTGGAGGGGGCAGGC
hsa_miR_4689	176	-18.5	ACACGCCACCTCTTCCTCAG	TTGAGGAGACATGGTGGGGGCC
hsa_miR_4689	151	-18.4	AGCCCCTCATGCCTTCCCGC	TTGAGGAGACATGGTGGGGGCC
hsa_miR_4690_5p	337	-18.6	TTCTCTGGGCCTCAGTTTTCTC	GAGCAGGCGAGGCTGGGCTGAA
hsa_miR_4692	190	-17.1	CCTCAGTGCCCCAGCCTGG	TCAGGCAGTGTGGGTATCAGAT
hsa_miR_4695_5p	200	-16.8	CCAGCCTGGCATCCTCTTCCTG	CAGGAGGCAGTGGGCGAGCAGG
hsa_miR_4697_5p	137	-16.4	TGTTCCAGAAGGACAGCCCCTC	AGGGGGCGCAGTCACTGACGTG
hsa_miR_4697_5p	100	-15.5	AGAACGTGTGACATGCCCTTCA	AGGGGGCGCAGTCACTGACGTG
hsa_miR_4700_5p	178	-23.5	ACGCCACCTCTTCCTCAGTGCCCCAGC	TCTGGGGATGAGGACAGTGTGT
hsa_miR_4700_5p	127	-15	TTCTACTTCCTGTTCCAGA	TCTGGGGATGAGGACAGTGTGT
hsa_miR_4701_3p	178	-18.2	ACGCCACCTCTTCCTCAG	ATGGGTGATGGGTGTGGTGT
hsa_miR_4706	146	-31.1	AGGACAGCCCCTCATGCCTTCCCGCG	AGCGGGGAGGAAGTGGGCGCTGCTT
hsa_miR_4706	13	-26.2	CAGCAGCAAACACTGCCCCCGTG	AGCGGGGAGGAAGTGGGCGCTGCTT
hsa_miR_4706	340	-17.8	TCTGGGCCTCAGTTTTCTCATCTGTG	AGCGGGGAGGAAGTGGGCGCTGCTT
hsa_miR_4707_5p	473	-17.7	AAAGGGCTGGAAGAAGCAGGGGA	GCCCCGGCGCGGGCGGGTTCTGG
hsa_miR_4708_3p	35	-15	GACTTCCTGGTGCCATCCTTGGCT	AGCAAGGCGGCATCTCTCTGAT
hsa_miR_4713_3p	195	-15.4	GTGCCCCAGCCTGGCATCCTC	TGGGATCCAGACAGTGGGAGAA
hsa_miR_4717_3p	343	-21.9	GGGCCTCAGTTTTCTCATCTGT	ACACATGGGTGGCTGTGGCCT
hsa_miR_4721	181	-20.1	CCACCTCTTCCTCAGTGCCCCA	TGAGGGCTCCAGGTGACGGTGG
hsa_miR_4722_5p	201	-34.1	CAGCCTGGCATCCTCTTCCTGCA	GGCAGGAGGGCTGTGCCAGGTTG
hsa_miR_4723_3p	473	-15.3	AAAGGGCTGGAAGAAGCAGGGGA	CCCTCTCTGGCTCCTCCCCAAA
hsa_miR_4723_5p	280	-16.7	AGCTCCAGCTCTAACTCTGTCCCA	TGGGGGAGCCATGAGATAAGAGCA
hsa_miR_4723_5p	196	-15	TGCCCCAGCCTGGCATCCTCTT	TGGGGGAGCCATGAGATAAGAGCA
hsa_miR_4724_3p	131	-20.4	ACTTCCTGTTCCAGAAGGAC	GTACCTTCTGGTTCAGCTAGT
hsa_miR_4724_5p	326	-18.3	AGATGCTTAACTTCTCTGGGCCTCAGTT	AACTGAACCAGGAGTGAGCTTCG
hsa_miR_4725_3p	199	-19.6	CCCAGCCTGGCATCCTCTTCCTG	TGGGGAAGGCGTCAGTGTCGGG
hsa_miR_4725_3p	218	-19.2	CCTGCATTCAGCCTTCTTTT	TGGGGAAGGCGTCAGTGTCGGG
hsa_miR_4725_3p	174	-15	CCACACGCCACCTCTTCCTCA	TGGGGAAGGCGTCAGTGTCGGG
hsa_miR_4726_5p	328	-22.6	ATGCTTAACTTCTCTGGGCCT	AGGGCCAGAGGAGCCTGGAGTGG
hsa_miR_4728_5p	147	-20.1	GGACAGCCCCTCATGCCTTCCCG	TGGGAGGGGAGAGGCAGCAAGCA
hsa_miR_4728_5p	213	-16.2	CTCTTCCTGCATTCAGCCTTCTTT	TGGGAGGGGAGAGGCAGCAAGCA
hsa_miR_4728_5p	338	-15.8	TCTCTGGGCCTCAGTTTTCTCA	TGGGAGGGGAGAGGCAGCAAGCA
hsa_miR_4728_5p	121	-15	AGAATGTTCTACTTCCTGTTCCA	TGGGAGGGGAGAGGCAGCAAGCA
hsa_miR_4731_5p	187	-19.8	CTTCCTCAGTGCCCCAGCC	TGCTGGGGGCCACATGAGTGTG
hsa_miR_4731_5p	332	-18.2	TTAACTTCTCTGGGCCTCAGTT	TGCTGGGGGCCACATGAGTGTG
hsa_miR_4736	150	-17.7	CAGCCCCTCATGCCTTCCC	AGGCAGGTTATCTGGGCTG
hsa_miR_4736	279	-16.6	GAGCTCCAGCTCTAACTCTGTCC	AGGCAGGTTATCTGGGCTG
hsa_miR_4736	201	-15.9	CAGCCTGGCATCCTCTTCCT	AGGCAGGTTATCTGGGCTG
hsa_miR_4738_3p	337	-22.8	TTCTCTGGGCCTCAGTTTTC	TGAAACTGGAGCGCCTGGAGGA
hsa_miR_4739	209	-21	CATCCTCTTCCTGCATTCAGCCTTCTTT	AAGGGAGGAGGAGCGGAGGGGCCCT
hsa_miR_4740_3p	322	-16	GGCAAGATGCTTAACTTCTCTGGGC	GCCCGAGAGGATCCGTCCCTGC

hsa_miR_4741	134	-25.2	TCCTGTTCCAGAAGGACAGCCCC	CGGGCTGTCCGGAGGGGTCGGCT
hsa_miR_4741	194	-19.3	AGTGCCCCAGCCTGGCATCCTC	CGGGCTGTCCGGAGGGGTCGGCT
hsa_miR_4743_3p	422	-17.4	CAGGACAGAGGAAACAGAAA	TTTCTGTCTTTTCTGGTCCAG
hsa_miR_4745_5p	196	-17.9	TGCCCCAGCCTGGCATCCTCTTCC	TGAGTGGGGCTCCCGGGACGGCG
hsa_miR_4745_5p	113	-17.2	TGCCCTTCAGAATGTTCTACTTC	TGAGTGGGGCTCCCGGGACGGCG
hsa_miR_4746_3p	194	-17.8	AGTGCCCCAGCCTGGCATCCT	AGCGGTGCTCCTGCGGGCCGA
hsa_miR_4746_3p	8	-16.4	CTGGACAGCAGCAAACACTGCC	AGCGGTGCTCCTGCGGGCCGA
hsa_miR_4747_5p	198	-17.1	CCCCAGCCTGGCATCCTCTTCCT	AGGGAAGGAGGCTTGGTCTTAG
hsa_miR_4747_5p	175	-16.8	CACACGCCACCTCTTCCTC	AGGGAAGGAGGCTTGGTCTTAG
hsa_miR_4748	329	-20.9	TGCTTAACTTCTCTGGGCCTC	GAGGTTTGGGGAGGATTTGCT
hsa_miR_4748	147	-15.7	GGACAGCCCCTCATGCCTT	GAGGTTTGGGGAGGATTTGCT
hsa_miR_4750_5p	329	-18.2	TGCTTAACTTCTCTGGGCCTCAG	CTCGGGCGGAGGTGGTTGAGTG
hsa_miR_4750_5p	189	-17.2	TCCTCAGTGCCCCAGCCTGGC	CTCGGGCGGAGGTGGTTGAGTG
hsa_miR_4754	281	-20.4	GCTCCAGCTCTAACTCTGTCCCAA	ATGCGGACCTGGGTTAGCGGAGT
hsa_miR_4757_5p	476	-20.3	GGGCTGGAAGAAGCAGGGGACCT	AGGCCTCTGTGACGTCACGGTGT
hsa_miR_4758_5p	285	-20.7	CAGCTCTAACTCTGTCCCAATCAG	GTGAGTGGGAGCCGGTGGGGCTG
hsa_miR_4758_5p	136	-20	CTGTTCCAGAAGGACAGCCCCTCAT	GTGAGTGGGAGCCGGTGGGGCTG
hsa_miR_4761_3p	147	-20.2	GGACAGCCCCTCATGCCTTC	GAGGGCATGCGCACTTTGTCC
hsa_miR_4761_3p	98	-17.6	GAAGAACGTGTGACATGCCCTT	GAGGGCATGCGCACTTTGTCC
hsa_miR_4763_3p	36	-25.6	ACTTCCTGGTGCCATCCTTGGCT	AGGCAGGGGCTGGTGCTGGGCGG
hsa_miR_4763_3p	7	-23.6	CCTGGACAGCAGCAAACACTGCCC	AGGCAGGGGCTGGTGCTGGGCGG
hsa_miR_4763_3p	278	-23.5	TGAGCTCCAGCTCTAACTCTGTCC	AGGCAGGGGCTGGTGCTGGGCGG
hsa_miR_4764_5p	203	-15.1	GCCTGGCATCCTCTTCCTGCATTCA	TGGATGTGGAAGGAGTTATCT
hsa_miR_4767	10	-16.8	GGACAGCAGCAAACACTGCCCCCGTG	CGCGGGCGCTCCTGGCCGCCGCC
hsa_miR_4769_5p	109	-16.4	GACATGCCCTTCAGAATGTTCTACT	GGTGGGATGGAGAGAAGGTATGAG
hsa_miR_4769_5p	207	-16.2	GGCATCCTCTTCCTGCATTCAGCC	GGTGGGATGGAGAGAAGGTATGAG
hsa_miR_4776_5p	38	-27.9	TTCCTGGTGCCATCCTTGGCTCCAT	GTGGACCAGGATGGCAAGGGCT
hsa_miR_4776_5p	113	-15.3	TGCCCTTCAGAATGTTCTAC	GTGGACCAGGATGGCAAGGGCT
hsa_miR_4776_5p	125	-15.2	TGTTCTACTTCCTGTTCCAG	GTGGACCAGGATGGCAAGGGCT
hsa_miR_4783_3p	324	-15.7	CAAGATGCTTAACTTCTCTGGGC	CCCCGGTGTTGGGGCGCGTCTGC
hsa_miR_4784	200	-22	CCAGCCTGGCATCCTCTTCC	TGAGGAGATGCTGGGACTGA
hsa_miR_4785	40	-17.5	CCTGGTGCCATCCTTGGCTCC	AGAGTCGGCGACGCCGCCAGC
hsa_miR_4786_3p	190	-19	CCTCAGTGCCCCAGCCTGGCATCC	TGAAGCCAGCTCTGGTCTGGGC
hsa_miR_4786_3p	39	-17.6	TCCTGGTGCCATCCTTGGCTCCA	TGAAGCCAGCTCTGGTCTGGGC
hsa_miR_4787_5p	35	-20.8	GACTTCCTGGTGCCATCCTTGG	GCGGGGGTGGCGGCGGCATCCC
hsa_miR_4788	345	-16.1	GCCTCAGTTTTCTCATCTGTGA	TTACGGACCAGCTAAGGGAGGC
hsa_miR_483_5p	215	-15.1	CTTCCTGCATTCAGCCTTCTT	AAGACGGGAGGAAAGAAGGGAG
hsa_miR_490_5p	35	-15.8	GACTTCCTGGTGCCATCCTTGG	CCATGGATCTCCAGGTGGGT
hsa_miR_5001_3p	474	-21.4	AAGGGCTGGAAGAAGCAGGG	TTCTGCCTCTGTCCAGGTCCTT
hsa_miR_5001_5p	207	-22.7	GGCATCCTCTTCCTGCATTCAGCCTT	AGGGCTGGACTCAGCGGCGGAGCT
hsa_miR_5001_5p	137	-19.7	TGTTCCAGAAGGACAGCCCC	AGGGCTGGACTCAGCGGCGGAGCT
hsa_miR_5001_5p	267	-19.1	ACCAGGCAACGTGAGCTCCAGCTCT	AGGGCTGGACTCAGCGGCGGAGCT
hsa_miR_5001_5p	183	-18	ACCTCTTCCTCAGTGCCCCAGCCTG	AGGGCTGGACTCAGCGGCGGAGCT
hsa_miR_5001_5p	329	-15	TGCTTAACTTCTCTGGGCCTCAGTTTT	AGGGCTGGACTCAGCGGCGGAGCT
hsa_miR_5006_5p	191	-18.4	CTCAGTGCCCCAGCCTGGCAT	TTGCCAGGGCAGGAGGTGGAA
hsa_miR_5008_5p	328	-19.9	ATGCTTAACTTCTCTGGGCCTCA	TGAGGCCCTTGGGGCACAGTGG
hsa_miR_5010_5p	197	-19.8	GCCCCAGCCTGGCATCCTCTT	AGGGGGATGGCAGAGCAAAATT
hsa_miR_502_5p	474	-16.3	AAGGGCTGGAAGAAGCAGGGGA	ATCCTTGCTATCTGGGTGCTA
hsa_miR_503_3p	7	-26.6	CCTGGACAGCAGCAAACACTGCCCC	GGGGTATTGTTTCCGCTGCCAGG
hsa_miR_504_3p	194	-20.2	AGTGCCCCAGCCTGGCATCCT	GGGAGTGCAGGGCAGGGTTTC
hsa_miR_504_3p	43	-16.4	GGTGCCATCCTTGGCTCCA	GGGAGTGCAGGGCAGGGTTTC
hsa_miR_504_3p	144	-15.5	GAAGGACAGCCCCTCATGCCT	GGGAGTGCAGGGCAGGGTTTC
hsa_miR_505_5p	124	-16.9	ATGTTCTACTTCCTGTTCCA	GGGAGCCAGGAAGTATTGATGT
hsa_miR_5087	475	-16.9	AGGGCTGGAAGAAGCAGGGGACCT	GGGTTTGTAGCTTTGCTGGCATG
hsa_miR_5088_5p	287	-18.5	GCTCTAACTCTGTCCCAATCAGCTGTG	CAGGGCTCAGGGATTGGATGGAGG
hsa_miR_5089_5p	111	-17.2	CATGCCCTTCAGAATGTTCTAC	GTGGGATTTCTGAGTAGCATC
hsa_miR_5089_5p	149	-15.2	ACAGCCCCTCATGCCTTCCCGC	GTGGGATTTCTGAGTAGCATC
hsa_miR_5090	202	-16.4	AGCCTGGCATCCTCTTCCTGC	CCGGGGCAGATTGGTGTAGGGTG
hsa_miR_5093	335	-18.5	ACTTCTCTGGGCCTCAGTTTTCT	AGGAAATGAGGCTGGCTAGGAGC
hsa_miR_510_5p	174	-16.8	CCACACGCCACCTCTTCCTCAGTG	TACTCAGGAGAGTGGCAATCAC
hsa_miR_512_3p	188	-16.6	TTCCTCAGTGCCCCAGCCTG	AAGTGCTGTCATAGCTGAGGTC
hsa_miR_515_3p	327	-15.49	GATGCTTAACTTCTCTGGGCCTC	GAGTGCCTTCTTTTGGAGCGTT
hsa_miR_5187_5p	283	-17.3	TCCAGCTCTAACTCTGTCCCA	TGGGATGAGGGATTGAAGTGGA
hsa_miR_5189_5p	123	-20.2	AATGTTCTACTTCCTGTTCCAGA	TCTGGGCACAGGCGGATGGACAGG
hsa_miR_5189_5p	181	-18.3	CCACCTCTTCCTCAGTGCCCCAGC	TCTGGGCACAGGCGGATGGACAGG
hsa_miR_518c_5p	470	-16.84	CAGAAAGGGCTGGAAGAAGCAGGGG	TCTCTGGAGGGAAGCACTTTCTG
hsa_miR_5191	220	-15.8	TGCATTCAGCCTTCTTTTCCG	AGGATAGGAAGAATGAAGTGCT
hsa_miR_5192	199	-23	CCCAGCCTGGCATCCTCTTCCT	AGGAGAGTGGATTCCAGGTGGT
hsa_miR_5192	335	-15.1	ACTTCTCTGGGCCTCAGTTTTCT	AGGAGAGTGGATTCCAGGTGGT
hsa_miR_5194	192	-16.7	TCAGTGCCCCAGCCTGGCATCCTCT	TGAGGGGTTTGGAATGGGATGG
hsa_miR_5194	136	-16.4	CTGTTCCAGAAGGACAGCCCCTCA	TGAGGGGTTTGGAATGGGATGG
hsa_miR_5196_5p	199	-30.4	CCCAGCCTGGCATCCTCTTCCTG	AGGGAAGGGGACGAGGGTTGGG
hsa_miR_5196_5p	283	-22.2	TCCAGCTCTAACTCTGTCCCA	AGGGAAGGGGACGAGGGTTGGG
hsa_miR_5196_5p	148	-21.7	GACAGCCCCTCATGCCTTCCCG	AGGGAAGGGGACGAGGGTTGGG
hsa_miR_541_3p	138	-16.3	GTTCCAGAAGGACAGCCCCTCA	TGGTGGGCACAGAATCTGGACT
hsa_miR_548au_3p	41	-16.9	CTGGTGCCATCCTTGGCTCCA	TGGCAGTTACTTTTGCACCAG
hsa_miR_548au_3p	8	-16	CTGGACAGCAGCAAACACTGCCC	TGGCAGTTACTTTTGCACCAG
hsa_miR_550a_5p	328	-17.1	ATGCTTAACTTCTCTGGGCCT	AGTGCCTGAGGGAGTAAGAGCCC

hsa_miR_5572	184	-21.7	CCTCTTCCTCAGTGCCCCAGC	GTTGGGGTGCAGGGGTCTGCT
hsa_miR_5572	286	-17.3	AGCTCTAACTCTGTCCCAAT	GTTGGGGTGCAGGGGTCTGCT
hsa_miR_5572	332	-17.3	TTAACTTCTCTGGGCCTCAGT	GTTGGGGTGCAGGGGTCTGCT
hsa_miR_5572	210	-15.2	ATCCTCTTCCTGCATTCAGC	GTTGGGGTGCAGGGGTCTGCT
hsa_miR_5586_3p	278	-16.8	TGAGCTCCAGCTCTAACTCTG	CAGAGTGACAAGCTGGTTAAAG
hsa_miR_5587_3p	327	-16.2	GATGCTTAACTTCTCTGGGC	GCCCCGGGCAGTGTGATCATC
hsa_miR_5591_5p	195	-20.4	GTGCCCCAGCCTGGCATCCTC	TGGGAGCTAAGCTATGGGTAT
hsa_miR_5683	344	-15.6	GGCCTCAGTTTTCTCATCTGTG	TACAGATGCAGATTCTCTGACTTC
hsa_miR_5687	109	-16.8	GACATGCCCTTCAGAATGTTCTAC	TTAGAACGTTTTAGGGTCAAAT
hsa_miR_5694	341	-15.2	CTGGGCCTCAGTTTTCTCATCTG	CAGATCATGGGACTGTCTCAG
hsa_miR_5698	181	-18.1	CCACCTCTTCCTCAGTGCCCCA	TGGGGGAGTGCAGTGATTGTGG
hsa_miR_5698	141	-15.4	CCAGAAGGACAGCCCCTCA	TGGGGGAGTGCAGTGATTGTGG
hsa_miR_572	475	-16.6	AGGGCTGGAAGAAGCAGGGGAC	GTCCGCTCGGCGGTGGCCCA
hsa_miR_5739	345	-18.6	GCCTCAGTTTTCTCATCTGT	GCGGAGAGAGAATGGGGAGC
hsa_miR_575	37	-21.3	CTTCCTGGTGCCATCCTTGGCTC	GAGCCAGTTGGACAGGAGC
hsa_miR_5787	186	-36.2	TCTTCCTCAGTGCCCCAGCCT	GGGCTGGGGCGCGGGGAGGT
hsa_miR_5787	335	-29	ACTTCTCTGGGCCTCAGTTT	GGGCTGGGGCGCGGGGAGGT
hsa_miR_5787	213	-25.1	CTCTTCCTGCATTCAGCCT	GGGCTGGGGCGCGGGGAGGT
hsa_miR_5787	271	-18.4	GGCAACGTGAGCTCCAGCTC	GGGCTGGGGCGCGGGGAGGT
hsa_miR_584_5p	294	-16.6	CTCTGTCCCAATCAGCTGTGT	TTATGGTTTGCCTGGGACTGAG
hsa_miR_597_3p	476	-16.9	GGGCTGGAAGAAGCAGGGGACCT	TGGTTCTCTTGTGGCTCAAGCGT
hsa_miR_598_3p	442	-15.8	GTACATGACCGATGATGTC	TACGTCATCGTTGTCATCGTCA
hsa_miR_598_5p	287	-15.4	GCTCTAACTCTGTCCCAATCAGCTGT	GCGGTGATCCCGATGGTGTGAGC
hsa_miR_602	279	-16.1	GAGCTCCAGCTCTAACTCTGTC	GACACGGGCGACAGCTGCGGCCC
hsa_miR_604	188	-17.7	TTCCTCAGTGCCCCAGCCT	AGGCTGCGGAATTCAGGAC
hsa_miR_6069	185	-18.9	CTCTTCCTCAGTGCCCCAGCCT	GGGCTAGGGCCTGCTGCCCCC
hsa_miR_6069	40	-16.8	CCTGGTGCCATCCTTGGCTC	GGGCTAGGGCCTGCTGCCCCC
hsa_miR_6077	199	-17.3	CCCAGCCTGGCATCCTCTTCCT	GGGAAGAGCTGTACGGCCTTC
hsa_miR_6077	341	-16.9	CTGGGCCTCAGTTTTCTC	GGGAAGAGCTGTACGGCCTTC
hsa_miR_6077	176	-16	ACACGCCACCTCTTCCT	GGGAAGAGCTGTACGGCCTTC
hsa_miR_6079	38	-15.1	TTCCTGGTGCCATCCTTGGCTCCAT	TTGGAAGCTTGGACCAACTAGCTG
hsa_miR_608	30	-21.9	CCCGTGACTTCCTGGTGCCATCCTT	AGGGGTGGTGTTGGGACAGCTCCGT
hsa_miR_608	190	-17.5	CCTCAGTGCCCCAGCCTGGCATCCTC	AGGGGTGGTGTTGGGACAGCTCCGT
hsa_miR_608	145	-16.6	AAGGACAGCCCCTCATGCCTTCCCG	AGGGGTGGTGTTGGGACAGCTCCGT
hsa_miR_608	194	-15.5	AGTGCCCCAGCCTGGCATCCTCTT	AGGGGTGGTGTTGGGACAGCTCCGT
hsa_miR_6081	200	-16.3	CCAGCCTGGCATCCTCTTCCT	AGGAGCAGTGCCGGCCAAGGCGCC
hsa_miR_6086	213	-19.6	CTCTTCCTGCATTCAGCCTTC	GGAGGTTGGGAAGGGCAGAG
hsa_miR_6086	148	-16.7	GACAGCCCCTCATGCCTTC	GGAGGTTGGGAAGGGCAGAG
hsa_miR_6087	148	-22.3	GACAGCCCCTCATGCCTTC	TGAGGCGGGGGGGCGAGC
hsa_miR_6087	176	-21.8	ACACGCCACCTCTTCCTCA	TGAGGCGGGGGGGCGAGC
hsa_miR_6087	208	-17.6	GCATCCTCTTCCTGCATTCA	TGAGGCGGGGGGGCGAGC
hsa_miR_6087	42	-17.2	TGGTGCCATCCTTGGCTCC	TGAGGCGGGGGGGCGAGC
hsa_miR_6087	287	-15.4	GCTCTAACTCTGTCCCA	TGAGGCGGGGGGGCGAGC
hsa_miR_6088	196	-20.4	TGCCCCAGCCTGGCATCCTCT	AGAGATGAAGCGGGGGGGCG
hsa_miR_6088	280	-15.5	AGCTCCAGCTCTAACTCT	AGAGATGAAGCGGGGGGGCG
hsa_miR_6089	190	-31.5	CCTCAGTGCCCCAGCCTGGCATCC	GGAGGCCGGGGTGGGGCGGGGCGG
hsa_miR_6089	328	-23.5	ATGCTTAACTTCTCTGGGCCTCA	GGAGGCCGGGGTGGGGCGGGGCGG
hsa_miR_6089	209	-22.4	CATCCTCTTCCTGCATTCAGCCTTC	GGAGGCCGGGGTGGGGCGGGGCGG
hsa_miR_6090	180	-22.9	GCCACCTCTTCCTCAGTGCCCC	GGGGAGCGAGGGGCGGGGC
hsa_miR_6090	203	-22.9	GCCTGGCATCCTCTTCCTG	GGGGAGCGAGGGGCGGGGC
hsa_miR_6090	147	-22.5	GGACAGCCCCTCATGCCTTCCC	GGGGAGCGAGGGGCGGGGC
hsa_miR_610	39	-21	TCCTGGTGCCATCCTTGGCTCC	TGAGCTAAATGTGTGCTGGGA
hsa_miR_612	325	-17.7	AAGATGCTTAACTTCTCTGGGCCTCAG	GCTGGGCAGGGCTTCTGAGCTCCTT
hsa_miR_612	121	-15.2	AGAATGTTCTACTTCCTGTTCCAGA	GCTGGGCAGGGCTTCTGAGCTCCTT
hsa_miR_6124	124	-15.9	ATGTTCTACTTCCTGTTCCA	GGGAAAAGGAAGGGGGAGGA
hsa_miR_6125	149	-23.7	ACAGCCCCTCATGCCTTCCCGC	GCGGAAGGCGGAGCGGCGGA
hsa_miR_6125	199	-19.6	CCCAGCCTGGCATCCTCTTCCTGC	GCGGAAGGCGGAGCGGCGGA
hsa_miR_6125	283	-15.6	TCCAGCTCTAACTCTGT	GCGGAAGGCGGAGCGGCGGA
hsa_miR_6126	335	-19.9	ACTTCTCTGGGCCTCAG	GTGAAGGCCCGGCGGAGA
hsa_miR_6127	177	-20.9	CACGCCACCTCTTCCTCA	TGAGGGAGTGGGTGGGAGG
hsa_miR_6127	204	-19.3	CCTGGCATCCTCTTCCTGCA	TGAGGGAGTGGGTGGGAGG
hsa_miR_6127	150	-17.1	CAGCCCCTCATGCCTTCC	TGAGGGAGTGGGTGGGAGG
hsa_miR_6127	281	-17	GCTCCAGCTCTAACTCTGTCC	TGAGGGAGTGGGTGGGAGG
hsa_miR_6129	175	-18.4	CACACGCCACCTCTTCCTCA	TGAGGGAGTTGGGTGTATA
hsa_miR_6129	205	-15.5	CTGGCATCCTCTTCCTGCA	TGAGGGAGTTGGGTGTATA
hsa_miR_6131	185	-15.9	CTCTTCCTCAGTGCCCCAGCC	GGCTGGTCAGATGGGAGTG
hsa_miR_6132	191	-23.8	CTCAGTGCCCCAGCCTGGCA	AGCAGGGCTGGGGATTGCA
hsa_miR_6132	207	-18.3	GGCATCCTCTTCCTGCA	AGCAGGGCTGGGGATTGCA
hsa_miR_6133	151	-16.1	AGCCCCTCATGCCTTCCCG	TGAGGGAGGAGGTTGGGTA
hsa_miR_6133	205	-15.8	CTGGCATCCTCTTCCTGCA	TGAGGGAGGAGGTTGGGTA
hsa_miR_6134	218	-15.5	CCTGCATTCAGCCTTCTTT	TGAGGTGGTAGGATGTAGA
hsa_miR_615_5p	138	-15	GTTCCAGAAGGACAGCCCCT	GGGGGTCCCCGGTGCTCGGATC
hsa_miR_619_5p	183	-15.3	ACCTCTTCCTCAGTGCCCCAGC	GCTGGGATTACAGGCATGAGCC
hsa_miR_636	420	-15.6	GCAGGACAGAGGAAACAGAAAGTAC	TGTGCTTGCTCGTCCCGCCCGCA
hsa_miR_637	328	-16.2	ATGCTTAACTTCTCTGGGCCTCAGT	ACTGGGGGCTTTCGGGCTCTGCGT
hsa_miR_639	473	-15.7	AAAGGGCTGGAAGAAGCAGGGGA	ATCGCTGCGGTTGCGAGCGCTGT
hsa_miR_645	192	-21.5	TCAGTGCCCCAGCCTGGC	TCTAGGCTGGTACTGCTGA
hsa_miR_6499_3p	7	-21.7	CCTGGACAGCAGCAAACACTGCC	AGCAGTGTTTGTTTTGCCCACA

hsa_miR_6499_5p	190	-17.6	CCTCAGTGCCCCAGCCTGG	TCGGGCGCAAGAGCACTGCAGT
hsa_miR_6505_5p	123	-15	AATGTTCTACTTCCTGTTCCAG	TTGGAATAGGGGATATCTCAGC
hsa_miR_6509_5p	189	-15.5	TCCTCAGTGCCCCAGCCTGGC	ATTAGGTAGTGGCAGTGGAAC
hsa_miR_6510_5p	334	-15.2	AACTTCTCTGGGCCTCAGTTT	CAGCAGGGGAGAGAGAGGAGTC
hsa_miR_6512_3p	462	-19.4	TTCGACCACAGAAAGGGCTGGAA	TTCCAGCCCTTCTAATGGTAGG
hsa_miR_6515_5p	102	-15.7	AACGTGTGACATGCCCTTCAG	TTGGAGGGTGTGGAAGACATC
hsa_miR_652_3p	45	-17	TGCCATCCTTGGCTCCATT	AATGGCGCCACTAGGGTTGTG
hsa_miR_656_5p	480	-17	TGGAAGAAGCAGGGGACCT	AGGTTGCCTGTGAGGTGTTCA
hsa_miR_657	478	-15.9	GCTGGAAGAAGCAGGGGACCTACT	GGCAGGTTCTCACCCTCTCTAGG
hsa_miR_658	140	-22.1	TCCAGAAGGACAGCCCCTCATGCC	GGCGGAGGGAAGTAGGTCCGTTGGT
hsa_miR_661	188	-17.2	TTCCTCAGTGCCCCAGCCTGGCA	TGCCTGGGTCTCTGGCCTGCGCGT
hsa_miR_663a	184	-25.2	CCTCTTCCTCAGTGCCCCAGCCT	AGGCGGGGCGCCGCGGGACCGC
hsa_miR_663a	334	-15.9	AACTTCTCTGGGCCTCAGTTT	AGGCGGGGCGCCGCGGGACCGC
hsa_miR_663a	213	-15	CTCTTCCTGCATTCAGCCTTCTT	AGGCGGGGCGCCGCGGGACCGC
hsa_miR_663b	37	-16.5	CTTCCTGGTGCCATCCTTGGCTCC	GGTGGCCCGGCCGTGCCTGAGG
hsa_miR_664b_3p	473	-17.6	AAAGGGCTGGAAGAAGCAGGGGAC	TTCATTTGCCTCCCAGCCTACA
hsa_miR_664b_5p	38	-18.8	TTCCTGGTGCCATCCTTGGCTCCA	TGGGCTAAGGGAGATGATTGGGTA
hsa_miR_671_5p	191	-26	CTCAGTGCCCCAGCCTGGCATCCT	AGGAAGCCCTGGAGGGGCTGGAG
hsa_miR_671_5p	147	-25	GGACAGCCCCTCATGCCTTCCC	AGGAAGCCCTGGAGGGGCTGGAG
hsa_miR_671_5p	38	-22.4	TTCCTGGTGCCATCCTTGGCTCCA	AGGAAGCCCTGGAGGGGCTGGAG
hsa_miR_671_5p	117	-16.7	CTTCAGAATGTTCTACTTCCT	AGGAAGCCCTGGAGGGGCTGGAG
hsa_miR_671_5p	216	-15.6	TTCCTGCATTCAGCCTTCTT	AGGAAGCCCTGGAGGGGCTGGAG
hsa_miR_6716_5p	202	-18.4	AGCCTGGCATCCTCTTCCTG	TGGGAATGGGGGTAAGGGCC
hsa_miR_6716_5p	151	-17.4	AGCCCCTCATGCCTTCCCG	TGGGAATGGGGGTAAGGGCC
hsa_miR_6716_5p	337	-15.7	TTCTCTGGGCCTCAGTTTTCTCA	TGGGAATGGGGGTAAGGGCC
hsa_miR_6717_5p	144	-18.6	GAAGGACAGCCCCTCATGCCT	AGGCGATGTGGGGATGTAGAGA
hsa_miR_6720_3p	180	-15	GCCACCTCTTCCTCAGTGCC	CGCGCCTGCAGGAACTGGTAGA
hsa_miR_6721_5p	188	-23.7	TTCCTCAGTGCCCCAGCCTG	TGGGCAGGGGCTTATTGTAGGAG
hsa_miR_6721_5p	282	-18.8	CTCCAGCTCTAACTCTGTCCCA	TGGGCAGGGGCTTATTGTAGGAG
hsa_miR_6721_5p	6	-15.7	ACCTGGACAGCAGCAAACACTGCCCC	TGGGCAGGGGCTTATTGTAGGAG
hsa_miR_6722_3p	204	-26.1	CCTGGCATCCTCTTCCTGCA	TGCAGGGGTCGGGTGGGCCAGG
hsa_miR_6722_3p	184	-17	CCTCTTCCTCAGTGCCCCAGCC	TGCAGGGGTCGGGTGGGCCAGG
hsa_miR_6722_3p	276	-16.2	CGTGAGCTCCAGCTCTAACTCTGTC	TGCAGGGGTCGGGTGGGCCAGG
hsa_miR_6724_5p	328	-15.7	ATGCTTAACTTCTCTGGGCCTCAG	CTGGGCCCGCGGCGGGCGTGGGG
hsa_miR_6726_5p	203	-18	GCCTGGCATCCTCTTCCTG	CGGGAGCTGGGGTCTGCAGGT
hsa_miR_6727_5p	179	-20.9	CGCCACCTCTTCCTCAGTGCCCCAG	CTCGGGGCAGGCGGCTGGGAGCG
hsa_miR_6727_5p	35	-19.3	GACTTCCTGGTGCCATCCTTGGC	CTCGGGGCAGGCGGCTGGGAGCG
hsa_miR_6727_5p	125	-18.9	TGTTCTACTTCCTGTTCCAGAA	CTCGGGGCAGGCGGCTGGGAGCG
hsa_miR_6728_3p	473	-16.9	AAAGGGCTGGAAGAAGCAGGGG	TCTCTGCTCTGCTCTCCCCAG
hsa_miR_6728_5p	284	-17.8	CCAGCTCTAACTCTGTCCCAA	TTGGGATGGTAGGACCAGAGGGG
hsa_miR_6729_5p	197	-21.8	GCCCCAGCCTGGCATCCTCTTCCT	TGGGCGAGGGCGGCTGAGCGGC
hsa_miR_6729_5p	218	-19.6	CCTGCATTCAGCCTTCTTTTCCG	TGGGCGAGGGCGGCTGAGCGGC
hsa_miR_6729_5p	208	-16.7	GCATCCTCTTCCTGCATTCAGCCTT	TGGGCGAGGGCGGCTGAGCGGC
hsa_miR_6731_5p	217	-16.8	TCCTGCATTCAGCCTTCTTTTCCG	TGGGAGAGCAGGGTATTGTGGA
hsa_miR_6731_5p	337	-15.2	TTCTCTGGGCCTCAGTTTTCTCA	TGGGAGAGCAGGGTATTGTGGA
hsa_miR_6732_5p	198	-29.9	CCCCAGCCTGGCATCCTCTT	TAGGGGGTGGCAGGCTGGCC
hsa_miR_6732_5p	139	-17.8	TTCCAGAAGGACAGCCCCTC	TAGGGGGTGGCAGGCTGGCC
hsa_miR_6732_5p	117	-16.2	CTTCAGAATGTTCTACTTCCTG	TAGGGGGTGGCAGGCTGGCC
hsa_miR_6733_5p	337	-15.5	TTCTCTGGGCCTCAGTTTTCTCA	TGGGAAAGACAAACTCAGAGTT
hsa_miR_6734_5p	281	-20	GCTCCAGCTCTAACTCTGTCCCAA	TTGAGGGGAGAATGAGGTGGAGA
hsa_miR_6735_3p	142	-15	CAGAAGGACAGCCCCTCATGCCT	AGGCCTGTGGCTCCTCCCTCAG
hsa_miR_6735_5p	141	-19.19	CCAGAAGGACAGCCCCTCATGCCTTC	CAGGGCAGAGGGCACAGGAATCTGA
hsa_miR_6737_3p	474	-17.6	AAGGGCTGGAAGAAGCAGGGG	TCTGTGCTTCACCCCTACCCAG
hsa_miR_6737_5p	282	-19.7	CTCCAGCTCTAACTCTGTCCCAA	TTGGGGTGGTCGGCCCTGGAG
hsa_miR_6738_5p	198	-16.3	CCCCAGCCTGGCATCCTCT	CGAGGGGTAGAAGAGCACAGGGG
hsa_miR_6738_5p	212	-15.5	CCTCTTCCTGCATTCAGCCTTCT	CGAGGGGTAGAAGAGCACAGGGG
hsa_miR_6740_5p	184	-17.6	CCTCTTCCTCAGTGCCCCAGCC	AGTTTGGGATGGAGAGAGGAGA
hsa_miR_6741_5p	204	-20.3	CCTGGCATCCTCTTCCTGCATTCAG	GTGGGTGCTGGTGGGAGCCGTG
hsa_miR_6742_5p	148	-17.5	GACAGCCCCTCATGCCTTCCCGCG	AGTGGGGTGGGACCCAGCTGTT
hsa_miR_6744_3p	476	-16.4	GGGCTGGAAGAAGCAGGGGACCT	GGGCCTCTCTTGTCATCCTGCAG
hsa_miR_6744_5p	343	-18.6	GGGCCTCAGTTTTCTCATCTG	TGGATGACAGTGGAGGCCT
hsa_miR_6744_5p	194	-18	AGTGCCCCAGCCTGGCATCCT	TGGATGACAGTGGAGGCCT
hsa_miR_6746_5p	326	-15.5	AGATGCTTAACTTCTCTGGG	CCGGGAGAAGGAGGTGGCCTGG
hsa_miR_6747_5p	191	-15.2	CTCAGTGCCCCAGCCTGGCATCCTC	AGGGGTGTGGAAAGAGGCAGAACA
hsa_miR_6749_5p	186	-30	TCTTCCTCAGTGCCCCAGCCTGG	TCGGGCCTGGGGTTGGGGGAGC
hsa_miR_675_5p	200	-17.1	CCAGCCTGGCATCCTCTTCCTGCATTC	TGGTGCGGAGAGGGCCCACAGTG
hsa_miR_6751_5p	40	-24.1	CCTGGTGCCATCCTTGGCTCCAT	TTGGGGGTGAGGTTGGTGTCTGG
hsa_miR_6751_5p	174	-22.6	CCACACGCCACCTCTTCCTCAG	TTGGGGGTGAGGTTGGTGTCTGG
hsa_miR_6751_5p	284	-19.3	CCAGCTCTAACTCTGTCCCAA	TTGGGGGTGAGGTTGGTGTCTGG
hsa_miR_6752_5p	198	-25.6	CCCCAGCCTGGCATCCTCTTCCT	GGGGGGTGTGGAGCCAGGGGGC
hsa_miR_6752_5p	214	-19.1	TCTTCCTGCATTCAGCCTTCT	GGGGGGTGTGGAGCCAGGGGGC
hsa_miR_6753_3p	138	-15.5	GTTCCAGAAGGACAGCCC	TGGTCTGTCTCTGCCCTGGCAC
hsa_miR_6754_5p	36	-21	ACTTCCTGGTGCCATCCTTGG	CCAGGGAGGCTGGTTTGGAGGA
hsa_miR_6756_5p	183	-29.1	ACCTCTTCCTCAGTGCCCCAGCCT	AGGGTGGGGCTGGAGGTGGGGCT
hsa_miR_6756_5p	207	-24	GGCATCCTCTTCCTGCATTCAGCCT	AGGGTGGGGCTGGAGGTGGGGCT
hsa_miR_6756_5p	202	-21	AGCCTGGCATCCTCTTCCTGCATTCA	AGGGTGGGGCTGGAGGTGGGGCT
hsa_miR_6756_5p	113	-18.1	TGCCCTTCAGAATGTTCTACTTC	AGGGTGGGGCTGGAGGTGGGGCT
hsa_miR_6757_5p	200	-24.8	CCAGCCTGGCATCCTCTTCCTG	TAGGGATGGGAGGCCAGGATGA

hsa_miR_6757_5p	36	-23.1	ACTTCCTGGTGCCATCCTTG	TAGGGATGGGAGGCCAGGATGA
hsa_miR_6760_5p	172	-19.8	GGCCACACGCCACCTCTTCCTC	CAGGGAGAAGGTGGAAGTGCAGA
hsa_miR_6760_5p	340	-19.3	TCTGGGCCTCAGTTTTCTCATCTG	CAGGGAGAAGGTGGAAGTGCAGA
hsa_miR_6760_5p	200	-18.9	CCAGCCTGGCATCCTCTTCCTG	CAGGGAGAAGGTGGAAGTGCAGA
hsa_miR_6762_5p	38	-20.1	TTCCTGGTGCCATCCTTGGCTCCA	CGGGGCCATGGAGCAGCCTGTGT
hsa_miR_6762_5p	328	-16.2	ATGCTTAACTTCTCTGGGCCTCA	CGGGGCCATGGAGCAGCCTGTGT
hsa_miR_6763_3p	474	-20.1	AAGGGCTGGAAGAAGCAGGGGAC	CTCCCCGGCCTCTGCCCCCAG
hsa_miR_6765_5p	184	-27.4	CCTCTTCCTCAGTGCCCCAGCCTGGC	GTGAGGCGGGGCCAGGAGGGTGTGT
hsa_miR_6769a_5p	342	-16.1	TGGGCCTCAGTTTTCTCATCT	AGGTGGGTATGGAGGAGCCCT
hsa_miR_6769a_5p	41	-15.5	CTGGTGCCATCCTTGGCTCCATTA	AGGTGGGTATGGAGGAGCCCT
hsa_miR_6769b_5p	208	-21.7	GCATCCTCTTCCTGCATTCAGCCT	TGGTGGGTGGGGAGGAGAAGTGC
hsa_miR_6771_5p	102	-18.5	AACGTGTGACATGCCCTTCAGAA	CTCGGGAGGGCATGGGCCAGGC
hsa_miR_6772_5p	190	-32.4	CCTCAGTGCCCCAGCCTGGCATCCT	TGGGTGTAGGCTGGAGCTGAGG
hsa_miR_6772_5p	204	-21.7	CCTGGCATCCTCTTCCTGCATTCA	TGGGTGTAGGCTGGAGCTGAGG
hsa_miR_6773_5p	327	-17.1	GATGCTTAACTTCTCTGGGCCTCAG	TTGGGCCCAGGAGTAAACAGGAT
hsa_miR_6774_5p	179	-17.5	CGCCACCTCTTCCTCAGTGCCCCAGC	ACTTGGGCAGGAGGGACCCTGTATG
hsa_miR_6774_5p	327	-15.8	GATGCTTAACTTCTCTGGGCCTCAGT	ACTTGGGCAGGAGGGACCCTGTATG
hsa_miR_6775_5p	121	-15.7	AGAATGTTCTACTTCCTGTTCCAGA	TCGGGGCATGGGGGAGGGAGGCTGG
hsa_miR_6776_5p	187	-22.1	CTTCCTCAGTGCCCCAGC	TCTGGGTGCAGTGGGGGTT
hsa_miR_6776_5p	209	-20.5	CATCCTCTTCCTGCATTCAGC	TCTGGGTGCAGTGGGGGTT
hsa_miR_6777_5p	14	-17.6	AGCAGCAAACACTGCCCCCGT	ACGGGGAGTCAGGCAGTGGTGGA
hsa_miR_6777_5p	115	-17.4	CCCTTCAGAATGTTCTACTTCCTGT	ACGGGGAGTCAGGCAGTGGTGGA
hsa_miR_6778_5p	152	-22.4	GCCCCTCATGCCTTCCCGCG	AGTGGGAGGACAGGAGGCAGGT
hsa_miR_6778_5p	340	-20	TCTGGGCCTCAGTTTTCTCATC	AGTGGGAGGACAGGAGGCAGGT
hsa_miR_6779_5p	340	-17.3	TCTGGGCCTCAGTTTTCTCAT	CTGGGAGGGGCTGGGTTTGGC
hsa_miR_6780a_3p	474	-17.4	AAGGGCTGGAAGAAGCAGGGGAC	CTCCTCTGTTTTCTTTCCTAG
hsa_miR_6780a_5p	281	-25.5	GCTCCAGCTCTAACTCTGTCCCAA	TTGGGAGGGAAGACAGCTGGAGA
hsa_miR_6780a_5p	146	-17.6	AGGACAGCCCCTCATGCCTTCCCGC	TTGGGAGGGAAGACAGCTGGAGA
hsa_miR_6780a_5p	138	-16.1	GTTCCAGAAGGACAGCCCCTCAT	TTGGGAGGGAAGACAGCTGGAGA
hsa_miR_6780b_5p	214	-24.5	TCTTCCTGCATTCAGCCTTCTTTT	TGGGGAAGGCTTGGCAGGGAAGA
hsa_miR_6781_5p	328	-19.4	ATGCTTAACTTCTCTGGGCCT	CGGGCCGGAGGTCAAGGGCGT
hsa_miR_6781_5p	285	-16.6	CAGCTCTAACTCTGTCCCA	CGGGCCGGAGGTCAAGGGCGT
hsa_miR_6781_5p	186	-16.3	TCTTCCTCAGTGCCCCAGCCTG	CGGGCCGGAGGTCAAGGGCGT
hsa_miR_6781_5p	124	-15.7	ATGTTCTACTTCCTGTTCC	CGGGCCGGAGGTCAAGGGCGT
hsa_miR_6782_5p	343	-19.9	GGGCCTCAGTTTTCTCATCTGTG	TAGGGGTGGGGGAATTCAGGGGTGT
hsa_miR_6782_5p	112	-19	ATGCCCTTCAGAATGTTCTACTTCCTG	TAGGGGTGGGGGAATTCAGGGGTGT
hsa_miR_6784_5p	185	-24	CTCTTCCTCAGTGCCCCAGC	GCCGGGGCTTTGGGTGAGGG
hsa_miR_6784_5p	192	-18.1	TCAGTGCCCCAGCCTGGC	GCCGGGGCTTTGGGTGAGGG
hsa_miR_6784_5p	15	-15.9	GCAGCAAACACTGCCCCCGT	GCCGGGGCTTTGGGTGAGGG
hsa_miR_6785_5p	216	-18.1	TTCCTGCATTCAGCCTTCTTT	TGGGAGGGCGTGGATGATGGTG
hsa_miR_6785_5p	282	-16	CTCCAGCTCTAACTCTGTCCCA	TGGGAGGGCGTGGATGATGGTG
hsa_miR_6785_5p	139	-16	TTCCAGAAGGACAGCCCCTCA	TGGGAGGGCGTGGATGATGGTG
hsa_miR_6787_5p	281	-26.6	GCTCCAGCTCTAACTCTGTCC	TGGCGGGGGTAGAGCTGGCTGC
hsa_miR_6787_5p	119	-20.1	TCAGAATGTTCTACTTCCTGTTC	TGGCGGGGGTAGAGCTGGCTGC
hsa_miR_6787_5p	200	-18.8	CCAGCCTGGCATCCTCTTCCT	TGGCGGGGGTAGAGCTGGCTGC
hsa_miR_6788_5p	175	-20.8	CACACGCCACCTCTTCCTCAG	CTGGGAGAAGAGTGGTGAAGA
hsa_miR_6791_5p	474	-15.6	AAGGGCTGGAAGAAGCAGGGG	CCCCTGGGGCTGGGCAGGCGGA
hsa_miR_6793_5p	181	-22.6	CCACCTCTTCCTCAGTGCCCCA	TGTGGGTTCTGGGTTGGGGTGA
hsa_miR_6795_3p	474	-25.1	AAGGGCTGGAAGAAGCAGGGGA	ACCCCTCGTTTCTTCCCCCAG
hsa_miR_6795_5p	200	-28.9	CCAGCCTGGCATCCTCTTCCTGCA	TGGGGGGACAGGATGAGAGGCTGT
hsa_miR_6795_5p	178	-21.8	ACGCCACCTCTTCCTCAGTGCCCCA	TGGGGGGACAGGATGAGAGGCTGT
hsa_miR_6796_5p	189	-20.6	TCCTCAGTGCCCCAGCCTGGCAT	TTGTGGGGTTGGAGAGCTGGCTG
hsa_miR_6796_5p	138	-20.1	GTTCCAGAAGGACAGCCCCTCAT	TTGTGGGGTTGGAGAGCTGGCTG
hsa_miR_6796_5p	13	-17.2	CAGCAGCAAACACTGCCCCCGT	TTGTGGGGTTGGAGAGCTGGCTG
hsa_miR_6797_5p	275	-17.9	ACGTGAGCTCCAGCTCTAACTCTGTCC[illegible]	AGGAGGGAAGGGGCTGAGAACAGGA
hsa_miR_6798_5p	199	-23.4	CCCAGCCTGGCATCCTCTTCCTGC	CCAGGGGGATGGGCGAGCTTGGG
hsa_miR_6798_5p	115	-17.3	CCCTTCAGAATGTTCTACTTCCTGT	CCAGGGGGATGGGCGAGCTTGGG
hsa_miR_6799_5p	149	-29.5	ACAGCCCCTCATGCCTTCCC	GGGGAGGTGTGCAGGGCTGG
hsa_miR_6799_5p	200	-27.6	CCAGCCTGGCATCCTCTTC	GGGGAGGTGTGCAGGGCTGG
hsa_miR_6799_5p	12	-19.9	ACAGCAGCAAACACTGCCCC	GGGGAGGTGTGCAGGGCTGG
hsa_miR_6799_5p	35	-15	GACTTCCTGGTGCCATCCTT	GGGGAGGTGTGCAGGGCTGG
hsa_miR_6801_3p	477	-22.2	GGCTGGAAGAAGCAGGGGA	ACCCCTGCCACTCACTGGCC
hsa_miR_6801_5p	278	-15.6	TGAGCTCCAGCTCTAACTCTGTCCC	TGGTCAGAGGCAGCAGGAAATGA
hsa_miR_6802_5p	186	-22.4	TCTTCCTCAGTGCCCCAGCCTGG	CTAGGTGGGGGGCTTGAAGC
hsa_miR_6802_5p	345	-16.3	GCCTCAGTTTTCTCATCTGT	CTAGGTGGGGGGCTTGAAGC
hsa_miR_6803_5p	11	-21.4	GACAGCAGCAAACACTGCCCCCGT	CTGGGGGTGGGGGGCTGGGCGT
hsa_miR_6803_5p	328	-20.8	ATGCTTAACTTCTCTGGGCCTCAG	CTGGGGGTGGGGGGCTGGGCGT
hsa_miR_6803_5p	208	-17.2	GCATCCTCTTCCTGCATTCAG	CTGGGGGTGGGGGGCTGGGCGT
hsa_miR_6804_5p	193	-19.1	CAGTGCCCCAGCCTGGCATCCTCT	TGAGGGTGTCAGCAGGTGACG
hsa_miR_6804_5p	35	-17.3	GACTTCCTGGTGCCATCCTTG	TGAGGGTGTCAGCAGGTGACG
hsa_miR_6804_5p	212	-15.9	CCTCTTCCTGCATTCAGCCTTCT	TGAGGGTGTCAGCAGGTGACG
hsa_miR_6805_3p	472	-19.2	GAAAGGGCTGGAAGAAGCAGGGGAC	TTGCTCTGCTCCCCCGCCCCCAG
hsa_miR_6805_5p	213	-15.1	CTCTTCCTGCATTCAGCCTTCTT	TAGGGGGCGGCTTGTGGAGTGT
hsa_miR_6806_5p	204	-21.1	CCTGGCATCCTCTTCCTGCA	TGTAGGCATGAGGCAGGGCCCAGG
hsa_miR_6806_5p	109	-15.4	GACATGCCCTTCAGAATGTTCTACT	TGTAGGCATGAGGCAGGGCCCAGG
hsa_miR_6808_5p	40	-19.8	CCTGGTGCCATCCTTGGCTC	CAGGCAGGGAGGTGGGACCATG
hsa_miR_6808_5p	184	-18.1	CCTCTTCCTCAGTGCCCCAGCCTG	CAGGCAGGGAGGTGGGACCATG
hsa_miR_6809_3p	474	-17.3	AAGGGCTGGAAGAAGCAGGGG	CTTCTCTTCTCTCCTTCCCAG

hsa_miR_6810_3p	474	-23.9	AAGGGCTGGAAGAAGCAGGGGA	TCCCCTGCTCCCTTGTTCCCCAG
hsa_miR_6810_5p	39	-20.6	TCCTGGTGCCATCCTTGGCTCCAT	ATGGGGACAGGGATCAGCATGGC
hsa_miR_6810_5p	180	-19.3	GCCACCTCTTCCTCAGTGCCCCAG	ATGGGGACAGGGATCAGCATGGC
hsa_miR_6810_5p	203	-16.5	GCCTGGCATCCTCTTCCTGCAT	ATGGGGACAGGGATCAGCATGGC
hsa_miR_6810_5p	15	-15.1	GCAGCAAACACTGCCCCCGT	ATGGGGACAGGGATCAGCATGGC
hsa_miR_6812_5p	180	-27.4	GCCACCTCTTCCTCAGTGCCCCAG	ATGGGGTGAGATGGGGAGGAGCAGC
hsa_miR_6812_5p	34	-19.1	GACTTCCTGGTGCCATCCTTGGCTCCA	ATGGGGTGAGATGGGGAGGAGCAGC
hsa_miR_6812_5p	147	-15.5	GGACAGCCCCTCATGCCTTCCCGC	ATGGGGTGAGATGGGGAGGAGCAGC
hsa_miR_6813_5p	200	-19.9	CCAGCCTGGCATCCTCTTCCTG	CAGGGGCTGGGGTTTCAGGTTCT
hsa_miR_6813_5p	137	-19.2	TGTTCCAGAAGGACAGCCCCTC	CAGGGGCTGGGGTTTCAGGTTCT
hsa_miR_6815_5p	32	-23.1	CGTGACTTCCTGGTGCCATCCTT	TAGGTGGCGCCGGAGGAGTCATT
hsa_miR_6816_5p	284	-18	CCAGCTCTAACTCTGTCCCA	TGGGGCGGGGCAGGTCCCTGC
hsa_miR_6816_5p	203	-15	GCCTGGCATCCTCTTCCTGCATTCA	TGGGGCGGGGCAGGTCCCTGC
hsa_miR_6819_5p	180	-20.5	GCCACCTCTTCCTCAGTGCCCCAG	TTGGGGTGGAGGGCCAAGGAGC
hsa_miR_6819_5p	330	-16.4	GCTTAACTTCTCTGGGCCTCAG	TTGGGGTGGAGGGCCAAGGAGC
hsa_miR_6820_5p	285	-17.3	CAGCTCTAACTCTGTCCCA	TGCGGCAGAGCTGGGGTCA
hsa_miR_6823_5p	276	-17.3	CGTGAGCTCCAGCTCTAACTCTGT	TCAGGGTTGGTAGGGGTTGCT
hsa_miR_6823_5p	34	-15.5	TGACTTCCTGGTGCCATCCTTGG	TCAGGGTTGGTAGGGGTTGCT
hsa_miR_6823_5p	294	-15.1	CTCTGTCCCAATCAGCTGTGT	TCAGGGTTGGTAGGGGTTGCT
hsa_miR_6824_3p	474	-18	AAGGGCTGGAAGAAGCAGGGG	TCTCTGGTCTTGCCACCCCCAG
hsa_miR_6824_5p	202	-25.4	AGCCTGGCATCCTCTTCCTGC	GTAGGGGAGGTTGGGCCAGGGA
hsa_miR_6825_5p	150	-19.5	CAGCCCCTCATGCCTTCCCG	TGGGGAGGTGTGGAGTCAGCAT
hsa_miR_6825_5p	172	-18	GGCCACACGCCACCTCTTCCTC	TGGGGAGGTGTGGAGTCAGCAT
hsa_miR_6826_3p	474	-20.3	AAGGGCTGGAAGAAGCAGGGGAC	CTCCCCTCTCTTTCCTGTTCAG
hsa_miR_6827_5p	147	-27.1	GGACAGCCCCTCATGCCTTCCC	TGGGAGCCATGAGGGTCTGTGC
hsa_miR_6827_5p	181	-18.2	CCACCTCTTCCTCAGTGCCCCA	TGGGAGCCATGAGGGTCTGTGC
hsa_miR_6829_5p	182	-23.2	CACCTCTTCCTCAGTGCCCC	TGGGCTGCTGAGAAGGGGCA
hsa_miR_6829_5p	196	-21.4	TGCCCCAGCCTGGCATCCTC	TGGGCTGCTGAGAAGGGGCA
hsa_miR_6829_5p	210	-18.2	ATCCTCTTCCTGCATTCAGCCTT	TGGGCTGCTGAGAAGGGGCA
hsa_miR_6830_5p	35	-18	GACTTCCTGGTGCCATCCTTGG	CCAAGGAAGGAGGCTGGACATC
hsa_miR_6831_5p	210	-26.7	ATCCTCTTCCTGCATTCAGCCTT	TAGGTAGAGTGTGAGGAGGAGGTC
hsa_miR_6831_5p	182	-25.8	CACCTCTTCCTCAGTGCCCCAGCCTG	TAGGTAGAGTGTGAGGAGGAGGTC
hsa_miR_6831_5p	196	-20.4	TGCCCCAGCCTGGCATCCTCTTCCTG	TAGGTAGAGTGTGAGGAGGAGGTC
hsa_miR_6831_5p	113	-18.2	TGCCCTTCAGAATGTTCTACTTC	TAGGTAGAGTGTGAGGAGGAGGTC
hsa_miR_6831_5p	336	-16.3	CTTCTCTGGGCCTCAGTTTTCTCATCTC	TAGGTAGAGTGTGAGGAGGAGGTC
hsa_miR_6832_3p	474	-15.1	AAGGGCTGGAAGAAGCAGGGG	ACCCTTTTTCTCTTTCCCAG
hsa_miR_6833_5p	126	-15.2	GTTCTACTTCCTGTTCCAGAA	GTGTGGAAGATGGGAGGAGAAA
hsa_miR_6833_5p	336	-15.1	CTTCTCTGGGCCTCAGTTTTCTCAT	GTGTGGAAGATGGGAGGAGAAA
hsa_miR_6834_5p	141	-20.3	CCAGAAGGACAGCCCCTCAT	GTGAGGGACTGGGATTTGTGG
hsa_miR_6835_5p	117	-22	CTTCAGAATGTTCTACTTCCT	AGGGGGTAGAAAGTGGCTGAAG
hsa_miR_6835_5p	10	-20.4	GGACAGCAGCAAACACTGCCCCCG	AGGGGGTAGAAAGTGGCTGAAG
hsa_miR_6835_5p	147	-16.7	GGACAGCCCCTCATGCCTTCC	AGGGGGTAGAAAGTGGCTGAAG
hsa_miR_6835_5p	198	-15.5	CCCCAGCCTGGCATCCTCTTCCT	AGGGGGTAGAAAGTGGCTGAAG
hsa_miR_6835_5p	215	-15.5	CTTCCTGCATTCAGCCTTCT	AGGGGGTAGAAAGTGGCTGAAG
hsa_miR_6836_5p	201	-18.8	CAGCCTGGCATCCTCTTCCTGCA	CGCAGGGCCCTGGCGCAGGCAT
hsa_miR_6839_3p	479	-21.6	CTGGAAGAAGCAGGGGACCTAC	TTGGGTTTTCTCTTCAATCCAG
hsa_miR_6840_3p	38	-16.9	TTCCTGGTGCCATCCTTGGC	GCCCAGGACTTTGTGCGGGGTG
hsa_miR_6841_3p	473	-16.8	AAAGGGCTGGAAGAAGCAGGGG	ACCTTGCATCTGCATCCCCAG
hsa_miR_6842_5p	135	-17.3	CCTGTTCCAGAAGGACAGCCCCTC	TGGGGGTGGTCTCTAGCCAAGG
hsa_miR_6845_5p	287	-19.5	GCTCTAACTCTGTCCCA	CGGGGCCAGAGCAGAGAGC
hsa_miR_6845_5p	330	-19.5	GCTTAACTTCTCTGGGCCTC	CGGGGCCAGAGCAGAGAGC
hsa_miR_6846_5p	212	-25.5	CCTCTTCCTGCATTCAGCCTTCT	TGGGGGCTGGATGGGGTAGAGT
hsa_miR_6846_5p	330	-21.4	GCTTAACTTCTCTGGGCCTCA	TGGGGGCTGGATGGGGTAGAGT
hsa_miR_6846_5p	38	-18.7	TTCCTGGTGCCATCCTTGGCTCCA	TGGGGGCTGGATGGGGTAGAGT
hsa_miR_6846_5p	10	-16.2	GGACAGCAGCAAACACTGCCCCCG	TGGGGGCTGGATGGGGTAGAGT
hsa_miR_6847_5p	330	-20.4	GCTTAACTTCTCTGGGCCTCAGT	ACAGAGGACAGTGGAGTGTGAGC
hsa_miR_6848_5p	39	-26.9	TCCTGGTGCCATCCTTGGCTCCA	TGGGGGCTGGGATGGGCCATGGT
hsa_miR_6848_5p	180	-23.5	GCCACCTCTTCCTCAGTGCCCCA	TGGGGGCTGGGATGGGCCATGGT
hsa_miR_6848_5p	9	-17.5	TGGACAGCAGCAAACACTGCCCCCG	TGGGGGCTGGGATGGGCCATGGT
hsa_miR_6848_5p	211	-16.3	TCCTCTTCCTGCATTCAGCCTTCT	TGGGGGCTGGGATGGGCCATGGT
hsa_miR_6849_5p	217	-15.6	TCCTGCATTCAGCCTTCTTTTC	GAGTGGATAGGGGAGTGTGTGGA
hsa_miR_6849_5p	203	-15.1	GCCTGGCATCCTCTTCCTGCATTC	GAGTGGATAGGGGAGTGTGTGGA
hsa_miR_6850_5p	151	-20.1	AGCCCCTCATGCCTTCCCGC	GTGCGGAACGCTGGCCGGGGCG
hsa_miR_6850_5p	201	-18.3	CAGCCTGGCATCCTCTTCCTGCAT	GTGCGGAACGCTGGCCGGGGCG
hsa_miR_6850_5p	338	-15.7	TCTCTGGGCCTCAGTTTTCTCAT	GTGCGGAACGCTGGCCGGGGCG
hsa_miR_6851_3p	479	-15.7	CTGGAAGAAGCAGGGGACCT	TGGCCCTTTGTACCCCTCCAG
hsa_miR_6851_5p	33	-21.6	GTGACTTCCTGGTGCCATCCTT	AGGAGGTGGTACTAGGGGCCAGC
hsa_miR_6851_5p	340	-18.3	TCTGGGCCTCAGTTTTCTCATCTGTG	AGGAGGTGGTACTAGGGGCCAGC
hsa_miR_6851_5p	277	-16.1	GTGAGCTCCAGCTCTAACTCTG	AGGAGGTGGTACTAGGGGCCAGC
hsa_miR_6851_5p	135	-15.3	CCTGTTCCAGAAGGACAGCCCCT	AGGAGGTGGTACTAGGGGCCAGC
hsa_miR_6852_3p	479	-16.6	CTGGAAGAAGCAGGGGACC	TGTCCTCTGTTCCTCAG
hsa_miR_6855_5p	180	-19	GCCACCTCTTCCTCAGTGCCCCAG	TTGGGGTTTGGGGTGCAGACATTGC
hsa_miR_6855_5p	38	-18.1	TTCCTGGTGCCATCCTTGGCTCCAT	TTGGGGTTTGGGGTGCAGACATTGC
hsa_miR_6856_5p	173	-17.8	GCCACACGCCACCTCTTCCTC	AAGAGAGGAGCAGTGGTGCTGTGG
hsa_miR_6857_5p	176	-17	ACACGCCACCTCTTCCTCAG	TTGGGGATTGGGTCAGGCCAGT
hsa_miR_6857_5p	181	-16.5	CCACCTCTTCCTCAGTGCCCCAG	TTGGGGATTGGGTCAGGCCAGT
hsa_miR_6858_5p	174	-21.7	CCACACGCCACCTCTTCCTCAG	GTGAGGAGGGGCTGGCAGGGAC
hsa_miR_6859_5p	336	-20.8	CTTCTCTGGGCCTCAGTTTTCTC	GAGAGGAACATGGGCTCAGGACA

hsa_miR_6859_5p	196	-19.9	TGCCCCAGCCTGGCATCCTCTT	GAGAGGAACATGGGCTCAGGACA
hsa_miR_6860	124	-16.8	ATGTTCTACTTCCTGTTCCAGA	ACTGGGCAGGGCTGTGGTGAGT
hsa_miR_6862_5p	180	-21.2	GCCACCTCTTCCTCAGTGCCCC	CGGGCATGCTGGGAGAGACTTT
hsa_miR_6862_5p	144	-18.4	GAAGGACAGCCCCTCATGCCTT	CGGGCATGCTGGGAGAGACTTT
hsa_miR_6864_5p	100	-16.9	AGAACGTGTGACATGCCCTTCAG	TTGAAGGGACAAGTCAGATATGCC
hsa_miR_6865_5p	30	-16.9	CCCGTGACTTCCTGGTGCCATCCTT	TAGGTGGCAGAGGAGGGACTTCA
hsa_miR_6867_5p	203	-15.8	GCCTGGCATCCTCTTCCTGCATT	TGTGTGTGTAGAGGAAGAAGGGA
hsa_miR_6870_5p	284	-18.8	CCAGCTCTAACTCTGTCCCA	TGGGGGAGATGGGGGTTGA
hsa_miR_6870_5p	177	-18.2	CACGCCACCTCTTCCTCA	TGGGGGAGATGGGGGTTGA
hsa_miR_6870_5p	200	-17.2	CCAGCCTGGCATCCTCTTCCT	TGGGGGAGATGGGGGTTGA
hsa_miR_6870_5p	15	-16.6	GCAGCAAACACTGCCCCCG	TGGGGGAGATGGGGGTTGA
hsa_miR_6870_5p	186	-16.5	TCTTCCTCAGTGCCCCA	TGGGGGAGATGGGGGTTGA
hsa_miR_6873_5p	341	-17.2	CTGGGCCTCAGTTTTCTCATCTG	CAGAGGGAATACAGAGGGCAAT
hsa_miR_6874_5p	39	-20.4	TCCTGGTGCCATCCTTGGCTCCAT	ATGGAGCTGGAACCAGATCAGGC
hsa_miR_6874_5p	270	-16.3	AGGCAACGTGAGCTCCAGCTCTAA	ATGGAGCTGGAACCAGATCAGGC
hsa_miR_6874_5p	122	-16.1	GAATGTTCTACTTCCTGTTCCAG	ATGGAGCTGGAACCAGATCAGGC
hsa_miR_6875_3p	467	-21	CCACAGAAAGGGCTGGAAGAAG	ATTCTTCCTGCCCTGGCTCCAT
hsa_miR_6877_5p	41	-20.7	CTGGTGCCATCCTTGGCTCC	AGGGCCGAAGGGTGGAAGCTGC
hsa_miR_6877_5p	211	-15.3	TCCTCTTCCTGCATTCAGCCTT	AGGGCCGAAGGGTGGAAGCTGC
hsa_miR_6878_5p	198	-17.8	CCCCAGCCTGGCATCCTCTTCCT	AGGGAGAAAGCTAGAAGCTGAAG
hsa_miR_6878_5p	340	-16.2	TCTGGGCCTCAGTTTTCTCATCT	AGGGAGAAAGCTAGAAGCTGAAG
hsa_miR_6880_5p	201	-23	CAGCCTGGCATCCTCTTCCTGCATTC	TGGTGGAGGAAGAGGGCAGCTC
hsa_miR_6880_5p	150	-19.9	CAGCCCCTCATGCCTTCCCG	TGGTGGAGGAAGAGGGCAGCTC
hsa_miR_6881_5p	183	-19.6	ACCTCTTCCTCAGTGCCCCA	TGGGGTAAGGATAGGAGGGTCA
hsa_miR_6883_5p	149	-21.1	ACAGCCCCTCATGCCTTCCCG	AGGGAGGGTGTGGTATGGATGT
hsa_miR_6883_5p	172	-20.9	GGCCACACGCCACCTCTTCCT	AGGGAGGGTGTGGTATGGATGT
hsa_miR_6883_5p	196	-18	TGCCCCAGCCTGGCATCCTCTTC	AGGGAGGGTGTGGTATGGATGT
hsa_miR_6883_5p	221	-17	GCATTCAGCCTTCTTTTCCG	AGGGAGGGTGTGGTATGGATGT
hsa_miR_6883_5p	281	-16.2	GCTCCAGCTCTAACTCTGTCCCA	AGGGAGGGTGTGGTATGGATGT
hsa_miR_6884_5p	147	-15.2	GGACAGCCCCTCATGCCTTC	AGAGGCTGAGAAGGTGATGTTG
hsa_miR_6885_3p	473	-17.8	AAAGGGCTGGAAGAAGCAGGG	CTTTGCTTCCTGCTCCCCTAG
hsa_miR_6887_3p	474	-17.4	AAGGGCTGGAAGAAGCAGGGGA	TCCCCTCCACTTTCCTCCTAG
hsa_miR_6891_5p	216	-17.3	TTCCTGCATTCAGCCTTCTTT	TAAGGAGGGGGATGAGGGG
hsa_miR_6891_5p	38	-16.9	TTCCTGGTGCCATCCTTG	TAAGGAGGGGGATGAGGGG
hsa_miR_6893_3p	475	-21.7	AGGGCTGGAAGAAGCAGGG	CCCTGCTGCCTTCACCTGCCAG
hsa_miR_6894_3p	475	-19	AGGGCTGGAAGAAGCAGGGGA	TTGCCTGCCCTCTTCCTCCAG
hsa_miR_6894_5p	194	-25.5	AGTGCCCCAGCCTGGCATCCTCTT	AGGAGGATGGAGAGCTGGGCCAGA
hsa_miR_6894_5p	199	-24.8	CCCAGCCTGGCATCCTCTTCCT	AGGAGGATGGAGAGCTGGGCCAGA
hsa_miR_6894_5p	144	-17.6	GAAGGACAGCCCCTCATGCCTTCC	AGGAGGATGGAGAGCTGGGCCAGA
hsa_miR_6894_5p	218	-17.5	CCTGCATTCAGCCTTCTTTTCCG	AGGAGGATGGAGAGCTGGGCCAGA
hsa_miR_7106_5p	283	-15.5	TCCAGCTCTAACTCTGTCCCA	TGGGAGGAGGGGATCTTGGG
hsa_miR_7106_5p	202	-15.4	AGCCTGGCATCCTCTTCCTG	TGGGAGGAGGGGATCTTGGG
hsa_miR_7107_5p	185	-30.1	CTCTTCCTCAGTGCCCCAGCCTGG	TCGGCCTGGGGAGGAGGAAGGG
hsa_miR_7109_5p	14	-20.7	AGCAGCAAACACTGCCCCCGT	CTGGGGGGAGGAGACCCTGCT
hsa_miR_711	331	-18.4	CTTAACTTCTCTGGGCCT	GGGACCCAGGGAGAGACGTAAG
hsa_miR_7110_5p	337	-16.3	TTCTCTGGGCCTCAGTTTTCTCA	TGGGGGTGTGGGGAGAGAGAG
hsa_miR_7111_3p	474	-19.1	AAGGGCTGGAAGAAGCAGGGGAC	ATCCTCTCTTCCCTCCTCCCAG
hsa_miR_7111_5p	200	-22.5	CCAGCCTGGCATCCTCTTCCT	TGGGGGAGGAAGGACAGGCCAT
hsa_miR_7111_5p	205	-21.4	CTGGCATCCTCTTCCTGCA	TGGGGGAGGAAGGACAGGCCAT
hsa_miR_7111_5p	124	-18	ATGTTCTACTTCCTGTTCCA	TGGGGGAGGAAGGACAGGCCAT
hsa_miR_7114_5p	177	-16.2	CACGCCACCTCTTCCTCAGT	TCTGTGGAGTGGGGTGCCTGT
hsa_miR_7155_5p	186	-17.1	TCTTCCTCAGTGCCCCAGC	TCTGGGGTCTTGGGCCATC
hsa_miR_7155_5p	334	-15.8	AACTTCTCTGGGCCTCAGT	TCTGGGGTCTTGGGCCATC
hsa_miR_7160_5p	333	-21.9	TAACTTCTCTGGGCCTCAGTT	TGCTGAGGTCCGGGCTGTGCC
hsa_miR_7160_5p	179	-16.7	CGCCACCTCTTCCTCAGTG	TGCTGAGGTCCGGGCTGTGCC
hsa_miR_7162_5p	476	-15	GGGCTGGAAGAAGCA	TGCTTCCTTTCTCAGCTG
hsa_miR_744_5p	189	-21.7	TCCTCAGTGCCCCAGCCTGGCA	TGCGGGGCTAGGGCTAACAGCA
hsa_miR_744_5p	280	-18.2	AGCTCCAGCTCTAACTCTGTC	TGCGGGGCTAGGGCTAACAGCA
hsa_miR_744_5p	200	-15.6	CCAGCCTGGCATCCTCTTCCTGCA	TGCGGGGCTAGGGCTAACAGCA
hsa_miR_758_5p	32	-16.3	CGTGACTTCCTGGTGCCATC	GATGGTTGACCAGAGAGCACAC
hsa_miR_762	184	-30	CCTCTTCCTCAGTGCCCCAGCCTG	GGGGCTGGGGCCGGGGCCGAGC
hsa_miR_762	281	-22.7	GCTCCAGCTCTAACTCTGTCCC	GGGGCTGGGGCCGGGGCCGAGC
hsa_miR_762	334	-21.3	AACTTCTCTGGGCCTCAGTTTT	GGGGCTGGGGCCGGGGCCGAGC
hsa_miR_762	212	-19.1	CCTCTTCCTGCATTCAGCCTT	GGGGCTGGGGCCGGGGCCGAGC
hsa_miR_766_5p	201	-18.7	CAGCCTGGCATCCTCTTCCT	AGGAGGAATTGGTGCTGGTCTT
hsa_miR_766_5p	171	-17.8	GGGCCACACGCCACCTCTTCCT	AGGAGGAATTGGTGCTGGTCTT
hsa_miR_7843_5p	144	-20.2	GAAGGACAGCCCCTCATGCCTTC	GAGGGCAGAGCCAGCTTCCTGA
hsa_miR_7847_3p	203	-22.3	GCCTGGCATCCTCTTCCTGCATT	CGTGGAGGACGAGGAGGAGGC
hsa_miR_7847_3p	46	-20.8	GCCATCCTTGGCTCCATT	CGTGGAGGACGAGGAGGAGGC
hsa_miR_7847_3p	152	-18.9	GCCCCTCATGCCTTCCCG	CGTGGAGGACGAGGAGGAGGC
hsa_miR_7847_3p	337	-18.2	TTCTCTGGGCCTCAGTTTTCTCATC	CGTGGAGGACGAGGAGGAGGC
hsa_miR_7850_5p	284	-19.6	CCAGCTCTAACTCTGTCCCAAT	GTTTGGACATAGTGTGGCTGG
hsa_miR_7974	206	-18.24	TGGCATCCTCTTCCTGCATTCAGCCT	AGGCTGTGATGCTCTCCTGAGCCC
hsa_miR_7974	140	-15.4	TCCAGAAGGACAGCCCCTCATGCCT	AGGCTGTGATGCTCTCCTGAGCCC
hsa_miR_8052	281	-20.2	GCTCCAGCTCTAACTCTGTCCCA	CGGGACTGTAGAGGGCATGAGC
hsa_miR_8059	180	-19	GCCACCTCTTCCTCAGTGCCCC	GGGGAACTGTAGATGAAAAGGC
hsa_miR_8069	143	-22.5	AGAAGGACAGCCCCTCATGCCTTCC	GGATGGTTGGGGGCGGTCGGCGT
hsa_miR_8069	279	-16.4	GAGCTCCAGCTCTAACTCTGTCC	GGATGGTTGGGGGCGGTCGGCGT

hsa_miR_8069	212	-16.3	CCTCTTCCTGCATTCAGCCTTCT	GGATGGTTGGGGGCGGTCGGCGT
hsa_miR_8069	287	-16.2	GCTCTAACTCTGTCCCAATCAGCT	GGATGGTTGGGGGCGGTCGGCGT
hsa_miR_8069	328	-16	ATGCTTAACTTCTCTGGGCCTCA	GGATGGTTGGGGGCGGTCGGCGT
hsa_miR_8072	16	-16.9	CAGCAAACACTGCCCCCGTG	GGCGGCGGGGAGGTAGGCAG
hsa_miR_8077	207	-18.2	GGCATCCTCTTCCTGCATTCAGCC	GGCTGAGTGGGGTTCTGACTCC
hsa_miR_8077	141	-17.7	CCAGAAGGACAGCCCCTCATGCC	GGCTGAGTGGGGTTCTGACTCC
hsa_miR_8081	432	-15.1	GAAACAGAAAGTACATGACCGAT	CTTGAGTCGTGCCTTTCTGAATG
hsa_miR_8082	40	-17.6	CCTGGTGCCATCCTTGGCTCCATTA	TGATGGAGCTGGGAATACTCTG
hsa_miR_8085	203	-23.7	GCCTGGCATCCTCTTCCTG	TGGGAGAGAGGACTGTGAGGC
hsa_miR_8085	283	-16.4	TCCAGCTCTAACTCTGTCCCA	TGGGAGAGAGGACTGTGAGGC
hsa_miR_8089	178	-26.9	ACGCCACCTCTTCCTCAGTGCCCCAGC	CCTGGGGACAGGGGATTGGGGCAG
hsa_miR_8089	328	-22.5	ATGCTTAACTTCTCTGGGCCTCAGT	CCTGGGGACAGGGGATTGGGGCAG
hsa_miR_877_3p	474	-16.4	AAGGGCTGGAAGAAGCAGGGGA	TCCTCTTCTCCCTCCTCCCAG
hsa_miR_877_5p	39	-19.4	TCCTGGTGCCATCCTTGGC	GTAGAGGAGATGGCGCAGGG
hsa_miR_877_5p	199	-17.3	CCCAGCCTGGCATCCTCTTCCTGC	GTAGAGGAGATGGCGCAGGG
hsa_miR_920	183	-21.1	ACCTCTTCCTCAGTGCCCC	GGGGAGCTGTGGAAGCAGTA
hsa_miR_920	14	-16	AGCAGCAAACACTGCCCCC	GGGGAGCTGTGGAAGCAGTA
hsa_miR_92a_1_5p	286	-16.7	AGCTCTAACTCTGTCCCAATCA	AGGTTGGGATCGGTTGCAATGCT
hsa_miR_92b_5p	122	-16.9	GAATGTTCTACTTCCTGTTCCA	AGGGACGGGACGCGGTGCAGTG
hsa_miR_92b_5p	217	-16.3	TCCTGCATTCAGCCTTCTTTTCCG	AGGGACGGGACGCGGTGCAGTG
hsa_miR_92b_5p	199	-15.3	CCCAGCCTGGCATCCTCTTCCT	AGGGACGGGACGCGGTGCAGTG
hsa_miR_937_5p	208	-16.3	GCATCCTCTTCCTGCATTCAG	GTGAGTCAGGGTGGGGCTGG
hsa_miR_939_5p	209	-21.8	CATCCTCTTCCTGCATTCAGCCTTCT	TGGGGAGCTGAGGCTCTGGGGGTG
hsa_miR_939_5p	195	-20.4	GTGCCCCAGCCTGGCATCCTCT	TGGGGAGCTGAGGCTCTGGGGGTG
hsa_miR_939_5p	113	-19.7	TGCCCTTCAGAATGTTCTACTTCCTG	TGGGGAGCTGAGGCTCTGGGGGTG
