# Supplementary material for: CCMAlnc Promotes the Malignance of Colorectal Cancer by Modulating the Interaction Between miR-5001-5p and Its Target mRNA
Source: Front Cell Dev Biol. 2020 Dec 16;8:566932. doi: 10.3389/fcell.2020.566932 (PMC7931267; doi:10.3389/fcell.2020.566932)
Supplement: Supplementary file 8 [file Table_3.PDF]

Table S3 miR-5001-5p target genes

Target gene	Representative transcript	Gene name
GIPC3	ENST00000322315.5	GIPC PDZ domain containing family, member 3
CENPP	ENST00000375587.3	centromere protein P
C1orf95	ENST00000366788.3	chromosome 1 open reading frame 95
B3GALT5	ENST00000380620.4	UDP-Gal:betaGlcNAc beta 1,3-galactosyltransferase, polypeptide 5
WNK2	ENST00000395475.2	WNK lysine deficient protein kinase 2
STK40	ENST00000359297.2	serine/threonine kinase 40
ATG2A	ENST00000421419.2	autophagy related 2A
SZT2	ENST00000562955.1	seizure threshold 2 homolog (mouse)
DENND6B	ENST00000413817.3	DENN/MADD domain containing 6B
MDGA1	ENST00000297153.7	MAM domain containing glycosylphosphatidylinositol anchor 1
C17orf103	ENST00000468196.1	chromosome 17 open reading frame 103
CABP7	ENST00000216144.3	calcium binding protein 7
KCNC1	ENST00000379472.3	potassium voltage-gated channel, Shaw-related subfamily, member 1
GPR20	ENST00000377741.3	G protein-coupled receptor 20
SLC12A4	ENST00000422611.2	solute carrier family 12 (potassium/chloride transporter), member 4
TSPAN14	ENST00000429989.3	tetraspanin 14
MRTO4	ENST00000330263.4	mRNA turnover 4 homolog (S. cerevisiae)
TTYH3	ENST00000258796.7	tweety family member 3
PLXNA4	ENST00000321063.4	plexin A4
OSBP2	ENST00000382310.3	oxysterol binding protein 2
KCNIP3	ENST00000295225.5	Kv channel interacting protein 3, calsenilin
CRTC1	ENST00000338797.6	CREB regulated transcription coactivator 1
LRRC3	ENST00000291592.4	leucine rich repeat containing 3
KIAA0930	ENST00000336156.5	KIAA0930
SYT12	ENST00000393946.2	synaptotagmin XII
TAB1	ENST00000216160.6	TGF-beta activated kinase 1/MAP3K7 binding protein 1
DUSP8	ENST00000397374.3	dual specificity phosphatase 8
TXNRD3NB	ENST00000383572.2	thioredoxin reductase 3 neighbor
MBD3	ENST00000590550.2	methyl-CpG binding domain protein 3
TBL3	ENST00000568546.1	transducin (beta)-like 3
KIF21B	ENST00000332129.2	kinesin family member 21B
TMEM134	ENST00000393877.3	transmembrane protein 134
NXPH3	ENST00000328741.5	neurexophilin 3
ZNF609	ENST00000326648.3	zinc finger protein 609
TBC1D16	ENST00000310924.2	TBC1 domain family, member 16
NSMCE1	ENST00000361439.4	non-SMC element 1 homolog (S. cerevisiae)
PCYT2	ENST00000538936.2	phosphate cytidylyltransferase 2, ethanolamine
GNB1L	ENST00000329517.6	guanine nucleotide binding protein (G protein), beta polypeptide 1-like
HES6	ENST00000409574.1	hairy and enhancer of split 6 (Drosophila)
AC174470.1	ENST00000457257.1	
FAM222B	ENST00000582266.1	family with sequence similarity 222, member B
SYT2	ENST00000367267.1	synaptotagmin II
PSD4	ENST00000441564.3	pleckstrin and Sec7 domain containing 4
LYNX1	ENST00000395192.2	Ly6/neurotoxin 1
GRIK3	ENST00000373091.3	glutamate receptor, ionotropic, kainate 3
TRMT61A	ENST00000389749.4	tRNA methyltransferase 61 homolog A (S. cerevisiae)
SLC9A8	ENST00000361573.2	solute carrier family 9, subfamily A (NHE8, cation proton antiporter 8), member 8
PACSIN1	ENST00000244458.2	protein kinase C and casein kinase substrate in neurons 1
TIFAB	ENST00000537858.1	TRAF-interacting protein with forkhead-associated domain, family member B
MGRN1	ENST00000415496.1	mahogunin ring finger 1, E3 ubiquitin protein ligase
FBXW8	ENST00000455858.2	F-box and WD repeat domain containing 8
PLCXD1	ENST00000381657.2	phosphatidylinositol-specific phospholipase C, X domain containing 1
MGLL	ENST00000434178.2	monoglyceride lipase
POLL	ENST00000370168.3	polymerase (DNA directed), lambda
TMEM127	ENST00000258439.3	transmembrane protein 127
NUDT3	ENST00000607016.1	nudix (nucleoside diphosphate linked moiety X)-type motif 3
NT5C1A	ENST00000235628.1	5'-nucleotidase, cytosolic IA
NECAB3	ENST00000246190.6	N-terminal EF-hand calcium binding protein 3
DDR1	ENST00000446312.1	discoidin domain receptor tyrosine kinase 1
SH3PXD2B	ENST00000311601.5	SH3 and PX domains 2B
P2RY2	ENST00000311131.2	purinergic receptor P2Y, G-protein coupled, 2
ATP2B2	ENST00000352432.4	ATPase, Ca++ transporting, plasma membrane 2
ORAI2	ENST00000356387.2	ORAI calcium release-activated calcium modulator 2
ASB6	ENST00000277459.4	ankyrin repeat and SOCS box containing 6
ARHGEF4	ENST00000392953.3	Rho guanine nucleotide exchange factor (GEF) 4
ZBTB16	ENST00000335953.4	zinc finger and BTB domain containing 16
CABLES1	ENST00000256925.7	Cdk5 and Abl enzyme substrate 1
ZMIZ1	ENST00000334512.5	zinc finger, MIZ-type containing 1
ANKRD33B	ENST00000296657.5	ankyrin repeat domain 33B
ELAVL3	ENST00000359227.3	ELAV like neuron-specific RNA binding protein 3
CLSTN2	ENST00000458420.3	calsyntenin 2
ZDHHC3	ENST00000296127.3	zinc finger, DHHC-type containing 3
RBM28	ENST00000223073.2	RNA binding motif protein 28
WDFY2	ENST00000298125.5	WD repeat and FYVE domain containing 2
RPL28	ENST00000560583.1	ribosomal protein L28
DCPS	ENST00000263579.4	decapping enzyme, scavenger
EHD1	ENST00000320631.3	EH-domain containing 1
SLC8A1	ENST00000406785.2	solute carrier family 8 (sodium/calcium exchanger), member 1
CNKSR3	ENST00000607772.1	CNKSR family member 3
NKIRAS2	ENST00000462043.2	NFKB inhibitor interacting Ras-like 2
TNS4	ENST00000254051.6	tensin 4
GLTPD1	ENST00000343938.4	glycolipid transfer protein domain containing 1
NDUFA4L2	ENST00000556732.1	NADH dehydrogenase (ubiquinone) 1 alpha subcomplex, 4-like 2
MARVELD1	ENST00000285605.6	MARVEL domain containing 1
TMEM234	ENST00000373593.1	transmembrane protein 234

TRIM66	ENST00000299550.6	tripartite motif containing 66
PTPRF	ENST00000372414.3	protein tyrosine phosphatase, receptor type, F
CLEC16A	ENST00000409790.1	C-type lectin domain family 16, member A
SLC46A1	ENST00000440501.1	solute carrier family 46 (folate transporter), member 1
FBXL16	ENST00000397621.1	F-box and leucine-rich repeat protein 16
RAB37	ENST00000392610.1	RAB37, member RAS oncogene family
CNIH2	ENST00000528852.1	cornichon family AMPA receptor auxiliary protein 2
PLA2G2F	ENST00000375102.3	phospholipase A2, group IIF
CYB5R3	ENST00000361740.4	cytochrome b5 reductase 3
XIRP1	ENST00000396251.1	xin actin-binding repeat containing 1
PPM1M	ENST00000296487.4	protein phosphatase, Mg2+/Mn2+ dependent, 1M
FAM219A	ENST00000379089.1	family with sequence similarity 219, member A
ADM2	ENST00000395738.2	adrenomedullin 2
PCDHGA11	ENST00000398587.2	protocadherin gamma subfamily A, 11
KCNJ5	ENST00000529694.1	potassium inwardly-rectifying channel, subfamily J, member 5
PCDHGA5	ENST00000518069.1	protocadherin gamma subfamily A, 5
PCDHGA1	ENST00000517417.1	protocadherin gamma subfamily A, 1
PCDHGA9	ENST00000573521.1	protocadherin gamma subfamily A, 9
PCDHGA8	ENST00000398604.2	protocadherin gamma subfamily A, 8
PCDHGA7	ENST00000518325.1	protocadherin gamma subfamily A, 7
PCDHGA12	ENST00000252085.3	protocadherin gamma subfamily A, 12
PCDHGA6	ENST00000517434.1	protocadherin gamma subfamily A, 6
PCDHGA2	ENST00000394576.2	protocadherin gamma subfamily A, 2
PCDHGA3	ENST00000253812.6	protocadherin gamma subfamily A, 3
PCDHGA10	ENST00000398610.2	protocadherin gamma subfamily A, 10
ZFP41	ENST00000520584.1	ZFP41 zinc finger protein
TOX2	ENST00000372999.1	TOX high mobility group box family member 2
NAT8L	ENST00000331662.3	N-acetyltransferase 8-like (GCN5-related, putative)
PARVG	ENST00000444313.3	parvin, gamma
C11orf21	ENST00000381153.3	chromosome 11 open reading frame 21
CBX7	ENST00000216133.5	chromobox homolog 7
DPYSL5	ENST00000288699.6	dihydropyrimidinase-like 5
DAP	ENST00000230895.6	death-associated protein
ISY1-RAB43	ENST00000418265.1	ISY1-RAB43 readthrough
CD7	ENST00000584284.1	CD7 molecule
CTD-2368P22	ENST00000550135.1	HCG1811579; Uncharacterized protein
PANX2	ENST00000159647.5	pannexin 2
ENTPD2	ENST00000355097.2	ectonucleoside triphosphate diphosphohydrolase 2
SEMA7A	ENST00000261918.4	semaphorin 7A, GPI membrane anchor (John Milton Hagen blood group)
GAS8	ENST00000268699.4	growth arrest-specific 8
HS1BP3	ENST00000304031.3	HCLS1 binding protein 3
NRG1	ENST00000341377.5	neuregulin 1
TMEM92	ENST00000300433.3	transmembrane protein 92
HAAO	ENST00000294973.6	3-hydroxyanthranilate 3,4-dioxygenase
FAM163A	ENST00000341785.4	family with sequence similarity 163, member A
IL1R1	ENST00000410023.1	interleukin 1 receptor, type I
ZBTB7C	ENST00000535628.2	zinc finger and BTB domain containing 7C
SLC17A9	ENST00000370351.4	solute carrier family 17 (vesicular nucleotide transporter), member 9
KCNJ6	ENST00000609713.1	potassium inwardly-rectifying channel, subfamily J, member 6
SHB	ENST00000377707.3	Src homology 2 domain containing adaptor protein B
URM1	ENST00000372850.1	ubiquitin related modifier 1
TMEM151B	ENST00000451188.2	transmembrane protein 151B
CDR2L	ENST00000337231.5	cerebellar degeneration-related protein 2-like
MOCS1	ENST00000373186.4	molybdenum cofactor synthesis 1
TOB2	ENST00000327492.3	transducer of ERBB2, 2
PCDH11X	ENST00000504220.2	protocadherin 11 X-linked
PCDHGB4	ENST00000519479.1	protocadherin gamma subfamily B, 4
PCDHGB1	ENST00000523390.1	protocadherin gamma subfamily B, 1
PCDHGB6	ENST00000520790.1	protocadherin gamma subfamily B, 6
PCDHGB2	ENST00000522605.1	protocadherin gamma subfamily B, 2
PCDHGA4	ENST00000571252.1	protocadherin gamma subfamily A, 4
PCDHGB7	ENST00000398594.2	protocadherin gamma subfamily B, 7
PCDHGB3	ENST00000576222.1	protocadherin gamma subfamily B, 3
CDCP1	ENST00000296129.1	CUB domain containing protein 1
PCDHGC4	ENST00000306593.1	protocadherin gamma subfamily C, 4
PCDHGC3	ENST00000308177.3	protocadherin gamma subfamily C, 3
PCDHGC5	ENST00000252087.1	protocadherin gamma subfamily C, 5
MYO1C	ENST00000359786.5	myosin IC
SLC29A4	ENST00000396872.3	solute carrier family 29 (equilibrative nucleoside transporter), member 4
LRRC4B	ENST00000599957.1	leucine rich repeat containing 4B
UAP1L1	ENST00000360271.3	UDP-N-acteylglucosamine pyrophosphorylase 1-like 1
NT5DC3	ENST00000392876.3	5'-nucleotidase domain containing 3
MVB12B	ENST00000361171.3	multivesicular body subunit 12B
PLCH2	ENST00000449969.1	phospholipase C, eta 2
GRK5	ENST00000392870.2	G protein-coupled receptor kinase 5
IQSEC2	ENST00000375365.2	IQ motif and Sec7 domain 2
KLHL21	ENST00000377663.3	kelch-like family member 21
AGFG2	ENST00000262935.4	ArfGAP with FG repeats 2
ONECUT3	ENST00000382349.4	one cut homeobox 3
TMEM178B	ENST00000565468.1	transmembrane protein 178B
PPARD	ENST00000448077.2	peroxisome proliferator-activated receptor delta
FANCA	ENST00000568369.1	Fanconi anemia, complementation group A
DCAF7	ENST00000310827.4	DDB1 and CUL4 associated factor 7
PHLDB1	ENST00000361417.2	pleckstrin homology-like domain, family B, member 1
TET3	ENST00000409262.3	tet methylcytosine dioxygenase 3
CAPN15	ENST00000219611.2	calpain 15
CCNF	ENST00000397066.4	cyclin F

TEAD1	ENST00000361905.4	TEA domain family member 1 (SV40 transcriptional enhancer factor)
TFE3	ENST00000315869.7	transcription factor binding to IGHM enhancer 3
SLC36A1	ENST00000243389.3	solute carrier family 36 (proton/amino acid symporter), member 1
NGFR	ENST00000172229.3	nerve growth factor receptor
SLC35B4	ENST00000378509.4	solute carrier family 35 (UDP-xylose/UDP-N-acetylglucosamine transporter), member B4
LHPP	ENST00000368839.1	phospholysine phosphohistidine inorganic pyrophosphate phosphatase
PAX2	ENST00000370296.2	paired box 2
WNT3A	ENST00000284523.1	wingless-type MMTV integration site family, member 3A
NFAM1	ENST00000329021.5	NFAT activating protein with ITAM motif 1
ZNRF1	ENST00000335325.4	zinc and ring finger 1, E3 ubiquitin protein ligase
SPTB	ENST00000389721.5	spectrin, beta, erythrocytic
RIMS4	ENST00000372851.3	regulating synaptic membrane exocytosis 4
ELK1	ENST00000247161.3	ELK1, member of ETS oncogene family
WDFY4	ENST00000413659.2	WDFY family member 4
CNTN2	ENST00000331830.4	contactin 2 (axonal)
KBTBD12	ENST00000405256.1	kelch repeat and BTB (POZ) domain containing 12
TMEM184B	ENST00000361906.3	transmembrane protein 184B
KIAA0513	ENST00000566428.1	KIAA0513
GPR144	ENST00000334810.1	G protein-coupled receptor 144
WASF2	ENST00000536657.1	WAS protein family, member 2
GLIS2	ENST00000262366.3	GLIS family zinc finger 2
GPRC5A	ENST00000014914.5	G protein-coupled receptor, family C, group 5, member A
TXLNA	ENST00000373610.3	taxilin alpha
FADS2	ENST00000257261.6	fatty acid desaturase 2
CYP20A1	ENST00000356079.4	cytochrome P450, family 20, subfamily A, polypeptide 1
BCAS3	ENST00000589222.1	breast carcinoma amplified sequence 3
CRAMP1L	ENST00000397412.3	Crm, cramped-like (Drosophila)
HS6ST1	ENST00000259241.6	heparan sulfate 6-O-sulfotransferase 1
LRRC32	ENST00000260061.5	leucine rich repeat containing 32
UQCR11	ENST00000589880.1	ubiquinol-cytochrome c reductase, complex III subunit XI
TFCP2L1	ENST00000263707.5	transcription factor CP2-like 1
ZNF460	ENST00000360338.3	zinc finger protein 460
CHD5	ENST00000262450.3	chromodomain helicase DNA binding protein 5
KCNK5	ENST00000359534.3	potassium channel, subfamily K, member 5
KIF6	ENST00000287152.7	kinesin family member 6
KIF26B	ENST00000366518.4	kinesin family member 26B
CLCN5	ENST00000376088.3	chloride channel, voltage-sensitive 5
GRAP2	ENST00000344138.4	GRB2-related adaptor protein 2
ARHGAP35	ENST00000404338.3	Rho GTPase activating protein 35
ADAP1	ENST00000265846.5	ArfGAP with dual PH domains 1
PQLC1	ENST00000590381.1	PQ loop repeat containing 1
NFASC	ENST00000401399.1	neurofascin
ENGASE	ENST00000579016.1	endo-beta-N-acetylglucosaminidase
COPS8	ENST00000354371.2	COP9 signalosome subunit 8
PPARGC1B	ENST00000309241.5	peroxisome proliferator-activated receptor gamma, coactivator 1 beta
NEURL1B	ENST00000369800.5	neuralized homolog 1B (Drosophila)
GRIN2B	ENST00000609686.1	glutamate receptor, ionotropic, N-methyl D-aspartate 2B
FBLN7	ENST00000331203.2	fibulin 7
FOXN3	ENST00000345097.4	forkhead box N3
SH3BP2	ENST00000356331.5	SH3-domain binding protein 2
SRGAP3	ENST00000383836.3	SLIT-ROBO Rho GTPase activating protein 3
HIF1AN	ENST00000299163.6	hypoxia inducible factor 1, alpha subunit inhibitor
ATG13	ENST00000359513.4	autophagy related 13
ENTPD1	ENST00000371207.3	ectonucleoside triphosphate diphosphohydrolase 1
RALY	ENST00000375114.3	RALY heterogeneous nuclear ribonucleoprotein
C1orf21	ENST00000235307.6	chromosome 1 open reading frame 21
C9orf114	ENST00000361256.5	chromosome 9 open reading frame 114
MRPL12	ENST00000333676.3	mitochondrial ribosomal protein L12
AP5Z1	ENST00000348624.4	adaptor-related protein complex 5, zeta 1 subunit
PPP1R15A	ENST00000200453.5	protein phosphatase 1, regulatory subunit 15A
SPN	ENST00000360121.3	sialophorin
PAPLN	ENST00000381166.3	papilin, proteoglycan-like sulfated glycoprotein
KIF1A	ENST00000320389.7	kinesin family member 1A
RPL14	ENST00000416518.1	ribosomal protein L14
PTTG1IP	ENST00000397887.3	pituitary tumor-transforming 1 interacting protein
ALPK3	ENST00000258888.5	alpha-kinase 3
ZNF346	ENST00000503039.1	zinc finger protein 346
TNFAIP8L2	ENST00000368910.3	tumor necrosis factor, alpha-induced protein 8-like 2
SAMD11	ENST00000342066.3	sterile alpha motif domain containing 11
KIAA1614	ENST00000367588.4	KIAA1614
SLC2A8	ENST00000373360.3	solute carrier family 2 (facilitated glucose transporter), member 8
SLC2A6	ENST00000371897.4	solute carrier family 2 (facilitated glucose transporter), member 6
PKM	ENST00000319622.6	pyruvate kinase, muscle
DFNB31	ENST00000265134.6	deafness, autosomal recessive 31
LDB3	ENST00000542786.1	LIM domain binding 3
XXYLT1	ENST00000310380.6	xyloside xylosyltransferase 1
CINP	ENST00000541568.2	cyclin-dependent kinase 2 interacting protein
KDM6B	ENST00000254846.5	lysine (K)-specific demethylase 6B
SPTBN2	ENST00000529997.1	spectrin, beta, non-erythrocytic 2
AOC3	ENST00000591562.1	amine oxidase, copper containing 3
ACE	ENST00000290866.4	angiotensin I converting enzyme
AC010327.2	ENST00000598855.1	Uncharacterized protein; cDNA FLJ45856 fis, clone OCBBF2025631
C11orf49	ENST00000395460.2	chromosome 11 open reading frame 49
TRAF1	ENST00000373887.3	TNF receptor-associated factor 1
PES1	ENST00000354694.7	pescadillo ribosomal biogenesis factor 1
AL355390.1	ENST00000325811.1	Uncharacterized protein
S100A7A	ENST00000368729.4	S100 calcium binding protein A7A

HAP1	ENST00000393939.2	huntingtin-associated protein 1
AC110781.3	ENST00000402221.1	Protein LOC100128374
GPR39	ENST00000329321.3	G protein-coupled receptor 39
PPP3R2	ENST00000374806.1	protein phosphatase 3, regulatory subunit B, beta
ISX	ENST00000308700.6	intestine-specific homeobox
PRRC2B	ENST00000372249.1	proline-rich coiled-coil 2B
C10orf105	ENST00000441508.2	chromosome 10 open reading frame 105
MFAP4	ENST00000497081.2	microfibrillar-associated protein 4
PER1	ENST00000317276.4	period circadian clock 1
CYB561D1	ENST00000496961.1	cytochrome b561 family, member D1
TMCC3	ENST00000261226.4	transmembrane and coiled-coil domain family 3
MAPK8IP3	ENST00000250894.4	mitogen-activated protein kinase 8 interacting protein 3
OPA3	ENST00000263275.4	optic atrophy 3 (autosomal recessive, with chorea and spastic paraplegia)
PRX	ENST00000291825.7	periaxin
BEGAIN	ENST00000355173.2	brain-enriched guanylate kinase-associated
CDH5	ENST00000341529.3	cadherin 5, type 2 (vascular endothelium)
STAC2	ENST00000333461.5	SH3 and cysteine rich domain 2
CLIP2	ENST00000223398.6	CAP-GLY domain containing linker protein 2
FAM120AOS	ENST00000423591.1	family with sequence similarity 120A opposite strand
LILRA6	ENST00000391735.3	leukocyte immunoglobulin-like receptor, subfamily A (with TM domain), member 6
PPM1H	ENST00000228705.6	protein phosphatase, Mg2+/Mn2+ dependent, 1H
MMP14	ENST00000311852.6	matrix metallopeptidase 14 (membrane-inserted)
CCDC9	ENST00000221922.6	coiled-coil domain containing 9
MEX3B	ENST00000558133.1	mex-3 RNA binding family member B
C1orf64	ENST00000329454.2	chromosome 1 open reading frame 64
SEC14L5	ENST00000251170.7	SEC14-like 5 (S. cerevisiae)
ABR	ENST00000302538.5	active BCR-related
G6PC	ENST00000253801.2	glucose-6-phosphatase, catalytic subunit
BRSK2	ENST00000382179.1	BR serine/threonine kinase 2
CNP	ENST00000393892.3	2',3'-cyclic nucleotide 3' phosphodiesterase
SLMO1	ENST00000592149.1	slowmo homolog 1 (Drosophila)
KLLN	ENST00000445946.3	killin, p53-regulated DNA replication inhibitor
WDR48	ENST00000302313.5	WD repeat domain 48
OVOL1	ENST00000335987.3	ovo-like 1(Drosophila)
WNT7B	ENST00000339464.4	wingless-type MMTV integration site family, member 7B
NR6A1	ENST00000487099.2	nuclear receptor subfamily 6, group A, member 1
PPIL2	ENST00000406385.1	peptidylprolyl isomerase (cyclophilin)-like 2
DPP9	ENST00000594671.1	dipeptidyl-peptidase 9
RP11-156E8.	ENST00000607453.1	
LDLRAD2	ENST00000344642.2	low density lipoprotein receptor class A domain containing 2
F2RL3	ENST00000248076.3	coagulation factor II (thrombin) receptor-like 3
NTRK2	ENST00000395882.1	neurotrophic tyrosine kinase, receptor, type 2
ESPN	ENST00000377828.1	espin
RAF1	ENST00000251849.4	v-raf-1 murine leukemia viral oncogene homolog 1
TNFRSF14	ENST00000355716.4	tumor necrosis factor receptor superfamily, member 14
GALNT10	ENST00000297107.6	UDP-N-acetyl-alpha-D-galactosamine:polypeptide N-acetylgalactosaminyltransferase 10 (GalNAc-T10)
KIAA1045	ENST00000242315.3	KIAA1045
CCDC13	ENST00000310232.6	coiled-coil domain containing 13
CACNA1E	ENST00000526775.1	calcium channel, voltage-dependent, R type, alpha 1E subunit
EXOSC2	ENST00000372358.5	exosome component 2
TMEM216	ENST00000334888.5	transmembrane protein 216
METAP1D	ENST00000315796.4	methionyl aminopeptidase type 1D (mitochondrial)
SUFU	ENST00000369902.3	suppressor of fused homolog (Drosophila)
P4HA2	ENST00000401867.1	prolyl 4-hydroxylase, alpha polypeptide II
MAPK3	ENST00000403394.1	mitogen-activated protein kinase 3
ACVR1B	ENST00000257963.4	activin A receptor, type IB
RMND5B	ENST00000515098.1	required for meiotic nuclear division 5 homolog B (S. cerevisiae)
SPRED3	ENST00000587013.1	sprouty-related, EVH1 domain containing 3
NCOR1	ENST00000268712.3	nuclear receptor corepressor 1
CDC14B	ENST00000265659.2	cell division cycle 14B
JPH3	ENST00000284262.2	junctophilin 3
C15orf39	ENST00000567617.1	chromosome 15 open reading frame 39
VDR	ENST00000395324.2	vitamin D (1,25- dihydroxyvitamin D3) receptor
PYGB	ENST00000216962.4	phosphorylase, glycogen; brain
CLCN2	ENST00000423355.2	chloride channel, voltage-sensitive 2
PHF19	ENST00000373896.3	PHD finger protein 19
DNAH17	ENST00000585328.1	dynein, axonemal, heavy chain 17
HGSNAT	ENST00000379644.4	heparan-alpha-glucosaminide N-acetyltransferase
ABCB8	ENST00000356058.4	ATP-binding cassette, sub-family B (MDR/TAP), member 8
TOR1A	ENST00000351698.4	torsin family 1, member A (torsin A)
RGSL1	ENST00000294854.8	regulator of G-protein signaling like 1
GATSL2	ENST00000426327.3	GATS protein-like 2
FAAH	ENST00000243167.8	fatty acid amide hydrolase
TBC1D24	ENST00000567020.1	TBC1 domain family, member 24
ZNF573	ENST00000590414.2	zinc finger protein 573
CAMKV	ENST00000296471.7	CaM kinase-like vesicle-associated
FAM83A	ENST00000518448.1	family with sequence similarity 83, member A
PLXDC2	ENST00000377252.4	plexin domain containing 2
RARA	ENST00000425707.3	retinoic acid receptor, alpha
CBX2	ENST00000310942.4	chromobox homolog 2
CLCN6	ENST00000312413.6	chloride channel, voltage-sensitive 6
PRIMA1	ENST00000393140.1	proline rich membrane anchor 1
RASSF6	ENST00000307439.5	Ras association (RalGDS/AF-6) domain family member 6
CARD11	ENST00000396946.4	caspase recruitment domain family, member 11
WDR52	ENST00000393845.2	WD repeat domain 52
PGPEP1	ENST00000597431.2	pyroglutamyl-peptidase I
ZNF275	ENST00000370251.3	zinc finger protein 275

PDGFRB	ENST00000261799.4	platelet-derived growth factor receptor, beta polypeptide
POR	ENST00000394893.1	P450 (cytochrome) oxidoreductase
SYNJ1	ENST00000357345.3	synaptojanin 1
GPR107	ENST00000372410.3	G protein-coupled receptor 107
GAS2L1	ENST00000360113.2	growth arrest-specific 2 like 1
PARVA	ENST00000334956.8	parvin, alpha
GM2A	ENST00000357164.3	GM2 ganglioside activator
MYL9	ENST00000279022.2	myosin, light chain 9, regulatory
GNAL	ENST00000334049.6	guanine nucleotide binding protein (G protein), alpha activating activity polypeptide, olfactory type
ARMC5	ENST00000457010.2	armadillo repeat containing 5
PPP1R11	ENST00000376773.1	protein phosphatase 1, regulatory (inhibitor) subunit 11
TMOD2	ENST00000249700.4	tropomodulin 2 (neuronal)
KCNC3	ENST00000376959.2	potassium voltage-gated channel, Shaw-related subfamily, member 3
LANCL2	ENST00000254770.2	LanC lantibiotic synthetase component C-like 2 (bacterial)
DUSP7	ENST00000495880.1	dual specificity phosphatase 7
HIP1	ENST00000336926.6	huntingtin interacting protein 1
GRM4	ENST00000538487.2	glutamate receptor, metabotropic 4
DDX11	ENST00000251758.5	DEAD/H (Asp-Glu-Ala-Asp/His) box helicase 11
FAM46B	ENST00000289166.5	family with sequence similarity 46, member B
CAPN5	ENST00000531028.1	calpain 5
GRAMD1B	ENST00000529750.1	GRAM domain containing 1B
PLEKHM3	ENST00000427836.2	pleckstrin homology domain containing, family M, member 3
RASSF4	ENST00000374417.2	Ras association (RalGDS/AF-6) domain family member 4
SFXN5	ENST00000410065.1	sideroflexin 5
PADI2	ENST00000375486.4	peptidyl arginine deiminase, type II
KLF2	ENST00000248071.5	Kruppel-like factor 2 (lung)
PAOX	ENST00000368539.4	polyamine oxidase (exo-N4-amino)
KCNB1	ENST00000371741.4	potassium voltage-gated channel, Shab-related subfamily, member 1
LIMK2	ENST00000331728.4	LIM domain kinase 2
NEURL	ENST00000369780.4	neuralized homolog (Drosophila)
DGCR2	ENST00000545799.1	DiGeorge syndrome critical region gene 2
EMX1	ENST00000258106.6	empty spiracles homeobox 1
RGP1	ENST00000378078.4	RGP1 retrograde golgi transport homolog (S. cerevisiae)
WIPF2	ENST00000323571.4	WAS/WASL interacting protein family, member 2
UNC5B	ENST00000335350.6	unc-5 homolog B (C. elegans)
ATP2A2	ENST00000395494.2	ATPase, Ca++ transporting, cardiac muscle, slow twitch 2
TMEM132B	ENST00000299308.3	transmembrane protein 132B
CMTM4	ENST00000394106.2	CKLF-like MARVEL transmembrane domain containing 4
PPP1R12B	ENST00000608999.1	protein phosphatase 1, regulatory subunit 12B
MTHFR	ENST00000376592.1	methylenetetrahydrofolate reductase (NAD(P)H)
C19orf24	ENST00000409293.4	chromosome 19 open reading frame 24
CCDC144A	ENST00000443444.2	coiled-coil domain containing 144A
ANKRD52	ENST00000267116.7	ankyrin repeat domain 52
COL1A1	ENST00000225964.5	collagen, type I, alpha 1
MMP17	ENST00000535291.1	matrix metallopeptidase 17 (membrane-inserted)
ZNF276	ENST00000289816.5	zinc finger protein 276
MEGF6	ENST00000294599.4	multiple EGF-like-domains 6
TMPRSS4	ENST00000534111.1	transmembrane protease, serine 4
IQCE	ENST00000402050.2	IQ motif containing E
TRAF3	ENST00000560371.1	TNF receptor-associated factor 3
RPTOR	ENST00000306801.3	regulatory associated protein of MTOR, complex 1
MYO18A	ENST00000354329.4	myosin XVIIIA
SLFN12	ENST00000394562.1	schlafen family member 12
LANCL3	ENST00000378621.3	LanC lantibiotic synthetase component C-like 3 (bacterial)
ONECUT2	ENST00000491143.2	one cut homeobox 2
GBX2	ENST00000551105.1	gastrulation brain homeobox 2
NETO2	ENST00000562435.1	neuropilin (NRP) and tolloid (TLL)-like 2
LIMD1	ENST00000273317.4	LIM domains containing 1
RAD54L2	ENST00000409535.2	RAD54-like 2 (S. cerevisiae)
DLG2	ENST00000398309.2	discs, large homolog 2 (Drosophila)
PCGF3	ENST00000362003.5	polycomb group ring finger 3
ELFN2	ENST00000402918.2	extracellular leucine-rich repeat and fibronectin type III domain containing 2
PIP5K1C	ENST00000335312.3	phosphatidylinositol-4-phosphate 5-kinase, type I, gamma
USP30	ENST00000257548.5	ubiquitin specific peptidase 30
PKIA	ENST00000396418.2	protein kinase (cAMP-dependent, catalytic) inhibitor alpha
MXD4	ENST00000337190.2	MAX dimerization protein 4
LONRF3	ENST00000304778.7	LON peptidase N-terminal domain and ring finger 3
SNX33	ENST00000308527.5	sorting nexin 33
BRD3	ENST00000303407.7	bromodomain containing 3
RAPGEF3	ENST00000405493.2	Rap guanine nucleotide exchange factor (GEF) 3
RAB6B	ENST00000285208.4	RAB6B, member RAS oncogene family
BCAR1	ENST00000162330.5	breast cancer anti-estrogen resistance 1
WDR91	ENST00000344400.5	WD repeat domain 91
SHH	ENST00000297261.2	sonic hedgehog
ANKRD9	ENST00000559651.1	ankyrin repeat domain 9
UMPS	ENST00000232607.2	uridine monophosphate synthetase
MCF2L2	ENST00000328913.3	MCF.2 cell line derived transforming sequence-like 2
NRDE2	ENST00000354366.3	NRDE-2, necessary for RNA interference, domain containing
RHOBTB2	ENST00000251822.6	Rho-related BTB domain containing 2
RIMS3	ENST00000372684.3	regulating synaptic membrane exocytosis 3
RHBDL3	ENST00000269051.4	rhomboid, veinlet-like 3 (Drosophila)
STAMBP	ENST00000394070.2	STAM binding protein
PMPCA	ENST00000371717.3	peptidase (mitochondrial processing) alpha
TIMP4	ENST00000287814.4	TIMP metallopeptidase inhibitor 4
PSMF1	ENST00000335877.6	proteasome (prosome, macropain) inhibitor subunit 1 (PI31)
APOA1	ENST00000375320.1	apolipoprotein A-I
MTG1	ENST00000317502.6	mitochondrial ribosome-associated GTPase 1

RP11-108K1[illegible]	ENST00000468317.2	Mitochondrial GTPase 1
FAM69B	ENST00000371692.4	family with sequence similarity 69, member B
SF3B3	ENST00000302516.5	splicing factor 3b, subunit 3, 130kDa
POC1A	ENST00000296484.2	POC1 centriolar protein A
FGFR1OP	ENST00000366847.4	FGFR1 oncogene partner
MAP2K2	ENST00000262948.5	mitogen-activated protein kinase kinase 2
CRISPLD2	ENST00000262424.5	cysteine-rich secretory protein LCCL domain containing 2
ECHDC3	ENST00000379215.4	enoyl CoA hydratase domain containing 3
NCKAP1	ENST00000361354.4	NCK-associated protein 1
TSPYL1	ENST00000368608.3	TSPY-like 1
ULK2	ENST00000395544.4	unc-51 like autophagy activating kinase 2
BAG1	ENST00000472232.3	BCL2-associated athanogene
CAPZB	ENST00000375142.1	capping protein (actin filament) muscle Z-line, beta
FHL2	ENST00000409177.1	four and a half LIM domains 2
POTED	ENST00000299443.5	POTE ankyrin domain family, member D
MAGEB10	ENST00000356790.2	melanoma antigen family B, 10
NLRP9	ENST00000332836.2	NLR family, pyrin domain containing 9
