# Supplementary material for: CCMAlnc Promotes the Malignance of Colorectal Cancer by Modulating the Interaction Between miR-5001-5p and Its Target mRNA
Source: Front Cell Dev Biol. 2020 Dec 16;8:566932. doi: 10.3389/fcell.2020.566932 (PMC7931267; doi:10.3389/fcell.2020.566932)
Supplement: Supplementary file 9 [file Data_Sheet_1.DOCX]

**Figure S1.** The relative levels of ENSG00000224879 in normal colon epithelial cell and eight CRC cell lines.

**Figure S2.** CCMAlnc promotes the proliferation and invasion of CRC cells; (A) Relative expression of ENSG00000272502 after transfected of ENSG00000272502 siRNA in SW480 cells; (B) Relative expression of ENSG00000237721 after transfected of ENSG00000237721 siRNA in DLD-1 cells; (C) Relative expression of ENSG00000254814 after transfected of ENSG00000254814 siRNA1 and siRNA2 in HT29 cells; (D) Matrigel invasion assay was performed in SW480 cells after transfection of control siRNA and ENSG00000272502 siRNA, respectively; (E) The expression of ENSG00000237721 when transfected with ENSG00000237721 siRNA in HT29 cells. (F-G) Relative expression of CCMAlnc after transfected of pcDNA3.1-CCMAlnc in (F)SW1116 cells and (G)SW480 cells.

**Figure S3.** Identifcation of miR-5001-5p as a target of CCMAlnc. (A-B) The expression of candidate miRNAs after transfected with control mimics and relevant miRNA mimics in (A)DLD-1 and (B)HT29cells.

**Figure S4.** The noncoding capability of CCMAlnc.
